# Early gene expression changes in spinal cord from SOD1^G93A^ Amyotrophic Lateral Sclerosis animal model

**DOI:** 10.3389/fncel.2013.00216

**Published:** 2013-11-18

**Authors:** Gabriela P. de Oliveira, Chrystian J. Alves, Gerson Chadi

**Affiliations:** Department of Neurology, Neuroregeneration Center, University of São Paulo School of MedicineSão Paulo, Brazil

**Keywords:** ALS, SOD1^G93A^, pre-symptomatic, spinal cord, microarray, laser microdissection, astrocytes

## Abstract

Amyotrophic Lateral Sclerosis (ALS) is an adult-onset and fast progression neurodegenerative disease that leads to the loss of motor neurons. Mechanisms of selective motor neuron loss in ALS are unknown. The early events occurring in the spinal cord that may contribute to motor neuron death are not described, neither astrocytes participation in the pre-symptomatic phases of the disease. In order to identify ALS early events, we performed a microarray analysis employing a whole mouse genome platform to evaluate the gene expression pattern of lumbar spinal cords of transgenic SOD1^G93A^ mice and their littermate controls at pre-symptomatic ages of 40 and 80 days. Differentially expressed genes were identified by means of the Bioconductor packages Agi4×44Preprocess and limma. FunNet web based tool was used for analysis of over-represented pathways. Furthermore, immunolabeled astrocytes from 40 and 80 days old mice were submitted to laser microdissection and RNA was extracted for evaluation of a selected gene by qPCR. Statistical analysis has pointed to 492 differentially expressed genes (155 up and 337 down regulated) in 40 days and 1105 (433 up and 672 down) in 80 days old ALS mice. KEGG analysis demonstrated the over-represented pathways tight junction, antigen processing and presentation, oxidative phosphorylation, endocytosis, chemokine signaling pathway, ubiquitin mediated proteolysis and glutamatergic synapse at both pre-symptomatic ages. *Ube2i* gene expression was evaluated in astrocytes from both transgenic ages, being up regulated in 40 and 80 days astrocytes enriched samples. Our data points to important early molecular events occurring in pre-symptomatic phases of ALS in mouse model. Early SUMOylation process linked to astrocytes might account to non-autonomous cell toxicity in ALS. Further studies on the signaling pathways presented here may provide new insights to better understand the events triggering motor neuron death in this devastating disorder.

## Introduction

Amyotrophic Lateral Sclerosis (ALS) is a fast disabling neurodegenerative disease characterized by upper and lower motor neuron loss of motor cortex, brainstem, and spinal cord leading to respiratory insufficiency and death (Turner et al., 2013). The incidence of ALS ranges from 1.7 to 2.3 cases per 100,000 population per year worldwide (Beghi et al., 2006). The mechanisms underlying neurodegeneration in ALS are multifactorial, and seem to involve neurons and non-neuronal cells (Boillee et al., 2006a,b; Yamanaka et al., 2008; Wang et al., 2011a) as well as several molecular pathways (Boillee et al., 2006a; Ferraiuolo et al., 2011b; Kiernan et al., 2011; Usuki et al., 2012). Approximately 5% of ALS cases are familial, and 20% of these have been linked to mutations in Cu/Zn superoxide dismutase 1 (SOD1) (Rosen et al., 1993; Andersen and Al-Chalabi, 2011). The first symptoms define the beginning of the clinical phase of the diagnosed cases of the more prevalent sporadic forms, consisting in muscle atrophy, weakness, fasciculations, and spasticity (Brooks et al., 2000). There is a lack of pathological studies on post mortem spinal cord from ALS patients that could add information about the triggering, initial time of motor neuron death and mechanisms of the disease. In fact, Fischer et al. (2004) reported the post-mortem evaluation in a patient with a short history of ALS, whose electromyography showed signs of acute and chronic denervation, coming out with an unexpected die without peripheral and central motor neuron death together with autolytic changes and a little axonal degeneration. Histological evaluations at the neuromuscular junctions and also electrophysiological analysis at the peripheral nerves in ALS patients have allowed authors to claim that motor neuron death correlates to the begging of clinical classical symptoms (Veugelers et al., 1996; Liu et al., 2013).

As the majority of familial ALS cases are linked to the mutations in SOD1 gene (Dion et al., 2009), transgenic mice expressing human mutant SOD1 (mSOD1) developing age-dependent clinical and pathological features of human ALS are current largely employed in the physiopathological studies of the disorder (Turner and Talbot, 2008). Using this mouse model, we previously described early behavior and electrophysiological alterations, prior the classical neurological symptoms and the beginning of motor neuron death (Alves et al., 2011). In fact, several early events demonstrated in animal models seemed to precede the neuronal death, remarkably the activation of glial cells (microglia and astrocytes) close to motor neurons (Graber et al., 2010; Wang et al., 2011b; Gerber et al., 2012), retraction of motor neuron fibers and neuromuscular junction displacement (Fischer et al., 2004; De Winter et al., 2006; Narai et al., 2009). It is still unknown whether the most claimed pathogenic processes for ALS, for instance oxidative stress, mitochondrial and neurofilament dysfunction, excitotoxicity, inflammation, non-autonomous cell toxicity, protein misfolding and abnormal RNA processing (Rothstein et al., 1992; Bergeron et al., 1994; Boillee et al., 2006a; Lemmens et al., 2010; Bendotti et al., 2012; Richardson et al., 2013) are taking place at the pre-symptomatic period of the disease.

The central question in understanding ALS facing therapeutic target development involves a further knowledge about the toxic mechanisms that trigger motor neuron death (Boillee et al., 2006a). Until scientific technology approaches do not overstep ethical limitations of clinical studies, the mutant SOD1-expressing mouse model may offer opportunity for a detailed analysis of intra and intercellular signaling-related to motor neuron toxicity.

The profiling of gene expression using different platforms have been largely employed in the ALS model in several stages of the disease course (Olsen et al., 2001; Dangond et al., 2004; Malaspina and De Belleroche, 2004; Jiang et al., 2005; Perrin et al., 2005; Ferraiuolo et al., 2007, 2011a; Yamamoto et al., 2007; Offen et al., 2009; Brockington et al., 2010; D'arrigo et al., 2010; Guipponi et al., 2010; Saris et al., 2013b; Yu et al., 2013), including the early symptomatic phase (Olsen et al., 2001; Yoshihara et al., 2002; Ferraiuolo et al., 2007; Yu et al., 2013), however, there is a lack of information on differential gene expression taking place before classical clinical symptoms (Olsen et al., 2001; Yoshihara et al., 2002; Ferraiuolo et al., 2007; Guipponi et al., 2010). Olsen et al. (2001) inaugurated that issue by looking at patterns of gene expression from SOD1^G93A^ spinal cord by means of a murine restricted platform of oligonucleotide microarray and by describing negligible changes in the transcript profile at the pre-symptomatic phases. Other authors that have examined gene profiling in pre-symptomatic phases of ALS disease employed restricted platforms of cDNA arrays, used animals with an uncommon symptom onset (Yoshihara et al., 2002; Guipponi et al., 2010) or evaluated gene profiling in specific spinal cord cells (Ferraiuolo et al., 2007, 2011a). Authors have encountered gene expressions related to inflammation, apoptosis, oxidative stress, ATP biosynthesis, myelination, axonal transport as candidates of biological processes taking place in the pre-symptomatic periods of ALS.

By means of a high-density oligonucleotide microarrays linked to specific tools capable to identify enriched pathways, the aim of this work was to identify early molecular changes in the pre-symptomatic stage in the spinal cord of the SOD1^G93A^ mouse model. The data showed important alterations at early 40 days pre-symptomatic period of disease and in 80 days old pre-symptomatic mice.

## Materials and methods

### Samples

Specific pathogen-free male SOD1^G93A^ mice of preclinical 40 and 80 days old mice and their age-paired non-transgenic wild-type controls, 20–25 g body weight, from University of São Paulo Medical School (São Paulo, Brazil) were used in the experiments. A total of 5 animals were used in each group in microarray experiments, while in the verification experiments by quantitative polymerase chain reaction (qPCR) and laser microdissection, each group was comprised for 6 and 3 different animals, respectively. Animals were kept under standardized lighting conditions (lights on at 7:00 h and off at 19:00 h), at a constant temperature of 23°C and with free access to food pellets and tap water. The colony was derived from Jackson Laboratories (Bar Harbor, ME, USA) from G93A mutant mice with 25 ± 1.5 copies of the human SOD1 transgene (Gurney, 1994). Mouse identification (SOD^G93A^ or WT) in our colony was performed by genotyping (Scorisa et al., 2010). Animals were killed by decapitation and their lumbar spinal cords were collected for molecular analysis. The study was conducted according protocols approved by the Animal Care and Use of Ethic Committee at the University of São Paulo and in accordance with the Guide for Care and Use of Laboratory Animals adopted by the National Institutes of Health.

### RNA extraction

Total RNA was isolated using the MiniSpin kit for RNA extraction (GE Healthcare, USA) according to the manufacturer's instructions. RNA quantity and integrity were assessed by spectrophotometry (Nanodrop, Thermo Scientific, USA) and microfluidics—based electrophoresis (Agilent 2100 Bioanalyzer, Agilent Technologies, USA), respectively. RNA samples with OD 260/280 of approximately 2.0 and RIN > 7.0 were used for microarray experiments and qPCR. A pool of RNAs from neonatal organs (heart, kidney, liver) was employed as reference sample. A representative eletropherogram from Bioanalyzer evaluation of RNA integrity is shown in supplementary material (Figure S1).

### Microarray experiments

For samples and reference, respectively, 250 and 500 ng of RNA were reverse transcribed by the Low-input RNA Linear Amplification Kit (Agilent Technologies) and then transcribed to Cy3-labeled (samples) or Cy5-labeled (reference) cRNA according to the manufacturer. The labeled cRNA was purified (Minispin kit, GE Life Sciences), and the dye content and concentration of cRNA were measured by a NanoDrop ND-1000 spectrophotometer (Thermo Scientific). A total of 850 ng of Cy3-labeled cRNA was hybridized together with the same amount of Cy5-labeled reference to Whole Mouse Genome Oligo 4 × 44 K microarrays overnight at 65°C, and then the slides were washed and treated with Stabilizing and Drying Solution (Agilent Technologies) and scanned by Agilent Microarray Scanner. All steps were performed according to the manufacturer (Agilent Technologies).

The raw data from hybridizations and experimental conditions are available on the Gene Expression Omnibus website under accession number GSE50642.

### Data analyses

The Feature Extraction Software v9.1.3.1 (Agilent Technologies) was used to extract and analyze the assay signals and subsequently determine the signal-to-noise ratios from the microarray images. Microarrays without enough quality were taken out from further analysis. The analysis proceeded with 4 samples for each group. Microarray raw data (.txt files) were imported into R v. 3.0.1 (Team RDC, 2012) and analyzed with the Bioconductor (Gentleman et al., 2004) packages Agi4×44PreProcess and limma (Smyth, 2005). Briefly, after quality check, the microarray probes were filtered and their median foreground intensity was normalized within and between arrays according to Agi4×44Preprocess and limma user guides, respectively. Finally, the probes were tested for differential expression using a linear model followed by Bayes moderated *t*-test (Smyth, 2005) for the comparisons of interest. Genes with nominal *p* < 0.05 were accepted to be differentially expressed and further considered in the analysis.

### FunNet analysis

In order to further identify over-represented pathways and biological process, the lists with differentially expressed genes for both 40 and 80 days old mice were split into lists of up and down regulated genes and submitted to FunNet web based tool (Functional Analysis of Transcriptional Networks), using Kyoto Encyclopedia of Genes and Genomes (KEGG) and Gene Ontology (GO) annotations (Prifti et al., 2008).

### Laser microdissection of astrocytes

The lumbar spinal cord of mice were rapidly removed and immediately frozen in ice cold isopentane at −45°C and stored at −80°C until use. The labeling procedure was performed as described previously (De Oliveira et al., 2009) and modified according to our experience. Frozen sections (5μm) were rapidly defrosted for 30 s and fixed with ice cold acetone, for 3 min. Sections were then incubated during 3 min in phosphate buffered saline (PBS) containing 3% Triton X-100 and then incubated with primary antibody, a polyclonal rabbit anti-glial fibrillary acidic protein (GFAP; Dako Cytomation; 1:100) diluted in 0.3% Triton X-100 containing 1% BSA for 5 min. Sections were then washed in PBS for 3 times of 15 s and then incubated with texas red-conjugated goat-anti-rabbit secondary antibody, in the same diluent than primary antibody, in a final concentration of 1:50 during 5 min in the dark and at room temperature. Sections were rinsed carefully three times with PBS for 15 s and immediately submitted to laser microdissection.

Around 200 astrocytes were isolated from each 40 and 80 days old mice lumbar spinal cords using P.A.L.M. Microlaser Technologies (Zeiss). RNA was extracted using PicoPure RNA isolation kit (Arcturus) and linear amplification of RNA was performed following Eberwine's procedure (Van Gelder et al., 1990) using the RiboampHSplus kit (Arcturus) according to the manufacturer's protocol. The quantity (NanoDrop 1000 Spectrophotometer) and quality (Agilent 2100 bioanalyser, RNA 6000 Pico LabChip) of amplified RNA was analyzed as described above. Also, the astrocytes enriched samples were submitted to PCRs in order to access contamination from other cell types. Protocol and results of astrocyte samples enrichment are presented in the supplementary material (Figure S2).

## Quantitative PCR

A proportion of genes identified as differentially expressed were selected for verification by qPCR, on the basis of robust microarray data confirming differential gene expression. The genes were chosen for verification based on their possible involvement in ALS related mechanisms. Verification addresses the possibility of false positive microarray signals, due to cross-hybridization with related genes, concern about the accuracy of array probe sets, and uncertainty about the hybridization kinetics of multiple reactions occurring on the miniature scale of an array chip. The qPCR verification of microarray results were performed on independent sample, as described above. cDNA was synthesized from 1 μg of total RNA treated with DNAse by a reverse transcription reagent kit (Applied Biosystems Life Technologies) according to manufacturer. qPCR reactions were carried out in duplicate with 40 ng cDNA, the DyNAmo ColorFlash SYBR Green qPCR kit (Thermo Scientific, USA) and 400 nM of each primer in a final volume reaction of 20 μl, by using the PikoReal Real-Time PCR System (Thermo Scientific). The information for SYBR primers can be found in Table 1. For astrocytes enriched samples, 1 μg of amplified RNA was reverse transcribed to cDNA by a reverse transcription reagent kit (Applied Biosystems Life Technologies) modified from original protocol in order to improve efficiency. Briefly, Oligo(dT)_16_ primer was added to samples and incubated at 70°C during 5 min, then the other required reagents, such as reaction buffer, MgCl_2_, dNTPs, RNAse inhibitor, in the same concentrations than manufacturer protocol, and 156,25 U of Reverse transcriptase (Multiscribe), were added to reaction and incubated at 37°C for 60 min followed by 95°C for 5 min. qPCR reactions were carried out in duplicate using Taqman master mix and the following assays were used: *Ube2i* (Mm04243971_g1) and *Gapdh* (Mm99999915_g1).

**Table 1:** **Information for primers used in SYBR qPCR experiments of 40 and 80 days old pre-symptomatic SOD1^G93A^ and wild-type mice**.

**Gene ID**	**Primer sequences (5′-3′)**	**Amplicon (bp)**
*Glg1*	F: GAGTGAGATTGCAGCCAGAG	143
	R:CAGGATGTAGTTCTTTGAGGGAG	
*Aqp4*	F: GCTCGATCTTTTGGACCCG	112
	R: AGACATACTCATAAAGGGCACC	
*Calca*	F: TGCAGATGAAAGCCAGGG	149
	R: CTTCACCACACCTCCTGATC	
*Eef2*	F: CATGTTTGTGGTCAAGGCATAC	141
	R:TTGTCAAAAGGATCCCCAGG	
*Nsg1*	F: AAGTGTACAAGTATGACCGCG	128
	R: GACAGTGTAAAATTTCTCCCGG	
*Syt10*	F: AGACCATTGGAACGAGATGC	148
	R: TGGAGGCTTTTATGGTGTGG	
**NORMALYZER**		
*Gapdh*	F: GAGTAAGAAACCCTGGACCAC	109
	R: TCTGGGATGGAAATTGTGAGG	

The Glg1, Aqp4, Calca genes and Eef2, Nsg1, Syt10 genes were verified in 40 and 80 days mice, respectively, according to microarray results.

For SYBR reactions the cycling was composed by an initial denaturation at 95°C for 10 min, templates were amplified by 40 cycles of 95°C for 15 s and 60°C for 30 s. A dissociation curve was then generated to ensure amplification of a single product, and absence of primer dimers. For each primer pair, a standard curve was generated to determine the efficiency of the PCR reaction over a range of template concentrations from 0.032 ng/μ l to 20 ng/μ l, using cDNA synthesized from mouse reference RNA. The efficiency for each set of primers was 100 ± 5%. For Taqman reactions, cycling was composed by an initial step of 50°C for 2 min, followed by denaturation at 95°C for 10 min, templates were amplified by 40 cycles of 95°C for 15 s and 60°C for 1 min. Gene expressions, normalized to *Gapdh*, could be determined using the ΔΔCt mathematical model (ABI PRISM 7700 Sequence Detection System protocol; Applied Biosystems). One-tailed unpaired *t*-test was used to determine the statistical significance of any differences in gene expression [GraphPad (San Diego, CA) Prism 5]. *Gapdh* was chosen as a housekeeping gene to normalize the qPCR values because the microarray analysis showed that its expression was stable across samples.

## Results

### General features of differential gene expression between SOD1^G93A^ and wild-type mice

Statistical analysis has pointed to 492 differentially expressed genes at the lumbar region of 40 days SOD1^G93A^, compared to the age matched wild-type mice, being 155 up and 337 down regulated genes, respectively, while 1105 genes were found differentially expressed by 80 days old ALS mice compared to age matched controls, being 433 up and 672 down regulated genes, respectively. The whole list with differentially expressed gene for both age mice can be found in Tables S1 and S2 in the Supplementary material. Of interest, among differentially expressed genes, 66 are common to both ages; they are presented in the Table 2.

**Table 2:** **Differentially expressed genes common to both gene lists of 40 and 80 days old pre-symptomatic SOD1^G93A^ and wild-type mice**.

**Gene symbol**	**Fold change 40 days**	**Fold change 80 days**
*Ocel1*	−1.68	−1.92
*Fam32a*	−1.45	−1.61
*Trim37*	1.22	−1.34
*Lsm6*	−1.3	−1.29
*Map1a*	1.2	−1.15 and −1.28
*Bmpr2*	1.21	−1.26
*Eif3j2*	1.29	−1.26
*Foxn3*	1.28	−1.25
*Plekha5*	1.17	−1.25
*Rfxank*	−1.12	−1.22
*Malat1*	1.1	−1.21
*Ddx6*	1.3	−1.19
*Huwe1*	1.12	−1.19
*Snx27*	1.19	−1.19
*Srrm3*	1.18	−1.19
*Dzip1*	1.14	−1.17
*Hook3*	1.14	−1.17
*Nemf*	1.15	−1.16
*Pdlim5*	1.09	−1.16
*Plvap*	−1.14	−1.16
*Thrap3*	1.15	−1.16
*Srsf11*	−1.1	−1.15
*Azin1*	1.12	−1.14
*Eif5b*	1.19	−1.14
*Hspa4*	1.1	−1.14
*Kras*	1.19	−1.14
*Sp4*	1.12	−1.14
*Tusc3*	−1.1	−1.14
*Vegfa*	1.12	−1.14
*Fam133b*	1.09	−1.13
*Fam81a*	1.12	−1.13
*Prkrir*	−1.1	−1.13
*Ptrf*	−1.3	−1.13
*6330411E07Rik*	1.09	−1.12
*Gria4*	1.18	−1.12
*Zfp866*	1.07	−1.12
*Marc-2*	−1.07	−1.11
*Hadh*	1.12	−1.11
*Mtf2*	1.1	−1.11
*Dhps*	−1.09	−1.1
*Nsd1*	1.12	−1.1
*Mier1*	1.13	−1.09
*U2surp*	1.13	−1.09
*2610507B11Rik*	1.07	1.1
*Ncam1*	1.17	1.12
*Rtn1*	−1.17	1.12
*Chd5*	1.12	1.13
*Dhcr7*	−1.08	1.15
*Strbp*	1.15	1.15
*Synm*	−1.12	1.15
*Map7d1*	1.14	1.16
*Specc1*	1.1	1.17
*Maea*	1.1	1.19
*Ncl*	1.1 and −1.1	1.19
*Glg1*	1.29	1.2
*Dnajc27*	1.17	1.21
*Ncdn*	1.14	1.21
*Lpcat2*	1.16	1.22
*D17Wsu92e*	1.11	1.24
*Nisch*	1.08	1.24
*Tkt*	−1.1	1.25
*Tmem59l*	1.29	1.26
*Mast3*	1.24	1.28
*Plac9a*	−1.45	1.35
*Nsg1*	−1.17	1.22 and 1.35
*Trappc3*	−1.2	1.32 and 1.41

Map1a, Ncl, Nsg1, and Trappc3 genes were represented by an additional probe.

### Verification of microarray results by qPCR

The results of qPCR verification for the six representative genes are shown in Table S3 (Supplementary material). The up and down regulations of the verified genes in the 40 days and 80 days old SOD1^G93A^ mice by means of qPCR were coincident and supported the microarray findings of correspondent animal ages (Table S3).

### FunNet analysis

**Figure 1:**
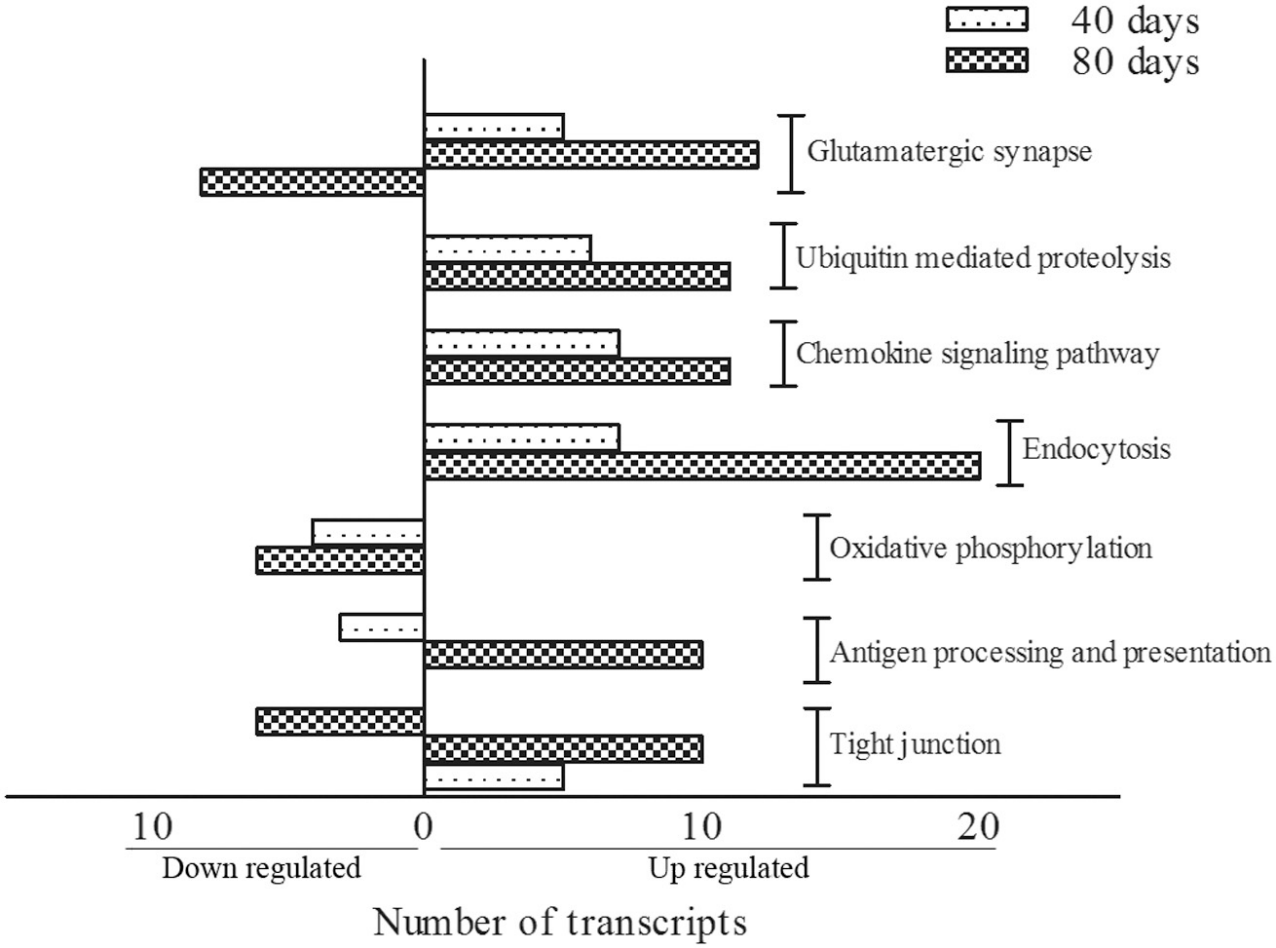
**KEGG pathways classification showing the number of transcripts up regulated and down regulated per category in 40 and 80 days pre-symptomatic SOD1^G93A^ mice in relation to age matched wild-types**. Bars on the left indicate the number of down regulated genes, and bars on the right indicate the number of up regulated genes, for each category.

KEGG terms which were significantly enriched (at level *p* < 0.05) amongst differentially expressed genes between SOD1^G93A^ and wild-type mice were identified for both 40 days and 80 days old pre-symptomatic ALS mice. Over-represented KEGG pathways and respective genes taking part of them are given in Tables 3, 4. Of importance, differentially expressed genes from 40 and 80 days old mice allowed to recognize 7 pathways common among both periods (Figure 1). Those were glutamatergic synapse, ubiquitin mediated proteolysis, chemokine signaling pathway, endocytosis, oxidative phosphorylation, antigen processing and presentation and tight junction. The number of transcripts in each pathway is also shown in Figure 1. Moreover, other interesting pathways could also be identified to appear only in 40 days (Table 3) or 80 days old SOD1^G93A^ mice (Table 4). Furthermore, among pathways common to both ages, ubiquitin mediated proteolysis, chemokine signaling pathway and endocytosis were over-represented by up regulated genes and oxidative phosphorylation was pointed by down regulated genes (Figure 1). Furthermore, glutamatergic synapse and tight junction were pointed by the genes that were up regulated in 40 days and also up or down regulated in 80 days gene expression lists (Figure 1). Finally, antigen processing and presentation was pointed for down regulated genes in 40 days and up regulated genes in 80 days lists (Figure 1).

**Table 3:** **KEGG pathways enriched amongst differentially expressed up or down regulated genes at 40 days old mice**.

**Gene ID**	**Gene symbol**	**Gene name**
**PATHWAYS POINTED BY UP REGULATED GENES**		
**Fructose and manose metabolism**		
170768	*Pfkfb3*	6-Phosphofructo-2-kinase/fructose-2,6-biphosphatase 3
18640	*Pfkfb2*	6-Phosphofructo-2-kinase/fructose-2,6-biphosphatase 2
18642	*Pfkm*	Phosphofructokinase, muscle
230163	*Aldob*	Aldolase B, fructose-bisphosphate
54384	*Mtmr7*	Myotubularin related protein 7
**Tight junction**		
14677	*Gnai1*	Guanine nucleotide binding protein (G protein), alpha inhibiting 1
16653	*Kras*	v-Ki-ras2 Kirsten rat sarcoma viral oncogene homolog
18176	*Nras*	Neuroblastoma ras oncogene
18417	*Cldn11*	Claudin 11
192195	*Ash1l*	Ash1 (absent, small, or homeotic)-like (Drosophila)
**Glutamatergic synapse**		
140919	*Slc17a6*	Solute carrier family 17 (sodium-dependent inorganic phosphate cotransporter), member 6
14677	*Gnai1*	Guanine nucleotide binding protein (G protein), alpha inhibiting 1
14802	*Gria4*	Glutamate receptor, ionotropic, AMPA4 (alpha 4)
14810	*Grin1*	Glutamate receptor, ionotropic, NMDA1 (zeta 1)
20511	*Slc1a2*	Solute carrier family 1 (glial high affinity glutamate transporter), member 2
**Axon guidance**		
12767	*Cxcr4*	Chemokine (C-X-C motif) receptor 4
14677	*Gnai1*	Guanine nucleotide binding protein (G protein), alpha inhibiting 1
16653	*Kras*	v-Ki-ras2 Kirsten rat sarcoma viral oncogene homolog
18176	*Nras*	Neuroblastoma ras oncogene
22253	*Unc5c*	Unc-5 homolog C (*C. elegans*)
56637	*Gsk3b*	Glycogen synthase kinase 3 beta
**Ubiquitin mediated proteolysis**		
107568	*Wwp1*	WW domain containing E3 ubiquitin protein ligase 1
17999	*Nedd4*	Neural precursor cell expressed, developmentally down-regulated 4
22210	*Ube2b*	Ubiquitin-conjugating enzyme E2B
59026	*Huwe1*	HECT, UBA and WWE domain containing 1
68729	*Trim37*	Tripartite motif-containing 37
70790	*Ubr5*	Ubiquitin protein ligase E3 component n-recognin 5
**Chemokine signaling pathway**		
12767	*Cxcr4*	Chemokine (C-X-C motif) receptor 4
14677	*Gnai1*	Guanine nucleotide binding protein (G protein), alpha inhibiting 1
16653	*Kras*	v-Ki-ras2 Kirsten rat sarcoma viral oncogene homolog
18176	*Nras*	Neuroblastoma ras oncogene
18708	*Pik3r1*	Phosphatidylinositol 3-kinase, regulatory subunit, polypeptide 1 (p85 alpha)
56637	*Gsk3b*	Glycogen synthase kinase 3 beta
73178	*Wasl*	Wiskott-Aldrich syndrome-like (human)
**Endocytosis**		
107568	*Wwp1*	WW domain containing E3 ubiquitin protein ligase 1
12767	*Cxcr4*	Chemokine (C-X-C motif) receptor 4
13854	*Epn1*	Epsin 1
17999	*Nedd4*	Neural precursor cell expressed, developmentally down-regulated 4
193740	*Hspa1a*	Heat shock protein 1A
26431	*Git2*	G protein-coupled receptor kinase-interactor 2
78618	*Acap2*	ArfGAP with coiled-coil, ankyrin repeat and PH domains 2
**PATHWAYS POINTED BY DOWN REGULATED GENES**		
**Nucleotide excision repair**		
19718	*Rfc2*	Replication factor C (activator 1) 2
66979	*Pole4*	Polymerase (DNA-directed), epsilon 4 (p12 subunit)
**DNA replication**		
19718	*Rfc2*	Replication factor C (activator 1) 2
66979	*Pole4*	Polymerase (DNA-directed), epsilon 4 (p12 subunit)
**Fatty acid metabolism**		
11363	*Acadl*	Acyl-Coenzyme A dehydrogenase, long-chain
74205	*Acsl3*	Acyl-CoA synthetase long-chain family member 3
**TGF-beta signaling pathway**		
12167	*Bmpr1b*	Bmpr1b bone morphogenetic protein receptor, type 1B
15902	*Id2*	Inhibitor of DNA binding 2
19651	*Rbl2*	Retinoblastoma-like 2
**Antigen processing and presentation**		
12010	*B2m*	Beta-2 microglobulin
12317	*Calr*	Calreticulin
19727	*Rfxank*	Regulatory factor X-associated ankyrin-containing protein
**ECM-receptor interaction**		
11603	*Agrn*	Agrin
12814	*Col11a1*	Collagen, type XI, alpha 1
16773	*Lama2*	Laminin, alpha 2
**GnRH signaling pathway**		
12314	*Calm2*	Calmodulin 2
16440	*Itpr3*	Inositol 1,4,5-triphosphate receptor 3
16476	*Jun*	Jun oncogene
17390	*Mmp2*	Matrix metallopeptidase 2
**Parkinson's disease**		
104130	*Ndufb11*	NADH dehydrogenase (ubiquinone) 1 beta subcomplex, 11
12857	*Cox4i1*	Cytochrome c oxidase subunit IV isoform 1
66576	*Uqcrh*	Ubiquinol-cytochrome c reductase hinge protein
67264	*Ndufb8*	NADH dehydrogenase (ubiquinone) 1 beta subcomplex 8
**Oxidative phosphorylation**		
104130	*Ndufb11*	NADH dehydrogenase (ubiquinone) 1 beta subcomplex, 11
12857	*Cox4i1*	Cytochrome c oxidase subunit IV isoform 1
66576	*Uqcrh*	Ubiquinol-cytochrome c reductase hinge protein
67264	*Ndufb8*	NADH dehydrogenase (ubiquinone) 1 beta subcomplex 8
**Alzheimer's disease**		
104130	*Ndufb11*	NADH dehydrogenase (ubiquinone) 1 beta subcomplex, 11
12314	*Calm2*	Calmodulin 2
12857	*Cox4i1*	Cytochrome c oxidase subunit IV isoform 1
16440	*Itpr3*	Inositol 1,4,5-triphosphate receptor 3
66576	*Uqcrh*	Ubiquinol-cytochrome c reductase hinge protein
67264	*Ndufb8*	NADH dehydrogenase (ubiquinone) 1 beta subcomplex 8

**Table 4:** **KEGG pathways enriched amongst differentially expressed up or down regulated genes at 80 days old mice**.

**Gene ID**	**Gene symbol**	**Gene name**
**PATHWAYS POINTED BY UP REGULATED GENES**		
**Vascular smooth muscle contraction**		
104111	*Adcy3*	Adenylate cyclase 3
12315	*Calm3*	Calmodulin 3
14673	*Gna12*	Guanine nucleotide binding protein, alpha 12
14674	*Gna13*	Guanine nucleotide binding protein, alpha 13
18751	*Prkcb*	Protein kinase C, beta
213498	*Arhgef11*	Rho guanine nucleotide exchange factor (GEF) 11
224129	*Adcy5*	Adenylate cyclase 5
26413	*Mapk1*	Mitogen-activated protein kinase 1
**Antigen processing and presentation**		
14963	*H2-Bl*	Histocompatibility 2, blastocyst
14972	*H2-K1*	Histocompatibility 2, K1, K region
15006	*H2-Q1*	Histocompatibility 2, Q region locus 1
15007	*H2-Q10*	Histocompatibility 2, Q region locus 10
15013	*H2-Q2*	Histocompatibility 2, Q region locus 2
15018	*H2-Q7*	Histocompatibility 2, Q region locus 7
15039	*H2-T22*	Histocompatibility 2, T region locus 22
15040	*H2-T23*	Histocompatibility 2, T region locus 23
15481	*Hspa8*	Heat shock protein 8
21355	*Tap2*	Transporter 2, ATP-binding cassette, sub-family B (MDR/TAP)
**Tight junction**		
11465	*Actg1*	Actin, gamma, cytoplasmic 1
13043	*Cttn*	Cortactin
13821	*Epb4.1l1*	Erythrocyte protein band 4.1-like 1
13822	*Epb4.1l2*	Erythrocyte protein band 4.1-like 2
14924	*Magi1*	Membrane associated guanylate kinase, WW and PDZ domain containing 1
16897	*Llgl1*	Lethal giant larvae homolog 1
17475	*Mpdz*	Multiple PDZ domain protein
18751	*Prkcb*	Protein kinase C, beta
67374	*Jam2*	Junction adhesion molecule 2
71960	*Myh14*	Myosin, heavy polypeptide 14
**Chemokine signaling pathway**		
104111	*Adcy3*	Adenylate cyclase 3
14083	*Ptk2*	PTK2 protein tyrosine kinase 2
14688	*Gnb1*	Guanine nucleotide binding protein (G protein), beta 1
14693	*Gnb2*	Guanine nucleotide binding protein (G protein), beta 2
14697	*Gnb5*	Guanine nucleotide binding protein (G protein), beta 5
14701	*Gng12*	Guanine nucleotide binding protein (G protein), gamma 12
14708	*Gng7*	Guanine nucleotide binding protein (G protein), gamma 7
18751	*Prkcb*	Protein kinase C, beta
224129	*Adcy5*	Adenylate cyclase 5
26413	*Mapk1*	Mitogen-activated protein kinase 1
277360	*Prex1*	Phosphatidylinositol-3,4,5-trisphosphate-dependent Rac exchange factor 1
**Ubiquitin mediated proteolysis**		
103583	*Fbxw11*	F-box and WD-40 domain protein 11
15204	*Herc2*	Hect (homologous to the E6-AP (UBE3A) carboxyl terminus) domain and RCC1 (CHC1)-like domain (RLD) 2
17237	*Mgrn1*	Mahogunin, ring finger 1
19823	*Rnf7*	Ring finger protein 7
217342	*Ube2o*	Ubiquitin-conjugating enzyme E2O
22192	*Ube2m*	Ubiquitin-conjugating enzyme E2M
22196	*Ube2i*	Ubiquitin-conjugating enzyme E2I
22213	*Ube2g2*	Ubiquitin-conjugating enzyme E2G 2
229615	*Pias3*	Protein inhibitor of activated STAT 3
50754	*Fbxw7*	F-box and WD-40 domain protein 7
63958	*Ube4b*	Ubiquitination factor E4B, UFD2 homolog (S. cerevisiae)
**Regulation of actin cytoskeleton**		
11465	*Actg1*	Actin, gamma, cytoplasmic 1
14083	*Ptk2*	PTK2 protein tyrosine kinase 2
14673	*Gna12*	Guanine nucleotide binding protein, alpha 12
14674	*Gna13*	Guanine nucleotide binding protein, alpha 13
14701	*Gng12*	Guanine nucleotide binding protein (G protein), gamma 12
18717	*Pip5k1c*	Phosphatidylinositol-4-phosphate 5-kinase, type 1 gamma
192897	*Itgb4*	Integrin beta 4
226970	*Arhgef4*	Rho guanine nucleotide exchange factor (GEF) 4
227753	*Gsn*	Gelsolin
26413	*Mapk1*	Mitogen-activated protein kinase 1
67771	*Arpc5*	Actin related protein 2/3 complex, subunit 5
71960	*Myh14*	Myosin, heavy polypeptide 14
**Glutamatergic synapse**		
104111	*Adcy3*	Adenylate cyclase 3
110637	*Grik4*	Glutamate receptor, ionotropic, kainate 4
14645	*Glul*	Glutamate-ammonia ligase (glutamine synthetase)
14688	*Gnb1*	Guanine nucleotide binding protein (G protein), beta 1
14693	*Gnb2*	Guanine nucleotide binding protein (G protein), beta 2
14697	*Gnb5*	Guanine nucleotide binding protein (G protein), beta 5
14701	*Gng12*	Guanine nucleotide binding protein (G protein), gamma 12
14708	*Gng7*	Guanine nucleotide binding protein (G protein), gamma 7
18751	*Prkcb*	Protein kinase C, beta
216456	*Gls2*	Glutaminase 2 (liver, mitochondrial)
224129	*Adcy5*	Adenylate cyclase 5
26413	*Mapk1*	Mitogen-activated protein kinase 1
**Phagosome**		
11465	*Actg1*	Actin, gamma, cytoplasmic 1
14963	*H2-Bl*	Histocompatibility 2, blastocyst
14972	*H2-K1*	Histocompatibility 2, K1, K region
15006	*H2-Q1*	Histocompatibility 2, Q region locus 1
15007	*H2-Q10*	Histocompatibility 2, Q region locus 10
15013	*H2-Q2*	Histocompatibility 2, Q region locus 2
15018	*H2-Q7*	Histocompatibility 2, Q region locus 7
15039	*H2-T22*	Histocompatibility 2, T region locus 22
15040	*H2-T23*	Histocompatibility 2, T region locus 23
15239	*Hgs*	HGF-regulated tyrosine kinase substrate
17113	*M6pr*	Mannose-6-phosphate receptor, cation dependent
21355	*Tap2*	Transporter 2, ATP-binding cassette, sub-family B (MDR/TAP)
22142	*Tuba1a*	Tubulin, alpha 1A
22151	*Tubb2a*	Tubulin, beta 2A class IIA
**Protein processing in endoplasmic reticulum**		
100037258	*Dnajc3*	DnaJ (Hsp40) homolog, subfamily C, member 3
108687	*Edem2*	ER degradation enhancer, mannosidase alpha-like 2
12955	*Cryab*	Crystallin, alpha B
15481	*Hspa8*	Heat shock protein 8
20014	*Rpn2*	Ribophorin II
20338	*Sel1l*	Sel-1 suppressor of lin-12-like (C. elegans)
216440	*Os9*	Amplified in osteosarcoma
22213	*Ube2g2*	Ubiquitin-conjugating enzyme E2G 2
269523	*Vcp*	Valosin containing protein
50907	*Preb*	Prolactin regulatory element binding
54197	*Rnf5*	Ring finger protein 5
56453	*Mbtps1*	Membrane-bound transcription factor peptidase, site 1
56812	*Dnajb2*	DnaJ (Hsp40) homolog, subfamily B, member 2
63958	*Ube4b*	Ubiquitination factor E4B, UFD2 homolog (S. cerevisiae)
**Cell adhesion molecules (CAMS)**		
14963	*H2-Bl*	Histocompatibility 2, blastocyst
14972	*H2-K1*	Histocompatibility 2, K1, K region
15006	*H2-Q1*	Histocompatibility 2, Q region locus 1
15007	*H2-Q10*	Histocompatibility 2, Q region locus 10
15013	*H2-Q2*	Histocompatibility 2, Q region locus 2
15018	*H2-Q7*	Histocompatibility 2, Q region locus 7
15039	*H2-T22*	Histocompatibility 2, T region locus 22
15040	*H2-T23*	Histocompatibility 2, T region locus 23
17967	*Ncam1*	Neural cell adhesion molecule 1
18007	*Neo1*	Neogenin
19274	*Ptprm*	Protein tyrosine phosphatase, receptor type, M
20340	*Glg1*	Golgi apparatus protein 1
20970	*Sdc3*	Syndecan 3
58235	*Pvrl1*	Poliovirus receptor-related 1
67374	*Jam2*	Junction adhesion molecule 2
**Endocytosis**		
11771	*Ap2a1*	Adaptor-related protein complex 2, alpha 1 subunit
12757	*Clta*	Clathrin, light polypeptide (Lca)
13196	*Asap1*	ArfGAP with SH3 domain, ankyrin repeat and PH domain1
13429	*Dnm1*	Dynamin 1
14963	*H2-Bl*	Histocompatibility 2, blastocyst
14972	*H2-K1*	Histocompatibility 2, K1, K region
15006	*H2-Q1*	Histocompatibility 2, Q region locus 1
15007	*H2-Q10*	Histocompatibility 2, Q region locus 10
15013	*H2-Q2*	Histocompatibility 2, Q region locus 2
15018	*H2-Q7*	Histocompatibility 2, Q region locus 7
15039	*H2-T22*	Histocompatibility 2, T region locus 22
15040	*H2-T23*	Histocompatibility 2, T region locus 23
15239	*Hgs*	HGF-regulated tyrosine kinase substrate
15481	*Hspa8*	Heat shock protein 8
16835	*Ldlr*	Low density lipoprotein receptor
18717	*Pip5k1c*	Phosphatidylinositol-4-phosphate 5-kinase, type 1 gamma
234852	*Chmp1a*	Charged multivesicular body protein 1A
243621	*Iqsec3*	IQ motif and Sec7 domain 3
67588	*Rnf41*	Ring finger protein 41
98366	*Smap1*	Stromal membrane-associated protein 1
**PATHWAYS POINTED BY DOWN REGULATED GENES**		
**VEGF signaling pathway**		
11651	*Akt1*	Thymoma viral proto-oncogene 1
16653	*Kras*	v-Ki-ras2 Kirsten rat sarcoma viral oncogene homolog
19056	*Ppp3cb*	Protein phosphatase 3, catalytic subunit, beta isoform
22339	*Vegfa*	Vascular endothelial growth factor A
**Long-term depression**		
14678	*Gnai2*	Guanine nucleotide binding protein (G protein), alpha inhibiting 2
14683	*Gnas*	GNAS (guanine nucleotide binding protein, alpha stimulating) complex locus
16653	*Kras*	v-Ki-ras2 Kirsten rat sarcoma viral oncogene homolog
18795	*Plcb1*	Phospholipase C, beta 1
60596	*Gucy1a3*	Guanylate cyclase 1, soluble, alpha 3
**Gap junction**		
14678	*Gnai2*	Guanine nucleotide binding protein (G protein), alpha inhibiting 2
14683	*Gnas*	GNAS (guanine nucleotide binding protein, alpha stimulating) complex locus
16653	*Kras*	v-Ki-ras2 Kirsten rat sarcoma viral oncogene homolog
18795	*Plcb1*	Phospholipase C, beta 1
60596	*Gucy1a3*	Guanylate cyclase 1, soluble, alpha 3
**RNA degradation**		
104625	*Cnot6*	CCR4-NOT transcription complex, subunit 6
13209	*Ddx6*	DEAD (Asp-Glu-Ala-Asp) box polypeptide 6
66373	*Lsm5*	LSM5 homolog, U6 small nuclear RNA associated (S. cerevisiae)
72662	*Dis3*	DIS3 mitotic control homolog (S. cerevisiae)
78651	*Lsm6*	LSM6 homolog, U6 small nuclear RNA associated (S. cerevisiae)
**Parkinson's disease**		
12866	*Cox7a2*	Cytochrome c oxidase subunit VIIa 2
333182	*Cox6b2*	Cytochrome c oxidase subunit VIb polypeptide 2
66142	*Cox7b*	Cytochrome c oxidase subunit VIIb
66495	*Ndufb3*	NADH dehydrogenase (ubiquinone) 1 beta subcomplex 3
66916	*Ndufb7*	NADH dehydrogenase (ubiquinone) 1 beta subcomplex, 7
68202	*Ndufa5*	NADH dehydrogenase (ubiquinone) 1 alpha subcomplex, 5
**Oxidative phosphorylation**		
12866	*Cox7a2*	Cytochrome c oxidase subunit VIIa 2
333182	*Cox6b2*	Cytochrome c oxidase subunit VIb polypeptide 2
66142	*Cox7b*	Cytochrome c oxidase subunit VIIb
66495	*Ndufb3*	NADH dehydrogenase (ubiquinone) 1 beta subcomplex 3
66916	*Ndufb7*	NADH dehydrogenase (ubiquinone) 1 beta subcomplex, 7
68202	*Ndufa5*	NADH dehydrogenase (ubiquinone) 1 alpha subcomplex, 5
**Tight junction**		
11651	*Akt1*	Thymoma viral proto-oncogene 1
14678	*Gnai2*	Guanine nucleotide binding protein (G protein), alpha inhibiting 2
16653	*Kras*	v-Ki-ras2 Kirsten rat sarcoma viral oncogene homolog
17888	*Myh6*	Myosin, heavy polypeptide 6, cardiac muscle, alpha
30960	*Vapa*	Vesicle-associated membrane protein, associated protein A
58187	*Cldn10*	Claudin 10
**Glutamatergic synapse**		
14678	*Gnai2*	Guanine nucleotide binding protein (G protein), alpha inhibiting 2
14683	*Gnas*	GNAS (guanine nucleotide binding protein, alpha stimulating) complex locus
14702	*Gng2*	Guanine nucleotide binding protein (G protein), gamma 2
14802	*Gria4*	Glutamate receptor, ionotropic, AMPA4 (alpha 4)
14805	*Grik1*	Glutamate receptor, ionotropic, kainate 1
18795	*Plcb1*	Phospholipase C, beta 1
19056	*Ppp3cb*	Protein phosphatase 3, catalytic subunit, beta isoform
216227	*Slc17a8*	Solute carrier family 17 (sodium-dependent inorganic phosphate cotransporter), member 8
**Huntington's disease**		
12866	*Cox7a2*	Cytochrome c oxidase subunit VIIa 2
18795	*Plcb1*	Phospholipase C, beta 1
333182	*Cox6b2*	Cytochrome c oxidase subunit VIb polypeptide 2
66142	*Cox7b*	Cytochrome c oxidase subunit VIIb
66495	*Ndufb3*	NADH dehydrogenase (ubiquinone) 1 beta subcomplex 3
66916	*Ndufb7*	NADH dehydrogenase (ubiquinone) 1 beta subcomplex, 7
68202	*Ndufa5*	NADH dehydrogenase (ubiquinone) 1 alpha subcomplex, 5
69920	*Polr2i*	Polymerase (RNA) II (DNA directed) polypeptide I
**Alzheimer's disease**		
11820	*App*	Amyloid beta (A4) precursor protein
12866	*Cox7a2*	Cytochrome c oxidase subunit VIIa 2
18795	*Plcb1*	Phospholipase C, beta 1
19056	*Ppp3cb*	Protein phosphatase 3, catalytic subunit, beta isoform
333182	*Cox6b2*	Cytochrome c oxidase subunit VIb polypeptide 2
66142	*Cox7b*	Cytochrome c oxidase subunit VIIb
66495	*Ndufb3*	NADH dehydrogenase (ubiquinone) 1 beta subcomplex 3
66916	*Ndufb7*	NADH dehydrogenase (ubiquinone) 1 beta subcomplex, 7
68202	*Ndufa5*	NADH dehydrogenase (ubiquinone) 1 alpha subcomplex, 5
**Ribosome**		
19951	*Rpl32*	Ribosomal protein L32
19981	*Rpl37a*	Ribosomal protein L37a
19982	*Rpl36a*	Ribosomal protein L36A
20068	*Rps17*	Ribosomal protein S17
20085	*Rps19*	Ribosomal protein S19
22186	*Uba52*	Ubiquitin A-52 residue ribosomal protein fusion product 1
57294	*Rps27*	Ribosomal protein S27
66489	*Rpl35*	Ribosomal protein L35
67945	*Rpl41*	Ribosomal protein L41
68028	*Rpl22l1*	Ribosomal protein L22 like 1
75617	*Rps25*	Ribosomal protein S25

Some pathways pointed by FunNet were omitted from table because they were composed by genes already presented in other pathways and also genes apparently not related to ALS. They were melanoma, measles, hepatitis C, melanogenesis, pathways in cancer and prostate cancer at 40 days and viral myocarditis and melanogenesis in 80 days results.

The results for GO enriched terms can be found in Tables S4 and S5 in Supplementary material.

### Laser microdissection of astrocytes and qPCR experiment

**Figure 2:**
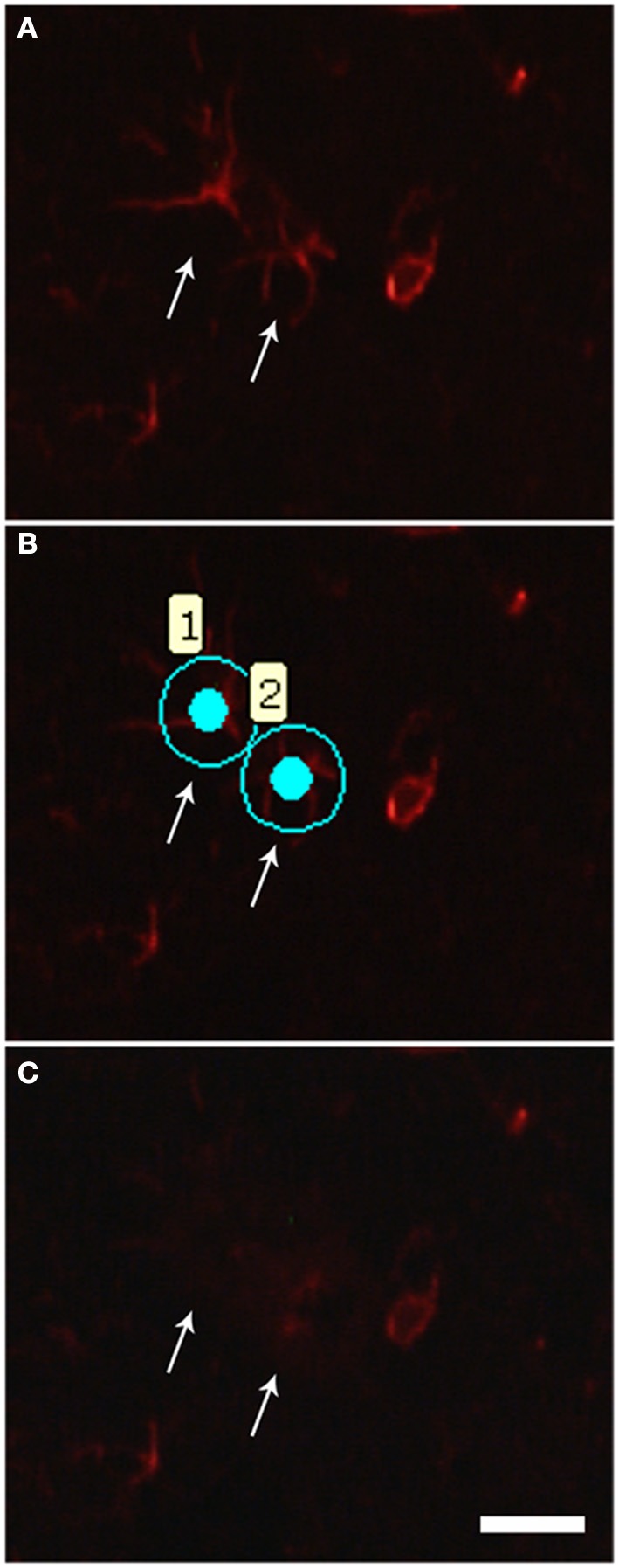
**Photomicrographs illustrating astrocyte laser microdissection process. (A)** The quick GFAP immunofluorescence allows recognizing the astrocytic profiles (arrows). **(B)** Astrocytes (1 and 2) were then selected for microdissection. **(C)** After laser firing and microdissection, selected cells (arrows) can no longer be visualized in the tissue. Scale bars of 20 μm.

**Figure 3:**
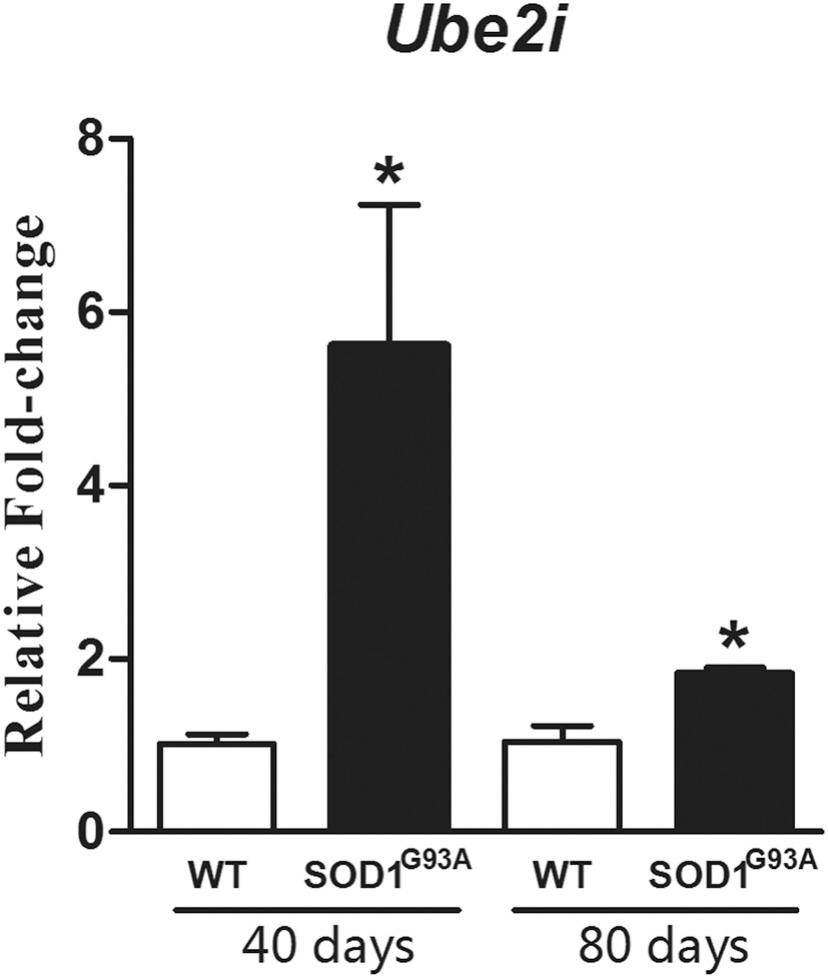
**Graph shows relative fold change values for *Ube2i* in microdissected astrocytes from 40 and 80 days old SOD1^G93A^ mice compared to the age matched wild-type controls (WT)**. Significant increases are seen in both transgenic astrocytes enriched samples. Results are presented as means ± s.e.m. from 3 samples used for each group. ^*^p-value < 0.05, according to unpaired *t*-test.

The profile for GFAP immunofluorescence for specific identification of astrocytes can be found in Figure 2. Our protocol allowed easily identifying the astrocytic profiles (Figure 2A) to be microdissected (Figure 2B). The procedure allowed a complete microdissection of the desired cell type (Figure 2C), the astrocytes in our case. The results of qPCR for *Ube2i*, using the two cycle amplified RNA, from 40 and 80 days mouse laser microdissected astrocytes have shown increased gene expressions in transgenic mice of both pre-symptomatic ages (Figure 3). The *Ube2i* expression was increased by 5.53-fold in the astrocytes from 40 days old SOD1^G92A^ mice and by 1.77-fold change in astrocytes from 80 days old SOD1^G92A^ mice compared to respective age matched wild-type samples.

## Discussion

Gene-expression profiling studies have been conducted in the search of molecular pathways related to motor neuron death in ALS by employing animal models in different phases of the disease and human post mortem material at the very end stage of motor neuron degeneration (Olsen et al., 2001; Dangond et al., 2004; Malaspina and De Belleroche, 2004; Jiang et al., 2005; Perrin et al., 2005; Ferraiuolo et al., 2007, 2011a; Yamamoto et al., 2007; Offen et al., 2009; Brockington et al., 2010; D'arrigo et al., 2010; Guipponi et al., 2010; Saris et al., 2013b).

The analysis of the mechanisms that trigger motor neuron death in the ALS may include evaluation of the altered molecular pathways that are taking place in compromised regions before the occurrence of cell death. Previous works have attempted to describe gene profiling in the pre-symptomatic phases of ALS animal model by employing distinct methodologies (Ferraiuolo et al., 2007; D'arrigo et al., 2010; Guipponi et al., 2010). This is the first work to analyze gene expression profile in the whole lumbar spinal cord of early 40 and 80 days old pre-symptomatic SOD1^G93A^ mouse in a whole genome array platform, which allowed depicting enriched pathways related to possible mechanisms of neuronal toxicity in ALS. Our analysis has pointed to up to 1105 differentially expressed genes in pre-symptomatic periods of SOD1^G93A^ mouse model, a larger number of than described elsewhere (Perrin et al., 2005, 2006). It should be pointed that the average of fold change described in previous publications is about 3, which is higher than that found in our microarray analysis. However, it must be emphasized that subtle changes in gene expression are exactly those that occur in initial stages of disease before the onset of clinical symptoms (Druyan et al., 2008). Moreover, some authors have argued that even small differences can be biologically relevant (Pedotti et al., 2008). Indeed, our qPCR verification analysis revealed higher fold changes than in the microarray, reaching values higher than 2 in the 80 days pre-symptomatic phase, which is closer to the symptom onset. The use of qPCR analysis to qualitatively verify the microarray results is largely accepted in the literature. However, it is well recognized that both methods have quantitative differences (Chuaqui et al., 2002), which are thought to be related to the variation in the hybridization kinetics of the technologies, low fold changes or lack of concordance between transcripts accessed in each method. The number of genes employed in qPCR validation is comparable to that found by other studies (Dallas et al., 2005; Brockington et al., 2010).

The differentially expressed genes with a *p*-value lower than 0.05 were submitted to enrichment analyses based on GO and KEGG databases, which correlated genes to already described related pathways and processes. Modulated genes based on GO evidenced more general biological processes that might be implicated in the ALS mechanisms. Of interest, regulation of astrocyte differentiation, protein retention in endoplasmatic reticulum lumen, Golgi vesicle transport and fructose metabolism, among others, were pointed at the pre-symptomatic 40 days old transgenic mice. At later pre-symptomatic phase of 80 days, the pattern of gene expression identified the GO terms post-Golgi vesicle-mediated transport, tricarboxylic acid cycle (TCA) and mRNA processing, among others. GO database analyses have been largely employed in the ALS research in several phases of the disease (Ferraiuolo et al., 2007, 2011a; Brockington et al., 2010).

Authors have also used the KEGG database to identify overrepresented pathways based on differentially expressed genes obtained by the microarray technique (Mougeot et al., 2011; Kalathur et al., 2012). The KEGG database analysis in the present work pointed to pathways that might be related to ALS mechanism at the pre-symptomatic ages of SOD1^G93A^ mice. Some pathways were found to be common to both pre-symptomatic periods, emphasizing the putative toxic triggering that may last before the onset of classical ALS symptoms with possible significance to mechanisms of initiation of motor neuron degeneration. Those pathways are going to be discussed below. It should be mentioned that alternative splicing have been recently implicated in ALS mechanisms (Lenzken et al., 2011; Singh and Cooper, 2012), however we could not access this biological event because the present analysis employed a platform designed to gene expression studies on 3′UTR that does not allow evaluation of alternative splicing variants.

### Glutamatergic synapse

The microarray profiling study by means of KEEG enriched analysis pointed to the category of glutamatergic synapse pathway in the lumbar spinal cord of ALS SOD1^G93A^. The large number of up regulated genes at 40 and 80 days underlines the excitotoxicity estate mediated by glutamatergic synapse of motor neurons in the pre-symptomatic condition of ALS disease (Bendotti et al., 2001; Gibb et al., 2007; Zhao et al., 2008; Jiang et al., 2009; Sunico et al., 2011). The modulation of GluR4, by means of *Gria4* findings in our microarray analysis, might reflect the dynamic state of the AMPA receptor subunit in the course of pre-symptomatic stages of ALS. At the early phases of the pre-symptomatic period, highly expressed *Gria4* gene might contribute to the AMPA receptor-mediated motor neuron toxicity, being a very early mechanism of the disease. The down regulation of the *Gria4* at the late pre-symptomatic stage could reflect a transient reactive mechanism to excitotoxic condition preceding motor neuron death. Reductions of GluR4 have been described at cellular level in the late disease stage of SOD1 mice, without alterations at the pre-symptomatic periods (Petri et al., 2005) thus, reflecting a disappearance of GluR4 containing neurons. In fact, imbalance of excitatory to inhibitory synaptic function precedes motor neuron degeneration as described in the spinal cord motor neurons in the late stage of pre-symptomatic phase of SOD1 ALS model by means of cellular analyses (Schutz, 2005). It should be mentioned that Ca^2+^ permeability of the AMPA receptor seems to occur mainly by the presence of the GluR2 subunit in the receptor complex. In fact, GluR2 deficiency clearly accelerated the motor neuron degeneration and shortened the life span of mutant SOD1^G93A^ double transgenic mice (Tateno et al., 2004). Synaptic GluR1 increases/mRNA up regulation, and decreases of synaptic and total GluR2 were found at early ages prior to disease onset thus prompting motor neurons to a higher Ca^2+^-permeable AMPA receptors -induced excitotoxicity (Zhao et al., 2008). The variant C-terminus of GluR4 (GluR4c), an alternative splicing isoform, stabilizes and locates AMPA receptors in the cell membrane, and also seems to potentate actions of GluR2 (Kawahara et al., 2004), thus highlighting the pivotal role of GluR4 subunit in regulating channel properties and trafficking of AMPA receptors. It must be then further clarified the role of GluR4 in the ALS mechanisms and possible dynamic interaction with that subunit with other AMPA receptor subtypes, especially GluR2.

The regulation of *Slc1a2* glial glutamate transporter (named EAAT2 or glial glutamate transporter GLT1) has not been evaluated in details. Excitotoxicity caused by a down-regulation of EAAT2 is thought to be a contributing factor to motor neuron death in ALS. Several mechanisms may account for impairment of EAAT2 function, for instance altered transcription/splicing, post-translational modifications, accelerated degradation, intracelular trafficking and inactivation by caspase-3 cleavage (Heath and Shaw, 2002; Boston-Howes et al., 2006) but not directly to gene regulation processes. It is possible that the impaired EAAT2 function could take place at the very early period of the pre-symptomatic stage, a matter that remains to be elucidated (Bendotti et al., 2001; Sasaki et al., 2001), thus, explaining the *Slc1a2* expression possibly related to motor neuron protection at those ages. The absence of this genomic process in the late pre-symptomatic period might potentiate loss of function of GLT1 thus culminating with the motor neuron death in ALS. Furthermore, the vesicular glutamate transporter 2 (VGLUT2), codified by *Slc17a6* gene, was found to be regulated and related to neuronal death in the pre-symptomatic stage of ALS model (Schutz, 2005; Sunico et al., 2011). The genetic reduction of VGLUT2 protein level in the ALS mouse model accounted for motor neuron rescue without modifying functional impairment (Wootz et al., 2010). It is possible that the up regulation of the *Slc17a6* gene at the early pre-symptomatic stage of the 40 days old SOD1^G93A^ mice potentiates the toxic state of motor neurons.

### Ubiquitin mediated proteolysis and oxidative phosphorylation

A recent meta-analysis study of the reported gene lists has described the evidences for a shared dysfunction in protein turnover in the ubiquitin-proteasome system in ALS mouse models and ALS patients (Saris et al., 2013a). Moreover, constitutive proteasome was decreased in motor neurons at the pre-symptomatic stage of SOD1^G93A^ (Cheroni et al., 2005), an alternative processes to decrease aggregate formation, thus an attempt to neuroprotect motor neurons of preclinical SOD1^G93A^ mice before the onset of clinical symptoms (Bendotti et al., 2012). That should be the case of *Nedd4* and *Fbxw7* expressions described here, whose encoded molecules have been already correlated to neuroprotection in ALS (Nateri et al., 2004; Matsumoto et al., 2011; Kwak et al., 2012). Moreover, it should be taken into attention the elevation of *Ubc12* in the spinal cord of SOD1^G93A^ mice at the pre-symptomatic phase (Massignan et al., 2007). *Ubc12* is an ubiquitin E2 ligase that adds NEDD-8 to substrates. *Ubc12* elevation in pre-symptomatic ALS was correlated to a tentative response to protein aggregation (Massignan et al., 2007). Interestingly, *Nedd8* gene was down regulated in our microarray analysis only in 80 days old mice, possibly representing a failure of the above described process close to the period of clinical onset.

The down regulation of genes over-representing the oxidative phosphorylation category at both pre-symptomatic ages of ALS mice seen in this work may be related to the progressive deteriorations of mitochondrial function and oxidative phosphorylation system described at pre-symptomatic ALS phases (Lin et al., 2009; Chen et al., 2010; Martin, 2010, 2011; Koopman et al., 2013), thus triggering reactive oxygen species (ROS) production (Manfredi and Xu, 2005) and motor neuron vulnerability before the onset of clinical symptoms. It is also interesting to notice that the TCA was seen as an over-represented GO term (Table S5, Supplementary material) in the up-regulated 80 days gene expression list. The TCA cycle is responsible to provide substrate to oxidative phosphorylation (Koopman et al., 2013) and its up regulation was previously seen in laser microdissected motor neurons from a VEGF model of ALS already in the pre-symptomatic period (Brockington et al., 2010). All in all, a possible mechanism of oxidative phosphorylation in the astrocyte-neuronal unit taking place in pre-symptomatic ALS might amplify motor neuron vulnerability to ROS damage.

### Chemokine signaling pathway and tight junction

The up regulation of all genes in the category chemokine signaling pathway in the pathogenesis of ALS is in agreement to previous publications (Henkel et al., 2006; Zhang et al., 2006; Rentzos et al., 2007; Kuhle et al., 2009; Sargsyan et al., 2009; Tateishi et al., 2010; Gupta et al., 2012). The up regulation of *Cxcr4* and *Pik3r1* described in this work is an important finding because the genes might be involved in non-autonomous toxicity in the early phase of ALS (Shideman et al., 2006; Luo et al., 2007; Manzano et al., 2011). Furthermore, disruption of blood-brain barrier and blood-spinal cord barrier are described as early events in ALS, thus impairing neurovascular unit prior motor neuron degeneration (Garbuzova-Davis et al., 2011, 2012; Grammas et al., 2011; Miyazaki et al., 2011). Indeed, reduced levels of adhesion molecules and the tight junction proteins zona occludens-1, occludin and claudin-5 are shown in post mortem tissue from patients and in ALS animal models (Zhong et al., 2008; Arhart, 2010; Garbuzova-Davis et al., 2012).

Our KEGG enriched analysis also demonstrated the modulation of tight junction related genes. Of substantial interest, we might point out the up regulation of *Cldn11* at 40 days and the down regulation of *Cldn10* at 80 days pre-symptomatic ALS mice, in agreement to previous description on differential regulation of tight junction genes related to specific characteristics of ALS clinical evolution (Henkel et al., 2009).

It is also important to highlight the particular modulation of the *Kras* gene, which has been up regulated at the age of 40 days and down regulated at the age of 80 days. The *Kras* gene is an oncogene that was located in the tight junction category by the KEGG analysis probably due its relation to topography of invading/proliferating cells in the scenario of neurodegenerative processes. Moreover, Kras proteins regulate cell activities such as proliferation, differentiation, apoptosis, and cell migration, those taking place in neurodegenerative processes-induced astroglial/microglial activation as well as expression of inflammatory and neurotrophic/neurotoxic mediators (Rotshenker, 2009). There is a marked proliferation/activation of both microglia and astrocytes at specific disease stages in ALS mouse models (Hall et al., 1998; Weydt et al., 2002) leading to the production of neuroprotective or pro-inflammatory molecules, which can decrease or increase the rate of primary motor neuron degeneration, respectively. Taken all together, up regulation of *Kras* gene at the early pre-symptomatic phase is in line with the early glial proliferative and reactivity events that will initiate the toxic triggering of non-autonomous cells and also the glial neuroprotective mechanisms to maintain temporarily the motor neurons. Later in that period, still before neuronal degeneration taking place, *Kras* gene down regulation might allow glial cells to drive toxic insult.

### Endocytosis and antigen processing and presentation

Endocytosis was an additional over-represented pathway in the pre-symptomatic stage of ALS. Genes found before clinical onset pointing to endocytosis have been related to clathrin-dependent/independent endocytosis, autophagy and also neurotransmission (Massey et al., 2006; Luo et al., 2007; Kon and Cuervo, 2010; McMahon and Boucrot, 2011; Elmer and McAllister, 2012), thus, related to extracellular turnover, repair of molecular processes and neuroprotection (Le Roy and Wrana, 2005; Doherty and McMahon, 2009; McMahon and Boucrot, 2011; Polymenidou and Cleveland, 2011). Disruption of these processes has been implicated as a general feature in the pathogenesis of ALS (Otomo et al., 2012), whereas there is a lack of information on that issue in pre-symptomatic periods (Morimoto et al., 2007; Tian et al., 2011). Clathrin-mediated endocytosis has a range of different physiological functions, remarkably the regulation of surface proteins, nutrition, activation of signaling pathways, protein trafficking and degradation of membrane components, in fact, mechanisms that might occur at the pre-symptomatic phases of ALS.

It is likely that the regulation of the genes for the heat shock proteins *Hspa1a* and *Hspa8* (also known as Hsp70-3 and Hsc70, respectively), described in our work is related to neuroprotective events before neurodegeneration, once treatment with recombinant human Hsp70 was able to both increase lifespan (Gifondorwa et al., 2007) and decrease neuromuscular junction denervation (Gifondorwa et al., 2012) in the SOD1^G93A^ mouse model. This protective role of Hsp70 has been also supported by other authors (Bruening et al., 1999; Takeuchi et al., 2002; Kieran et al., 2004). Actually, the increase of Hsc70 in the spinal cord of transgenic mice at pre-symptomatic ages of disease (Basso et al., 2009) and the demonstration of ubiquitinated Hsc70-induced degradation of mutant SOD1 (Urushitani et al., 2004) emphasized the possible neuroprotective role of heat shock protein regulation described in our work.

Furthermore, antigen processing and presentation pathway was also pointed as enriched among down regulated genes in 40 days old and up regulated genes in 80 days old pre-symptomatic SOD1^G93A^ mice. Genes presented in 40 and 80 days lists are mostly related to major histocompatibility complex (MHC) class I (*H2-Bl*, *H2-K1*, *H2-Q1*, *H2-Q10*, *H2-Q2*, *H2-Q7*, *H2-T22*, *H2-T23*—80 days ALS mice), molecules necessary for peptide loading (*Tap2*—80 days ALS mice) and to surface expression (*B2m*—40 days ALS mice) (Kimura and Griffin, 2000). *B2m* gene, possibly via cell surface MHC class I molecules, has been implicated in the synaptic plasticity at dendrites and axonal regeneration after peripheral nerve axotomy (Oliveira et al., 2004). It is possible that the down regulation of *B2m* in spinal cord from SOD1^G93A^ at pre-symptomatic ages is related to axonal and dendritic retractions and displacement of neuromuscular junction described as one of the earliest events faced by motor neurons in ALS models (Fischer et al., 2004). Our findings are in line with a description of down regulation of B2m protein reported in cerebrospinal fluid of ALS patients (Brettschneider et al., 2008), thus emphasizing the importance of its regulation in ALS. Additionally, *Rfxank* was down regulated at 40 days in our analysis, which is in agreement to a loss of MHC-II neuronal expression concurrent with abundant MHCII-positive microglia surrounding motor neurons in the pre-symptomatic SOD1^G93A^ mice (Casas et al., 2013), thus, interfering with the neuroimmunemodulation mediated by microglia (Graber et al., 2010; Sanagi et al., 2010). All in all, dysregulation of genes related to antigen processing and presentation might account for a number of intercellular mechanisms able to amplify the harmful non-autonomous cell toxicity at the pre-symptomatic stages of ALS.

### Laser microdissection of astrocytes

We performed laser microdissection of GFAP positive astrocytes from lumbar spinal cord ventral horn of SOD1^G93A^ transgenic and wild-type mice in the same pre-symptomatic ages of microarray analysis. The use of laser microdissection has been gained importance in recent years, once it allows specific cell enrichment from complex tissues, revealing to be a powerful tool in the study of neurodegenerative disorders in which individual cell types are known to be differentially involved in disease stages. The advantage of the methodology is the possibility to address molecular biology in the context of *in vivo* cellular analysis. The method is of substantial importance to evaluate changes in the astrocytes, the glial cell involved remarkably in toxic mechanisms of ALS. A previous study has employed laser microdissection of astrocytes to perform microarray experiments in ALS mouse model (Ferraiuolo et al., 2011a). The pattern of gene expression was first evaluated in the lumbar regions of the spinal cord in the present analysis, thus, taking into account all cell types from tissue. The depicted pathways represented the state of intercellular interaction in the pre-symptomatic studied periods of the ALS mouse model. The selected genes to be evaluated in type-specific cell, which is the case of *Ube2i* in the laser microdissected astrocytes described herein, would allow a closer analysis of astrocyte participation in the context of the neighbor cell toxicity. The *Ube2i* gene was then chosen for further evaluation in astrocytes by qPCR because astrocytes exert a non-autonomous cell toxicity to motor neurons and because SUMOylation pathway has gained importance in ALS mechanisms recently (for review, see Dangoumau et al., 2013). Increases of gene expression for *Ube2i* were found in enriched astrocytes samples from 40 and 80 days old pre-symptomatic mice, a regulation still not presented in the literature in that stage of disease, thus, entering in the context of ALS pathogenesis. In fact, conjugation of small ubiquitin-like modifier (SUMO) molecules involves a series of steps, being the ubiquitin conjugating enzyme E2, codified by *Ube2i* gene, responsible for the recognition of the target protein. SUMOylation is involved in the cellular response to oxidative stress, hypoxia, glutamate excitotoxicity and proteasome impairment, events that have been linked to motor neuron toxicity in ALS (Xu et al., 2011). Moreover, studies are required to determine the precise implication of the SUMO pathway in regulating the balance between cellular adaptive and neuroprotective response to stress (Fei et al., 2006; Dangoumau et al., 2013) with a special importance to motor neuron in the pre-symptomatic stage of ALS. Nevertheless, as discussed previously in this report, glutamate astroglial excitotoxicity faced by motor neurons in ALS is also hamfull by the cleavage of EAAT2 in the ventral horn of the spinal cord (Martin et al., 2007; Foran et al., 2011). The proteolytic fragments may be SUMOylated and accumulated in the nucleus of astrocytes (Boston-Howes et al., 2006; Foran et al., 2011) as described in SOD1^G93A^ mice, worsening the gliotoxic effects of astrocytes to motor neurons (Foran et al., 2011). Taking together, SUMOylation process and expression of *Ube2i* might participate in complex events related to the astrocyte-neuron unit in ALS, and future works are required to address specific cellular events.

In conclusion, the present work gives further evidence about molecular events taking place in the spinal cord from ALS mouse model before the onset of classical symptoms. The gene expression changes reflect responses for both neuroprotection and toxicity at the spinal cord in the evaluated periods. Indeed, the study of Ube2i expression in astrocytes adds novel insights for the participation of this cell type on the early mechanisms in ALS.

## Author contributions

Gabriela Pintar de Oliveira and Chrystian J. Alves performed the experiments. All authors designed the study, analyzed the results and wrote the manuscript. All authors read and approved the final manuscript.

### Conflict of interest statement

The authors declare that the research was conducted in the absence of any commercial or financial relationships that could be construed as a potential conflict of interest.
