# Supplementary material for: Early gene expression changes in spinal cord from SOD1G93A Amyotrophic Lateral Sclerosis animal model
Source: Front Cell Neurosci. 2013 Nov 18;7:216. doi: 10.3389/fncel.2013.00216 (PMC3831149; doi:10.3389/fncel.2013.00216)
Supplement: Supplementary file 1 [file DataSheet1.PDF]

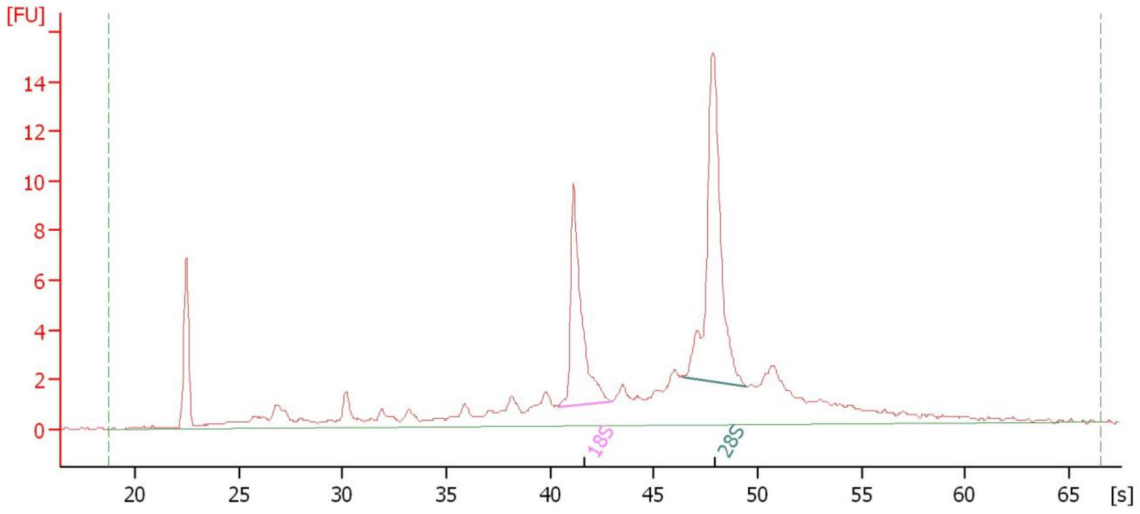

Figure S1. Eletropherogram representative of the results of RNA integrity obtained from samples employed in the experiments. The RNA integrity calculation is based on the ratios between 28S and 18S subunities from ribosomal RNA. The sample chosen for illustration presented a RNA integrity number (RIN) equal 8.4, once values of all samples ranged from 8 to 8.9.

Table S1. List containing differentially expressed genes (p<0.05) in 40 days old mice obtained from microarray analysis.

ProbeID	Gene Symbol	Absolute Fold Change	Log Fold Change	Average Expression	P.Value
A_51_P422030	Ocel1	-1.684203903	-0.752066813	7.693641523	0.0193
A_51_P471520	Stk25	-1.511899275	-0.596362028	9.484702689	0.0041
A_51_P468140	Serpind1	-1.50002447	-0.584986036	7.888358881	0.0086
A_51_P366672	Slc36a2	-1.495442306	-0.580572253	6.896484761	0.0434
A_51_P391668	D8Ertd738e	-1.480233806	-0.56582507	9.634933923	0.0358
A_52_P472233	Fcho1	-1.477685061	-0.563338821	8.468305393	0.0108
A_51_P104897	Itpr3	-1.465660969	-0.551551423	9.279208281	0.0310
A_52_P549977	Fam32a	-1.452688813	-0.53872569	8.441035643	0.0483
A_51_P215627	Plac9a	-1.451160261	-0.537206855	8.164177195	0.0258
A_52_P154101	Calca	-1.361205621	-0.444885014	10.50696082	0.0152
A_51_P505521	Hist1h4i	-1.340073761	-0.422312412	7.904763841	0.0123
A_51_P349495	Mboat1	-1.32114865	-0.401792801	7.997191901	0.0257
A_51_P237752	Ptrf	-1.317196395	-0.397470469	9.113590063	0.0401
A_52_P328492	Gas2l3	-1.314062023	-0.394033372	6.758856308	0.0411
A_52_P593268	Lsm6	-1.307745691	-0.387082016	10.03455332	0.0064
A_52_P655743	Lsm6	-1.303050917	-0.381893459	10.28659924	0.0116
A_51_P186703	Fbln5	-1.296806637	-0.37496338	7.802687575	0.0224
A_51_P239654	Nr4a1	-1.29562563	-0.373648913	8.529709806	0.0004
A_51_P501730	Crispld2	-1.282919321	-0.359430447	6.732376704	0.0030
A_51_P463552	Wdr78	-1.267688279	-0.342200034	7.930739463	0.0071
A_52_P652859	Lama2	-1.247474731	-0.319010593	6.54549276	0.0318
A_52_P472302	Fxyd6	-1.240253353	-0.310634858	10.51150932	0.0346
A_51_P354652	Slc25a30	-1.232895793	-0.302050866	6.665239626	0.0260

A_51_P106538	Htra3	-1.225962661	-0.29391504	7.691205265	0.0373
A_51_P204387	Tmem63c	-1.223706721	-0.291257837	7.505569353	0.0415
A_52_P240542	Id2	-1.21148868	-0.276780925	9.755582691	0.0399
A_51_P431852	Uqcrh	-1.21133136	-0.276593568	12.87071714	0.0026
A_51_P448479	Slc10a4	-1.210603032	-0.275725869	9.801982049	0.0313
A_51_P197850	Nr2c1	-1.208632133	-0.273375203	6.527456847	0.0504
A_51_P432930	Trappc3	-1.204846397	-0.268849233	7.922602777	0.0418
A_51_P291062	Col16a1	-1.200543621	-0.263687824	8.743457606	0.0351
A_52_P33202	Shisa3	-1.197557086	-0.260094428	6.549308425	0.0383
A_51_P149455	Acadl	-1.196028066	-0.258251244	7.676178571	0.0168
A_51_P246066	Slamf9	-1.195248863	-0.257311033	6.783212915	0.0065
A_51_P440923	Sh3pxd2a	-1.19279686	-0.254348365	8.705582483	0.0385
A_51_P173961	Pdrg1	-1.187068491	-0.247403178	10.22646006	0.0414
A_52_P560728	Serhl	-1.185881532	-0.245959893	7.81046069	0.0504
A_51_P295286	1700066M21Rik	-1.182519279	-0.241863705	6.614938881	0.0503
A_51_P482571	Wnt6	-1.181702454	-0.240866819	6.607549263	0.0046
A_51_P511270	Pou3f1	-1.181446461	-0.240554253	9.640979356	0.0499
A_51_P356760	Mical1	-1.180314804	-0.239171694	8.856263311	0.0409
A_51_P129012	B2m	-1.17990474	-0.238670388	8.921867312	0.0074
A_51_P444264	Rtn1	-1.17846279	-0.236906206	12.73174614	0.0025
A_51_P406157	Calcb	-1.176665896	-0.234704738	7.647561606	0.0252
A_51_P151862	Lims2	-1.176656605	-0.234693346	9.418441063	0.0276
A_51_P191865	Lama2	-1.175510493	-0.233287417	6.740090352	0.0392
A_52_P318532	Tbx2	-1.173648332	-0.231000189	6.895468919	0.0467
A_52_P467726	Nsg1	-1.169555268	-0.225960038	11.83298885	0.0417
A_52_P92161	Drp2	-1.168617189	-0.224802415	6.700269822	0.0278
A_51_P185971	Calm2	-1.165349773	-0.220763037	12.79660428	0.0027
A_51_P516125	NA	-1.163706878	-0.218727709	9.314587305	0.0394
A_51_P230439	Ppfibp2	-1.1588511	-0.212695207	9.113521449	0.0274
A_51_P316103	Lima1	-1.157310414	-0.210775877	8.190774021	0.0324
A_52_P640413	Igflr1	-1.155944446	-0.209072064	7.231814262	0.0242
A_52_P117325	Rbl2	-1.155494332	-0.208510183	7.060601177	0.0423
A_52_P475170	4931432P07Rik	-1.154634782	-0.207436591	6.509332538	0.0276
A_51_P181922	Yjefn3	-1.154599579	-0.207392605	7.456058711	0.0167
A_51_P205573	Ndufb11	-1.154046257	-0.206701052	11.95395442	0.0466
A_51_P517982	Gabarapl2	-1.153578907	-0.206116691	10.59975993	0.0329
A_52_P163640	Ccdc126	-1.151889618	-0.204002474	6.730158785	0.0219
A_52_P981680	Rfc2	-1.151798012	-0.203887737	9.677069998	0.0021
A_51_P416419	Calr	-1.150902263	-0.202765322	9.576280091	0.0450
A_52_P590665	Tmem161a	-1.150855496	-0.202706696	8.066935632	0.0333
A_51_P225224	Htra1	-1.150174679	-0.201852983	12.92334571	0.0342
A_52_P677718	Tatdn2	-1.149841808	-0.201435393	6.774786236	0.0324
A_51_P199725	Arhgap24	-1.149194993	-0.200623612	8.248315797	0.0109
A_52_P241519	Myo1c	-1.148719018	-0.200025951	6.934629803	0.0414
A_52_P563617	Ssbp4	-1.148201644	-0.199376027	8.655105275	0.0405

A_52_P541826	Eif4a1	-1.148021609	-0.199149798	7.366202031	0.0351
A_52_P586944	Bmpr1b	-1.14723153	-0.198156581	6.709881296	0.0328
A_51_P362429	Myh11	-1.14648192	-0.197213604	6.791138141	0.0022
A_52_P131254	NA	-1.143654516	-0.193651297	12.03548716	0.0137
A_51_P346715	D4Wsu53e	-1.142552941	-0.192261015	11.76184176	0.0386
A_51_P414653	Plvap	-1.1400321	-0.189074447	7.388246355	0.0117
A_52_P56751	Lcp1	-1.1400032	-0.189037874	7.721925252	0.0258
A_51_P483180	Snx7	-1.138793936	-0.187506716	7.448905482	0.0435
A_52_P222350	NA	-1.136847658	-0.18503894	7.149428133	0.0039
A_52_P577019	Rps15a	-1.136110947	-0.184103728	10.60917565	0.0483
A_51_P397934	Grin3b	-1.134676342	-0.182280839	8.682239412	0.0225
A_52_P335478	Pole4	-1.134280479	-0.181777426	8.353686974	0.0286
A_52_P562807	3110052M02Rik	-1.134206208	-0.181682958	6.972088679	0.0089
A_52_P362161	Rab3b	-1.133581449	-0.180888054	7.884499914	0.0218
A_51_P292276	Agrn	-1.133564319	-0.180866253	10.96310735	0.0164
A_52_P694988	Zfp933	-1.132539112	-0.179560874	6.445424832	0.0429
A_51_P100063	Lnx1	-1.129828851	-0.176104247	8.056583053	0.0458
A_51_P141136	Tnrc6a	-1.129721412	-0.17596705	7.900249839	0.0503
A_51_P153124	Emcn	-1.129320362	-0.175454803	7.924206905	0.0360
A_51_P459477	Col11a1	-1.1287304	-0.174700935	7.036338775	0.0482
A_51_P394515	Tkt	-1.128632436	-0.174575718	11.97122811	0.0157
A_51_P115178	Scara3	-1.127709761	-0.173395809	6.564525693	0.0296
A_52_P322962	NA	-1.126929489	-0.17239725	6.61055956	0.0219
A_52_P96360	NA	-1.126859871	-0.172308122	8.156790445	0.0482
A_51_P507778	Sdr42e1	-1.126669688	-0.172064614	8.499648072	0.0069
A_52_P484838	Rfxank	-1.126455932	-0.171790875	7.365826798	0.0307
A_51_P319551	Kif3a	-1.12640871	-0.171730395	7.884179598	0.0096
A_52_P272364	Gria3	-1.125954659	-0.171148733	6.452039429	0.0199
A_52_P480141	Plxna1	-1.12547476	-0.170533703	6.971589255	0.0346
A_51_P436878	Sertad1	-1.124149093	-0.16883339	7.508588517	0.0209
A_51_P404875	Synm	-1.122891839	-0.167218969	9.913507348	0.0441
A_52_P473966	Kdelr3	-1.122875907	-0.167198499	6.611004032	0.0236
A_51_P341736	Mmp2	-1.120313494	-0.163902494	6.970641561	0.0242
A_51_P165435	Cox4i1	-1.119811506	-0.163255908	12.99347746	0.0417
A_51_P451574	Acot1	-1.119807333	-0.163250533	8.709608198	0.0243
A_52_P438359	Dnajc19	-1.119571163	-0.162946233	7.079379491	0.0464
A_52_P134075	Osbpl5	-1.116635563	-0.15915841	9.216698262	0.0142
A_51_P233059	Ktn1	-1.116222594	-0.158624753	11.22966162	0.0138
A_52_P218590	NA	-1.115770076	-0.158039765	6.457407466	0.0237
A_52_P623337	Ncl	-1.114788521	-0.156770052	11.60649659	0.0172
A_52_P49601	Fth1	-1.114326981	-0.15617263	14.86527232	0.0121
A_52_P279579	Nyap1	-1.114279795	-0.156111538	9.467362003	0.0400
A_52_P453650	NA	-1.11271514	-0.154084304	9.3743494	0.0346
A_51_P277345	Ostf1	-1.112175795	-0.153384844	8.418080013	0.0397
A_52_P198898	Samd5	-1.111861678	-0.15297732	6.769812595	0.0195

A_52_P179599	NA	-1.111715053	-0.152787054	13.07017311	0.0230
A_51_P331021	Ttc32	-1.110571279	-0.151301992	7.249097334	0.0441
A_51_P234113	Nod1	-1.108684025	-0.148848256	8.538987806	0.0376
A_52_P400999	Arhgap31	-1.108404774	-0.14848483	7.510853157	0.0486
A_51_P437426	Lrrc33	-1.107047899	-0.146717645	7.339477658	0.0402
A_51_P153423	NA	-1.105753195	-0.145029411	7.050478404	0.0415
A_52_P515347	Tusc3	-1.104896246	-0.143910902	8.596401451	0.0480
A_51_P247873	Ndufb8	-1.103122379	-0.14159285	12.39090077	0.0151
A_51_P290921	Sytl2	-1.10308751	-0.141547247	8.024316605	0.0292
A_51_P487105	Bud31	-1.103059773	-0.141510971	7.704943805	0.0237
A_52_P685971	Srsf11	-1.101814325	-0.139881125	9.25257059	0.0340
A_52_P324566	Midn	-1.101162374	-0.13902722	8.119583408	0.0321
A_52_P405193	Prkrir	-1.100571066	-0.138252305	6.891052808	0.0188
A_51_P386304	Ccnl2	-1.097999153	-0.134876941	8.534202552	0.0363
A_51_P273843	Spcs2	-1.09726909	-0.13391737	9.995090394	0.0432
A_52_P42380	Tmem106c	-1.097016012	-0.133584584	7.113249978	0.0191
A_51_P293938	Rasl11b	-1.095890876	-0.132104148	8.01933226	0.0250
A_52_P555089	NA	-1.095885221	-0.132096704	9.242270375	0.0430
A_52_P305307	Sh3bp5	-1.094988578	-0.13091582	8.918811607	0.0148
A_52_P424585	Ctnnb1	-1.094700947	-0.130536804	9.827308238	0.0250
A_52_P654604	NA	-1.093764322	-0.129301908	6.938714739	0.0312
A_51_P279851	Dhps	-1.092760706	-0.127977513	8.346201884	0.0156
A_51_P420731	Thy1	-1.088498518	-0.122339443	7.554144872	0.0268
A_52_P53596	Sesn1	-1.088199405	-0.121942945	6.978765026	0.0333
A_51_P325914	Jun	-1.086946108	-0.120280411	7.77928671	0.0428
A_51_P441837	Tmem53	-1.086816145	-0.120107902	7.758364613	0.0496
A_52_P218058	Clec5a	-1.085135891	-0.117875721	6.709141334	0.0284
A_51_P290986	Dhcr7	-1.085029493	-0.117734258	9.819274469	0.0489
A_51_P520936	Bcar3	-1.084056	-0.116439286	7.52614621	0.0419
A_51_P447595	Scube1	-1.083079713	-0.115139427	7.126570672	0.0439
A_52_P112721	Commd8	-1.080863208	-0.112183949	7.741206098	0.0238
A_52_P52964	Hist1h4f	-1.080506219	-0.111707376	9.335413596	0.0442
A_51_P123077	Nubp1	-1.080217197	-0.111321421	7.726310934	0.0485
A_51_P450123	Mrpl36	-1.079043887	-0.109753543	9.061627839	0.0413
A_52_P61691	Cd59b	-1.07739746	-0.107550569	6.438355205	0.0401
A_52_P400355	3110035E14Rik	-1.076535933	-0.106396475	8.581471851	0.0391
A_51_P359333	Fh1	-1.074925128	-0.104236176	10.1440364	0.0451
A_52_P661731	2-Mar	-1.073201431	-0.101920884	9.317653666	0.0397
A_51_P511560	Acsl3	-1.072372389	-0.100805979	10.20132422	0.0345
A_51_P310548	Osgep	-1.070125307	-0.09777974	9.404860589	0.0289
A_51_P465600	Usp48	-1.069989955	-0.097597253	8.09082632	0.0356
A_51_P115159	Fam162a	-1.06785814	-0.094720004	10.04945669	0.0503
A_51_P306160	Map3k13	1.06000923	0.084076826	6.459207426	0.0482
A_52_P342836	NA	1.063238115	0.088464729	7.67427738	0.0503
A_52_P477286	Rab8b	1.063783487	0.089204547	6.480474084	0.0485

A_51_P428781	Pbx4	1.066316259	0.092635391	6.441723827	0.0465
A_51_P451176	Bhlhe41	1.067763349	0.094591934	6.436438696	0.0386
A_52_P354286	Dab1	1.068095347	0.095040439	6.79100819	0.0457
A_52_P971290	NA	1.069426509	0.096837344	6.388147176	0.0414
A_52_P308681	Atxn3	1.069625291	0.097105484	7.143599768	0.0500
A_52_P142912	Pfkfb2	1.069921059	0.097504356	6.563841053	0.0386
A_51_P237548	Dzank1	1.069931708	0.097518715	6.552715259	0.0456
A_52_P400509	Atm	1.070560861	0.098366815	6.648937292	0.0480
A_51_P399653	Crhr2	1.071257523	0.099305335	6.474436128	0.0371
A_52_P475886	Rc3h1	1.07129541	0.099356359	6.462070776	0.0404
A_52_P169181	Auts2	1.071525568	0.099666275	6.448417719	0.0486
A_52_P796682	Ccne1	1.072077481	0.100409175	6.507993066	0.0358
A_52_P156932	Wac	1.072169989	0.100533659	6.594795584	0.0499
A_52_P138126	Pfkfb3	1.072316732	0.1007311	6.415542702	0.0347
A_51_P437847	Kctd1	1.072460377	0.100924347	8.346472068	0.0353
A_52_P213004	Pacs2	1.073560622	0.102403659	6.833703418	0.0487
A_52_P201482	Prickle2	1.073812473	0.102742069	6.501700788	0.0287
A_52_P585124	Cxcr4	1.073857874	0.102803065	6.402537647	0.0488
A_52_P201972	Zfp148	1.073884167	0.102838387	6.641158699	0.0485
A_52_P710826	NA	1.074069357	0.103087157	6.590939122	0.0503
A_52_P323074	Epn1	1.074148036	0.103192835	6.516347779	0.0279
A_51_P351948	NA	1.074288187	0.10338106	7.317065768	0.0476
A_52_P423859	Nvl	1.074648261	0.103864535	6.537103582	0.0296
A_52_P367760	Calml4	1.074668881	0.103892216	6.615731351	0.0460
A_52_P336080	Eif5	1.074684921	0.103913748	6.57280199	0.0501
A_52_P69292	Grin1	1.075566303	0.105096461	6.969950477	0.0412
A_51_P260008	NA	1.076006592	0.105686916	6.682705801	0.0439
A_52_P22781	Zfp866	1.076512564	0.106365158	6.907296691	0.0431
A_51_P363525	Fbrsl1	1.076770864	0.106711279	8.258417296	0.0252
A_52_P500077	Zfp551	1.077496328	0.107682953	7.288904681	0.0397
A_51_P185141	Myo1e	1.077531668	0.107730269	6.742450971	0.0378
A_51_P164895	Slc25a36	1.077626994	0.107857895	6.718675553	0.0314
A_52_P581390	Kif1c	1.077892322	0.108213064	6.60746979	0.0276
A_52_P16209	2610507B11Rik	1.0779773	0.108326798	6.523147977	0.0465
A_52_P155302	Ankib1	1.078182678	0.108601637	6.599670552	0.0447
A_52_P675039	Fhad1	1.078580346	0.10913365	6.548780338	0.0225
A_52_P250278	Dhx29	1.078871379	0.10952288	7.065128526	0.0463
A_51_P511612	NA	1.078895089	0.109554585	6.497744878	0.0443
A_52_P214630	Sox9	1.079332129	0.110138875	7.56642132	0.0361
A_51_P320650	C77370	1.079821367	0.110792669	6.623724702	0.0323
A_52_P459657	Pcsk1n	1.079969242	0.110990225	6.692500328	0.0504
A_51_P352005	Hsd3b4	1.080084326	0.111143954	6.502004016	0.0210
A_51_P285779	Asphd2	1.080109026	0.111176945	9.679522967	0.0445
A_52_P496497	Abhd6	1.080174702	0.111264665	7.753295415	0.0427
A_51_P437608	Tulp3	1.080210077	0.111311912	6.757037836	0.0336

A_51_P459741	Gprasp1	1.080555773	0.111773539	6.802390062	0.0449
A_51_P143103	Pprc1	1.0806461	0.111894134	7.309051064	0.0297
A_52_P303491	Grid2	1.080726417	0.112001355	7.408870504	0.0463
A_52_P168549	Fgf14	1.080767317	0.112055952	6.503691572	0.0403
A_52_P297212	Zkscan3	1.081472863	0.112997465	7.171825888	0.0301
A_52_P550884	Samd12	1.081685368	0.113280921	6.505505258	0.0365
A_52_P380301	Unc5c	1.08174632	0.113362213	7.103945431	0.0477
A_52_P145033	Nisch	1.081915037	0.113587209	6.897340535	0.0345
A_52_P395149	Smtnl2	1.082395231	0.114227387	7.187672298	0.0377
A_52_P561236	Bri3bp	1.082463478	0.114318349	6.932265918	0.0306
A_51_P244154	Lrrc8b	1.082545511	0.114427678	7.238964025	0.0430
A_51_P346893	Extl1	1.082625314	0.114534027	6.685628725	0.0291
A_51_P233267	NA	1.082674133	0.114599082	6.825737504	0.0386
A_52_P947423	NA	1.082993731	0.115024892	6.443921427	0.0174
A_51_P275591	Zfp292	1.083233102	0.115343732	7.05019541	0.0421
A_51_P178575	Brd3	1.083762708	0.11604891	8.930068417	0.0368
A_52_P649296	Nras	1.083837783	0.116148847	6.719202515	0.0248
A_51_P294156	4930422G04Rik	1.083875629	0.116199222	6.6325791	0.0177
A_52_P679711	4930538K18Rik	1.084132684	0.116541336	6.395477475	0.0384
A_51_P437050	Heg1	1.084242532	0.116687507	6.536603336	0.0472
A_51_P332359	Med6	1.084253195	0.116701695	7.155800767	0.0281
A_51_P205820	Klf11	1.084488252	0.117014425	6.469622806	0.0285
A_51_P476900	NA	1.084516882	0.11705251	7.529614971	0.0284
A_51_P156222	Elfn1	1.084828372	0.117466815	7.722203308	0.0277
A_51_P343350	Amn	1.084980987	0.117669762	7.097276805	0.0126
A_52_P738798	NA	1.085272989	0.118057983	6.476066349	0.0274
A_52_P533724	Ino80	1.087465512	0.120969648	6.522569488	0.0249
A_52_P118638	Senp5	1.087738393	0.121331623	6.857888853	0.0347
A_51_P416243	Exosc9	1.087761545	0.121362329	7.571865451	0.0423
A_52_P51564	Arhgap10	1.087892308	0.121535749	6.561122387	0.0235
A_51_P393748	Ddx58	1.087896163	0.121540862	6.502722954	0.0342
A_51_P275915	Ubr5	1.088160967	0.121891984	6.723347244	0.0434
A_52_P539414	Gtf2h3	1.088197365	0.12194024	7.200936622	0.0466
A_52_P612079	Prepl	1.088308111	0.122087056	8.418121513	0.0503
A_51_P152797	2810039B14Rik	1.088416534	0.122230778	6.588098557	0.0430
A_52_P198289	NA	1.088717408	0.122629531	6.592297673	0.0146
A_51_P417758	Fut9	1.088834558	0.122784762	6.403301375	0.0289
A_52_P582384	Narf	1.089006739	0.123012882	6.720126668	0.0307
A_52_P527977	Sdk2	1.089072146	0.123099529	7.03488123	0.0199
A_52_P457028	Mia3	1.089142496	0.123192719	6.417400325	0.0333
A_51_P408881	Pdlim5	1.089836895	0.124112237	6.638607246	0.0503
A_52_P459399	Tnrc6b	1.089889086	0.124181325	6.780039284	0.0334
A_51_P264956	Kif1b	1.089953713	0.124266869	6.545731536	0.0243
A_52_P574214	Rrp1b	1.090082967	0.124437943	6.570424443	0.0215
A_52_P627306	Mtf2	1.091288676	0.126032785	7.707880463	0.0270

A_51_P507851	Gcc1	1.091420049	0.126206451	7.099094354	0.0341
A_52_P24696	Mgat5	1.091517488	0.126335245	6.439985475	0.0138
A_52_P111145	NA	1.091756086	0.126650573	6.55947474	0.0503
A_52_P596360	NA	1.091870408	0.126801636	6.454141665	0.0273
A_52_P266106	Usp38	1.091924168	0.126872667	6.57602032	0.0244
A_51_P311319	Scrn3	1.092771147	0.127991298	6.735616419	0.0434
A_51_P135654	Eif4ebp2	1.093115955	0.128446446	7.22438544	0.0363
A_52_P379631	Steap2	1.093228073	0.128594413	6.643570391	0.0184
A_52_P333749	Rmdn3	1.093493464	0.128944597	6.757710651	0.0230
A_51_P381440	Zfp40	1.093516964	0.128975602	6.621030526	0.0334
A_52_P411358	NA	1.093822534	0.129378689	6.534411904	0.0349
A_51_P101621	Creb1	1.093834256	0.129394149	7.243676625	0.0502
A_51_P273044	Baz2a	1.094006336	0.129621093	8.333217638	0.0243
A_51_P459873	6330411E07Rik	1.094073717	0.129709947	6.844400572	0.0301
A_52_P5549	Fam133b	1.094180114	0.129850241	7.427069026	0.0470
A_51_P459091	Ybey	1.094293234	0.129999384	6.630118927	0.0149
A_51_P520412	Rabl6	1.094552884	0.130341661	8.271860075	0.0483
A_51_P170987	Rgs7bp	1.095375246	0.131425182	7.710453526	0.0272
A_52_P545255	Cpsf2	1.095454323	0.13152933	7.043433663	0.0313
A_52_P462366	NA	1.095787151	0.131967592	6.723106913	0.0120
A_51_P377045	Malat1	1.095944378	0.13217458	7.214187153	0.0487
A_52_P662600	Pdlim5	1.096467955	0.132863648	6.601390607	0.0222
A_51_P355589	Fjx1	1.096966255	0.133519146	7.253413145	0.0293
A_52_P539434	Lbh	1.097006976	0.1335727	6.551972129	0.0225
A_52_P287692	Stk32c	1.097803781	0.134620213	6.593477472	0.0233
A_51_P456466	Mlxipl	1.098291296	0.135260746	6.681335625	0.0206
A_52_P367294	Fsd1l	1.098859243	0.136006597	6.695014527	0.0175
A_51_P278843	NA	1.099032127	0.13623356	6.917853509	0.0441
A_52_P348189	Krtcap3	1.099213793	0.136472013	6.455975989	0.0279
A_52_P540855	Prdx6	1.099271268	0.136547445	8.07508804	0.0482
A_52_P318683	NA	1.099692685	0.137100411	6.673574769	0.0277
A_52_P22180	Usp38	1.099730448	0.137149952	6.515267626	0.0266
A_52_P218976	Cyld	1.100068973	0.137593982	6.769154602	0.0264
A_52_P497553	Dhfr	1.100416526	0.138049711	6.830856421	0.0228
A_52_P74368	Slc43a2	1.100564735	0.138244006	7.193275614	0.0466
A_52_P617817	Hspa4	1.100946297	0.138744098	7.006740376	0.0241
A_51_P141290	Plch2	1.101481937	0.139445837	7.186340428	0.0323
A_52_P416123	Malat1	1.101677499	0.139701956	7.75307016	0.0380
A_52_P142965	Brd4	1.101700502	0.13973208	8.411761079	0.0385
A_52_P287338	Eif4e3	1.10175757	0.139806809	7.077579204	0.0389
A_51_P242043	Dhcr24	1.102355041	0.140588955	9.607037152	0.0192
A_51_P487360	Hpcal1	1.102610913	0.140923785	9.123670934	0.0316
A_51_P436534	Twf1	1.103549951	0.142151934	6.581332991	0.0421
A_51_P266964	Slc35d1	1.103703861	0.142353128	6.783821099	0.0300
A_51_P378381	4933436C20Rik	1.104342271	0.143187378	6.540995642	0.0285

A_52_P307938	Pik3r1	1.10457173	0.143487109	6.867673555	0.0130
A_52_P274238	Maea	1.104589372	0.143510152	9.216517126	0.0423
A_51_P153982	Specc1	1.10496513	0.144000842	10.36876278	0.0412
A_52_P641849	Khnyn	1.104976735	0.144015994	6.646357294	0.0307
A_52_P429650	Ncl	1.105305089	0.144444641	7.762715417	0.0501
A_52_P106251	Git2	1.105704757	0.144966212	6.533630492	0.0350
A_52_P494230	Brd4	1.106035124	0.145397202	7.652129206	0.0442
A_52_P381430	NA	1.106212339	0.14562834	6.774465425	0.0167
A_51_P418859	Zfp599	1.106683916	0.146243228	6.857594508	0.0401
A_51_P396570	Plod2	1.106784874	0.146374833	6.980406425	0.0066
A_51_P263004	Bcl11a	1.106820882	0.146421768	7.348113464	0.0380
A_52_P54297	Rbm27	1.107246655	0.146976638	6.718332815	0.0079
A_52_P593361	Ash1l	1.108127733	0.148124189	6.919764814	0.0129
A_51_P264995	Mtf2	1.108212172	0.148234119	7.808125148	0.0076
A_52_P296913	Cnnm1	1.10911223	0.149405358	10.81097588	0.0118
A_51_P293901	Dhrs1	1.110838798	0.151649472	8.053468511	0.0186
A_52_P558713	4930414L22Rik	1.110935993	0.151775698	6.554407526	0.0367
A_52_P487598	Ncor1	1.112147332	0.153347922	7.548996914	0.0263
A_51_P427017	1700020I14Rik	1.112212325	0.15343223	7.529301789	0.0086
A_52_P247733	Prune	1.112333581	0.153589507	6.872993227	0.0381
A_52_P596357	NA	1.11236036	0.153624239	6.759140893	0.0443
A_52_P513439	NA	1.11319569	0.154707227	7.038950335	0.0386
A_52_P52263	D17Wsu92e	1.113529151	0.155139327	7.419442866	0.0334
A_51_P503877	NA	1.113632347	0.155273021	7.936411269	0.0198
A_52_P185664	Nipal4	1.113990024	0.155736313	7.403786125	0.0065
A_52_P654108	Dync1li2	1.114046438	0.155809372	7.438670867	0.0354
A_52_P113250	Insig2	1.114743023	0.15671117	7.780341089	0.0347
A_51_P174996	Slc17a6	1.114884714	0.156894534	9.661117151	0.0160
A_51_P247614	Ncrna00086	1.115997395	0.15833366	8.624346081	0.0430
A_52_P328044	Tle1	1.11609064	0.158454196	8.546660005	0.0097
A_51_P443322	Eif3c	1.117044489	0.159686645	8.80385483	0.0434
A_52_P507479	Fam73a	1.117531203	0.160315114	8.032553514	0.0145
A_51_P295022	Nedd4	1.117669233	0.160493295	10.68749727	0.0500
A_51_P480855	Rad18	1.117745696	0.160591991	7.204079109	0.0126
A_52_P69867	Ppme1	1.118136728	0.161096614	7.282451887	0.0356
A_51_P383599	NA	1.118294613	0.161300314	7.976652341	0.0233
A_51_P221510	Fam81a	1.118661088	0.16177302	7.19119251	0.0486
A_52_P407871	Lsm12	1.118875006	0.162048876	7.501752281	0.0103
A_51_P206153	Ptprd	1.11919689	0.162463858	10.866322	0.0339
A_51_P156882	Adarb1	1.119574587	0.162950645	7.020148601	0.0044
A_52_P402989	H2afy	1.119798142	0.163238692	7.658984475	0.0311
A_52_P565940	Nsd1	1.119897579	0.163366795	7.255314113	0.0130
A_51_P201390	NA	1.120018467	0.16352252	6.777798895	0.0066
A_51_P234544	Azin1	1.120302472	0.1638883	8.682072395	0.0380
A_51_P409452	Cldn11	1.120323424	0.163915282	11.68900386	0.0087

A_51_P410581	Hdgfrp3	1.121412423	0.165316957	7.214797808	0.0280
A_52_P131915	Acap2	1.121444401	0.165358096	9.023574054	0.0376
A_52_P205282	Huwe1	1.122695755	0.166967018	7.443012756	0.0150
A_51_P479758	Sp4	1.123626127	0.168162076	6.690540476	0.0031
A_51_P358908	U2af2	1.123841352	0.168438391	6.741590526	0.0102
A_52_P147778	Fgfr1op	1.124041438	0.168695222	6.647933229	0.0102
A_51_P311476	Rgma	1.124777702	0.169639899	10.79795687	0.0479
A_51_P203321	Rbm10	1.124902382	0.169799812	9.14571257	0.0263
A_52_P249424	Vegfa	1.124939165	0.169846985	6.920272007	0.0060
A_51_P196590	Hadh	1.125141415	0.17010634	8.452964723	0.0278
A_52_P378167	Pcdha9	1.125482701	0.170543882	7.032258718	0.0168
A_52_P383913	Trim35	1.125602906	0.170697958	6.512756669	0.0345
A_52_P18299	Chd5	1.125941849	0.171132319	8.164651351	0.0395
A_51_P406527	Kcnd2	1.126028057	0.171242776	7.931109216	0.0048
A_51_P202040	Fam98b	1.126032101	0.171247956	7.558490442	0.0486
A_52_P41175	Med13l	1.126246743	0.171522934	8.120490845	0.0052
A_52_P470373	Nlk	1.126393655	0.171711112	8.759443215	0.0309
A_51_P463791	Srrm3	1.126466748	0.171804728	8.782258289	0.0393
A_52_P529446	NA	1.127499514	0.173126811	7.583891884	0.0339
A_52_P91346	Mier1	1.127836982	0.173558555	7.359142249	0.0022
A_52_P367791	Mri1	1.128529355	0.174443946	7.237866266	0.0238
A_52_P409778	Wdfy3	1.129515391	0.175703929	7.950130064	0.0334
A_51_P396364	Cdk5rap2	1.12955516	0.175754724	7.177488674	0.0140
A_52_P110257	Wdr83	1.129567152	0.17577004	7.58457257	0.0107
A_52_P108952	Ppp2r5a	1.129728355	0.175975917	7.642048084	0.0448
A_52_P448357	Tspyl4	1.130307107	0.17671481	7.44457408	0.0051
A_52_P294174	U2surp	1.1316316	0.178404369	7.061330014	0.0055
A_52_P585028	Cnep1r1	1.132016053	0.178894417	8.164653801	0.0163
A_51_P251245	Pkp4	1.133180936	0.180378236	8.954950047	0.0501
A_52_P482875	Trak2	1.133192675	0.180393182	6.929500709	0.0029
A_52_P391639	1600029I14Rik	1.133777407	0.181137426	6.50932652	0.0200
A_51_P168762	Tnfrsf21	1.134067349	0.18150632	8.977285037	0.0386
A_52_P271725	Rtn3	1.134249156	0.181737586	8.53186647	0.0465
A_51_P229599	Etnk1	1.135103888	0.182824343	7.446990095	0.0466
A_51_P438293	Smarcal1	1.135257538	0.183019616	7.774412093	0.0166
A_52_P247513	Hook3	1.135578748	0.183427755	7.38564258	0.0245
A_51_P310164	2810459M11Rik	1.135658955	0.183529651	6.631043773	0.0028
A_51_P189343	Map7d1	1.136447547	0.184531097	11.15588338	0.0451
A_52_P322658	Ubqln1	1.136981211	0.185208413	8.789657699	0.0269
A_51_P485391	Parn	1.137175188	0.185454527	7.565714788	0.0221
A_51_P441970	Stox2	1.138190901	0.186742551	7.948774003	0.0197
A_52_P535212	Cpeb3	1.138632492	0.187302175	8.024762386	0.0091
A_52_P148428	Nfix	1.138871816	0.187605376	7.0442366	0.0023
A_52_P384574	Stard4	1.140161801	0.189238573	7.876759126	0.0228
A_52_P89305	Frmd5	1.140440777	0.189591529	8.720141537	0.0122

A_52_P622850	Hes5	1.141022816	0.19032764	7.98526301	0.0469
A_51_P438349	Kif1c	1.141783713	0.191289389	8.200979281	0.0048
A_51_P258690	Scrg1	1.141827557	0.191344785	9.905906341	0.0230
A_52_P464570	Wwp1	1.141876545	0.191406681	7.340481265	0.0288
A_51_P317076	Use1	1.142132446	0.191729961	8.63048982	0.0386
A_51_P125825	Dzip1	1.143272736	0.19316961	7.526890454	0.0231
A_52_P568028	Ncdn	1.14455437	0.194785997	9.358803485	0.0386
A_52_P459143	Celf6	1.14473911	0.195018841	7.709897982	0.0065
A_52_P108089	BC030336	1.145139758	0.195523682	9.023113148	0.0253
A_51_P432432	Pcdh9	1.145169626	0.19556131	8.849190334	0.0114
A_52_P461517	Ubap2l	1.145189184	0.19558595	7.450202802	0.0205
A_51_P373142	AI854703	1.145346742	0.195784425	9.619347433	0.0207
A_51_P504037	Smarca2	1.145849278	0.196417288	8.350059829	0.0416
A_52_P160518	Sfmbt1	1.146332247	0.197025247	6.975984214	0.0154
A_52_P529013	Paip2b	1.146727319	0.197522372	6.51314266	0.0385
A_52_P121525	Strbp	1.148112669	0.199264226	6.958952178	0.0186
A_51_P393761	Ndufs2	1.148832416	0.200168362	9.480059624	0.0198
A_51_P146063	Nemf	1.149116647	0.200525253	8.942409071	0.0476
A_51_P385258	Miox	1.149365982	0.200838255	6.558471608	0.0425
A_52_P647393	E130308A19Rik	1.150282201	0.201987844	7.732666236	0.0153
A_51_P193379	Mtmr7	1.151546589	0.20357278	7.075658447	0.0469
A_51_P437068	Cnnm1	1.151609298	0.203651343	8.966903305	0.0014
A_51_P184398	Ttbk2	1.151631829	0.203679567	7.121035973	0.0300
A_52_P471088	Ctage5	1.152344218	0.20457173	7.20034488	0.0008
A_52_P345946	NA	1.152602252	0.204894744	6.766469125	0.0458
A_51_P337269	Aldob	1.152717323	0.205038769	7.101947797	0.0293
A_51_P158400	NA	1.153519141	0.206041944	7.22052715	0.0041
A_51_P473383	Tenm4	1.153760139	0.206343326	10.27050127	0.0286
A_52_P79763	Thrap3	1.154622758	0.207421567	7.99299822	0.0298
A_51_P494430	Id4	1.155695559	0.208761403	9.060728487	0.0382
A_51_P117995	Pfkm	1.157042763	0.210442186	11.16043619	0.0380
A_52_P676108	Rnaseh2a	1.158245711	0.21194134	7.454346739	0.0313
A_52_P355276	Smg6	1.158590479	0.212370715	7.522025566	0.0419
A_52_P429944	Apba1	1.159486939	0.213486569	6.816278071	0.0184
A_51_P256246	Tspan13	1.159539831	0.213552379	10.25891092	0.0374
A_52_P7041	Odc1	1.160217354	0.214395104	9.572466104	0.0377
A_52_P559770	Aplp2	1.160240191	0.214423501	7.699855244	0.0125
A_51_P233367	Fzd10	1.160418994	0.214645815	6.855972104	0.0013
A_52_P53948	Srpr	1.160453291	0.214688455	7.598631024	0.0107
A_52_P96782	Wasl	1.160586005	0.214853437	7.99229398	0.0029
A_51_P169061	Lpcat2	1.16122379	0.215646034	8.400541524	0.0486
A_51_P135416	Mpped2	1.161227067	0.215650105	7.892057777	0.0332
A_51_P205545	Creld1	1.162230513	0.216896236	8.462711018	0.0229
A_51_P412835	Daxx	1.162351091	0.217045904	8.774230572	0.0125
A_52_P64601	Msl1	1.162415408	0.217125732	7.241721538	0.0446

A_52_P454950	Ube2b	1.164010963	0.219104646	9.200084473	0.0257
A_52_P493620	Fgfr1op2	1.164231197	0.219377582	8.943154757	0.0411
A_51_P243900	Nell2	1.165558693	0.221021655	9.135431911	0.0157
A_51_P348325	Poc1b	1.166005402	0.221574473	6.763290377	0.0115
A_51_P182572	Phactr1	1.168588356	0.224766819	10.97076802	0.0324
A_52_P239052	Zfp148	1.169052681	0.225339943	8.821128233	0.0074
A_52_P73703	Dnajc27	1.171438925	0.228281738	9.419298513	0.0030
A_51_P406105	Rps4y2	1.171857503	0.22879715	9.728765059	0.0309
A_52_P646312	Plekha5	1.172033713	0.229014068	8.088474634	0.0126
A_52_P447477	Prepl	1.17225475	0.229286125	9.362909227	0.0086
A_52_P75568	Hspa4l	1.173208212	0.230459075	7.561452244	0.0135
A_51_P326764	Acbd3	1.173413943	0.230712039	7.537829577	0.0193
A_52_P382754	Ncam1	1.173624695	0.230971133	10.32414274	0.0498
A_52_P157150	Rassf4	1.17602721	0.233921441	7.380282963	0.0105
A_52_P399175	Rffl	1.176929052	0.235027354	7.926231236	0.0124
A_51_P257885	Mmd2	1.178881888	0.237419182	9.945632967	0.0032
A_52_P516034	Ptp4a1	1.179474175	0.238143831	8.796148684	0.0340
A_51_P125935	Syt11	1.179840406	0.238591724	10.69588436	0.0413
A_51_P323712	Agt	1.182500494	0.241840786	11.8077946	0.0101
A_52_P513167	Larp4b	1.182520602	0.241865319	7.235803649	0.0328
A_51_P501735	Gria4	1.182863197	0.24228323	8.588995508	0.0019
A_52_P536947	Cyfip2	1.183160693	0.242646029	10.31480834	0.0479
A_51_P340200	G3bp2	1.183411583	0.242951921	10.38375846	0.0484
A_52_P168496	Slc1a2	1.185479647	0.245470894	9.157389192	0.0065
A_52_P641629	Gsk3b	1.185692093	0.245729411	7.280433015	0.0089
A_52_P240152	Snx27	1.187467646	0.247888207	8.358103263	0.0077
A_51_P463789	Srrm3	1.187526668	0.247959912	9.732121121	0.0264
A_51_P454280	Chd4	1.190007151	0.250970243	8.114276243	0.0007
A_52_P82741	Hspa1a	1.190469624	0.251530809	8.2218115	0.0191
A_51_P377237	Kras	1.190898136	0.252050017	7.580098801	0.0063
A_51_P249544	Haus8	1.193622545	0.255346691	8.381915399	0.0160
A_51_P498640	Pdxk	1.19531363	0.257389206	9.207620776	0.0078
A_52_P592305	Kcnc1	1.19533885	0.257419645	8.9397215	0.0437
A_52_P243658	Edil3	1.197187585	0.259649223	7.463260626	0.0056
A_52_P45708	Vezf1	1.197265464	0.25974307	8.724976195	0.0017
A_51_P479659	Eif5b	1.197466842	0.259985708	8.633395821	0.0483
A_52_P317393	Gpr56	1.204563832	0.268510846	8.955796891	0.0019
A_52_P136275	Tgs1	1.205867032	0.270070833	8.279374759	0.0076
A_51_P492528	D3Bwg0562e	1.209901382	0.27488946	8.558949354	0.0283
A_52_P599728	Map1a	1.214205888	0.280013075	8.958328595	0.0035
A_52_P636830	G3bp2	1.216434846	0.282659049	9.820957461	0.0290
A_52_P653585	Gnai1	1.217206876	0.283574389	8.981878886	0.0223
A_51_P419389	Bmpr2	1.218509776	0.285117825	7.805445259	0.0498
A_52_P187855	Trim37	1.220822007	0.287852874	10.25064594	0.0108
A_52_P415365	Fam120a	1.225071882	0.292866403	7.112852139	0.0361

A_52_P846109	Map1a	1.230327776	0.29904272	7.995119245	0.0140
A_51_P518528	Dpy19l1	1.231679953	0.300627426	10.01975596	0.0018
A_52_P58006	Acbd3	1.234179419	0.303552141	7.58521375	0.0177
A_52_P631591	Mast3	1.237306964	0.307203463	8.824236058	0.0442
A_51_P500981	Map7	1.239166462	0.309370003	7.750726126	0.0188
A_51_P102789	C1qc	1.257881858	0.330996429	8.255815989	0.0134
A_52_P155100	Srcin1	1.282454468	0.358907605	11.35226569	0.0417
A_51_P479528	Klhdc10	1.28443613	0.361135152	8.977833602	0.0282
A_52_P289835	Foxn3	1.284727701	0.361462611	8.763636676	0.0074
A_52_P279759	Glg1	1.286170817	0.36308226	7.384041009	0.0014
A_51_P142175	Lancl1	1.291153916	0.368660991	7.51401959	0.0001
A_52_P654965	Eif3j2	1.29133598	0.36886441	7.982257508	0.0187
A_51_P215038	Tmem59l	1.293148059	0.370887466	11.52653294	0.0019
A_52_P573497	Ddx6	1.298558792	0.376911333	7.932720916	0.0019
A_51_P419319	Aqp4	1.307324867	0.386617691	10.1316974	0.0113
A_52_P250517	Zfp106	1.307554575	0.386871163	7.129419128	0.0233
A_51_P509997	Cox6a2	1.345903439	0.428574909	7.663388985	0.0203
A_52_P191567	Plcl1	1.346240062	0.428935695	7.853744491	0.0333
A_51_P419086	Gadd45gip1	1.365883402	0.449834334	9.64219593	0.0444

Positive and negative values correspond to up and down regulation changes, respectively.

Table S2. List containing differentially expressed genes (p<0.05) in 80 days old mice obtained from microarray analysis.

ProbeID	Gene Symbol	Absolute Fold Change	Log Fold Change	Average Expression	P.Value
A_51_P422030	Ocel1	-1.926137195	-0.945710467	7.380022796	0.004
A_52_P567281	NA	-1.802418196	-0.849933783	10.48723334	0.015
A_52_P442031	Klf2	-1.757929594	-0.813877291	11.42533989	0.013
A_52_P527944	Ptprz1	-1.729486407	-0.790343675	14.69646178	0.000
A_52_P586928	Pdyn	-1.679497739	-0.748029853	10.16510915	0.017
A_51_P318830	Syt10	-1.66243188	-0.733295226	8.958153717	0.005
A_52_P549977	Fam32a	-1.617644597	-0.693894677	8.317250618	0.008
A_52_P336748	NA	-1.604941822	-0.682521002	11.6145284	0.001
A_51_P128075	Tescl	-1.5519168	-0.634051215	6.67612467	0.016
A_52_P292251	NA	-1.532726727	-0.616100499	6.791582186	0.044
A_52_P313382	Tfap2e	-1.419602225	-0.505486741	8.82959688	0.049
A_52_P2670	Rmrp	-1.417508964	-0.503357858	7.381494135	0.047
A_51_P180747	Ctla2a	-1.400991151	-0.486447844	7.934491912	0.011
A_51_P440682	Cap1	-1.388451673	-0.473476963	7.262431519	0.038
A_51_P309618	Yae1d1	-1.383809679	-0.468645537	7.006236848	0.000
A_51_P300759	Ppih	-1.381266607	-0.46599181	8.120232642	0.000
A_51_P178083	Resp18	-1.361433559	-0.445126578	10.4724229	0.003
A_52_P187855	Trim37	-1.345616344	-0.428267134	10.06505455	0.011
A_52_P676406	Cdc37l1	-1.320687689	-0.401289345	9.386810779	0.013
A_51_P259118	Klhl1	-1.319513878	-0.400006525	8.413875297	0.002
A_52_P176983	9530080O11Rik	-1.317211804	-0.397487345	10.27860007	0.009

A_52_P513624	NA	-1.316198465	-0.396377044	7.561510257	0.024
A_52_P425634	2610005L07Rik	-1.312715864	-0.39255468	9.013941293	0.008
A_51_P267544	Frg1	-1.308483728	-0.387895983	7.614467992	0.009
A_51_P358894	Ttc9b	-1.303669965	-0.382578686	8.144690401	0.028
A_52_P555537	2810008D09Rik	-1.298972997	-0.377371441	9.599079269	0.003
A_51_P403704	2610100L16Rik	-1.298511238	-0.376858499	7.578343661	0.016
A_52_P94874	Gnas	-1.294467869	-0.372359155	7.463768948	0.017
A_52_P655743	Lsm6	-1.292192803	-0.369821345	10.34870526	0.019
A_52_P846109	Map1a	-1.288507184	-0.365700581	8.006607885	0.018
A_52_P482124	Fam32a	-1.287988029	-0.365119185	8.119495815	0.024
A_51_P449824	Exoc3l2	-1.285364759	-0.362177823	6.550582038	0.016
A_52_P127892	NA	-1.283170792	-0.359713208	7.66589616	0.001
A_51_P383644	Amy1	-1.282838864	-0.359339967	9.219959673	0.025
A_51_P400269	Slc38a5	-1.280588961	-0.356807478	8.289531475	0.003
A_51_P493234	Cp	-1.28044151	-0.356641352	7.491880958	0.046
A_52_P335089	2610005L07Rik	-1.279291893	-0.355345479	7.515241667	0.009
A_52_P258959	NA	-1.275018075	-0.350517699	8.504297755	0.024
A_51_P489522	Ctla2b	-1.274483469	-0.34991266	7.272573401	0.017
A_51_P456465	Cldn10	-1.270406101	-0.345289746	9.55478568	0.025
A_52_P593268	Lsm6	-1.269121132	-0.343829775	10.08061305	0.023
A_52_P654965	Eif3j2	-1.268678859	-0.343326925	8.026880979	0.007
A_52_P477752	Csnk1a1	-1.266366548	-0.340695051	7.95235847	0.003
A_52_P94201	Syt1	-1.26512546	-0.339280461	7.517121897	0.013
A_52_P490874	Srrm4	-1.261495052	-0.335134548	7.439101524	0.001
A_51_P319562	Ank2	-1.260915604	-0.334471716	8.206160891	0.048
A_52_P392456	Rnd3	-1.260901561	-0.334455648	6.403321542	0.002
A_51_P323443	Vapb	-1.260828176	-0.334371681	8.484951047	0.024
A_51_P419389	Bmpr2	-1.257009377	-0.329995412	7.756211883	0.032
A_52_P646312	Plekha5	-1.254382578	-0.326977427	8.27636289	0.042
A_51_P147684	Nr2f2	-1.252326426	-0.324610657	7.225899386	0.041
A_52_P289835	Foxn3	-1.251587844	-0.323759552	8.582021884	0.019
A_51_P227866	Tmx4	-1.247608537	-0.319165329	9.100258112	0.011
A_51_P278018	Vps36	-1.247199457	-0.318692205	9.161045819	0.002
A_52_P588483	Fbln1	-1.246878255	-0.318320608	7.220161224	0.019
A_51_P448458	Dnm3	-1.246042528	-0.317353309	8.340725849	0.019
A_51_P366867	Gas5	-1.245204965	-0.316383235	10.75795389	0.004
A_52_P103929	Syt1	-1.244545289	-0.315618731	9.832449582	0.043
A_52_P423128	Arglu1	-1.235193648	-0.304737239	10.0844939	0.007
A_51_P445487	2410066E13Rik	-1.234625698	-0.304073726	7.525047862	0.022
A_51_P469902	NA	-1.233773985	-0.303078131	7.895272756	0.015
A_52_P306387	Rnf214	-1.231280384	-0.300159326	7.392621092	0.026
A_52_P670399	NA	-1.229474863	-0.298042239	10.47544302	0.000
A_52_P684050	Fam110a	-1.228010841	-0.296323297	6.714388179	0.016
A_51_P269634	Zfp14	-1.226710333	-0.294794621	7.333505148	0.014
A_51_P388587	AY036118	-1.225439893	-0.293299723	10.38182508	0.005

A_52_P683146	Cdh11	-1.222173101	-0.289448634	7.642475688	0.016
A_52_P484838	Rfxank	-1.219505847	-0.286296675	7.251116711	0.017
A_52_P75384	B230219D22Rik	-1.218343286	-0.28492069	8.731694671	0.033
A_51_P377045	Malat1	-1.213035689	-0.278621997	6.947858181	0.014
A_51_P224843	Tmsb4x	-1.211999378	-0.277388958	12.40158994	0.041
A_52_P490863	Nop10	-1.209357326	-0.274240577	10.27543386	0.010
A_51_P351923	A030009H04Rik	-1.208325062	-0.273008619	9.088167808	0.005
A_51_P328769	Rnf20	-1.206884388	-0.271287481	8.596512199	0.030
A_51_P204831	Crip1	-1.206237313	-0.270513769	7.110367028	0.024
A_51_P243596	Mllt6	-1.205499707	-0.2696313	6.719634729	0.032
A_52_P997449	NA	-1.203613133	-0.267371753	7.116169877	0.003
A_51_P160625	Wapal	-1.201231429	-0.264514127	7.809250055	0.025
A_52_P199905	Slc27a1	-1.200134033	-0.263195537	8.39802795	0.003
A_51_P184024	Tsen15	-1.200019737	-0.263058135	9.122772489	0.031
A_51_P114462	Ccl17	-1.198968516	-0.261793775	6.399026857	0.041
A_51_P476820	Calr3	-1.197903921	-0.260512201	6.698374672	0.034
A_51_P516833	Igf2	-1.196682325	-0.25904022	7.67443927	0.022
A_51_P133953	NA	-1.19643136	-0.258737631	9.159972806	0.018
A_51_P296775	NA	-1.196124679	-0.258367777	10.0854274	0.007
A_52_P5394	1810022K09Rik	-1.194766307	-0.256728458	9.883614221	0.007
A_51_P441263	Rpl37a	-1.193972975	-0.255770182	12.1711159	0.014
A_52_P573497	Ddx6	-1.193944312	-0.255735548	7.80357614	0.004
A_51_P463789	Srrm3	-1.192897791	-0.254470436	9.587991167	0.020
A_51_P305843	Chordc1	-1.192029846	-0.253420358	8.382468622	0.002
A_52_P205282	Huwe1	-1.191565415	-0.252858156	7.324053052	0.043
A_51_P337662	Ddx26b	-1.191562197	-0.252854259	9.030350278	0.043
A_51_P441942	Tial1	-1.191345725	-0.252592139	9.861748833	0.019
A_51_P317443	Cd3eap	-1.190534179	-0.251609039	8.069760011	0.033
A_52_P163820	2810006K23Rik	-1.189848731	-0.250778171	6.988481155	0.024
A_51_P460710	Tdrd3	-1.189155799	-0.249937745	8.290330903	0.008
A_52_P26976	Rbm28	-1.188151111	-0.248718332	7.443662957	0.015
A_52_P239023	Zfp955a	-1.187666777	-0.248130117	7.058780904	0.018
A_51_P337708	Ovgp1	-1.186589713	-0.246821181	7.903334269	0.014
A_52_P124812	Usp15	-1.186550829	-0.246773904	7.787086065	0.006
A_52_P240152	Snx27	-1.185609522	-0.245628939	8.133965308	0.047
A_51_P459240	Gstk1	-1.184776589	-0.244615038	8.902797691	0.017
A_52_P387458	Slirp	-1.182019711	-0.241254094	6.968040383	0.009
A_51_P216702	Eogt	-1.181957394	-0.241178032	6.819174729	0.005
A_51_P160664	Cox7b	-1.181933907	-0.241149363	10.90904971	0.001
A_52_P323111	Cers6	-1.181498509	-0.240617809	8.625330244	0.016
A_52_P481880	Rpl36a	-1.175992679	-0.233879079	11.41342863	0.004
A_52_P247513	Hook3	-1.174753582	-0.232358166	7.377469147	0.030
A_52_P196458	Dzip1	-1.174608631	-0.232180144	8.151559892	0.033
A_52_P27725	D930016D06Rik	-1.174310089	-0.231813418	7.145653668	0.017
A_51_P178646	Rpp21	-1.174287582	-0.231785767	10.04771198	0.031

A_51_P357195	NA	-1.173189134	-0.230435614	6.828033273	0.004
A_51_P374549	Spata1	-1.171791901	-0.228716383	6.502249349	0.003
A_52_P55053	Ssbp1	-1.171460276	-0.228308034	7.868696033	0.002
A_51_P132625	Hsd17b11	-1.171299055	-0.228109471	6.760097711	0.033
A_52_P9437	Psmb3	-1.171243151	-0.228040611	11.3348882	0.048
A_52_P295201	Rpl41	-1.170699274	-0.227370528	12.02490115	0.021
A_51_P449795	Crip1	-1.170062097	-0.226585098	7.216899521	0.010
A_52_P253317	NA	-1.168385724	-0.224516635	8.291129159	0.015
A_52_P466147	Rarres2	-1.168369512	-0.224496618	8.375404078	0.035
A_52_P129624	Dgkk	-1.168033938	-0.224082194	7.065185502	0.014
A_51_P199367	Esd	-1.16723927	-0.223100327	10.47249636	0.008
A_51_P246215	Polr2i	-1.166561576	-0.22226246	9.653881627	0.015
A_51_P431047	St8sia3	-1.166260449	-0.221890007	8.293933953	0.016
A_52_P566681	Gpm6a	-1.164841012	-0.220133057	10.86561356	0.023
A_51_P282609	Grik1	-1.163297831	-0.218220507	6.85141025	0.014
A_51_P146063	Nemf	-1.162696379	-0.217474408	8.928338983	0.009
A_52_P61774	Cinp	-1.1626408	-0.217405442	6.745356907	0.015
A_52_P457411	Ubl5	-1.162316488	-0.217002955	10.4591209	0.023
A_51_P318104	App	-1.16203245	-0.216650357	10.117858	0.041
A_52_P469939	Gcc2	-1.161902202	-0.216488641	7.142932462	0.004
A_51_P133737	Luc7l3	-1.161811204	-0.216375647	11.45114057	0.011
A_51_P511199	Rps27	-1.161592256	-0.216103741	11.35063731	0.010
A_51_P225048	Zranb1	-1.161507813	-0.215998858	7.117314676	0.006
A_51_P393161	Scaper	-1.161223499	-0.215645672	7.673192146	0.015
A_52_P79763	Thrap3	-1.161206953	-0.215625116	7.943261048	0.008
A_51_P408881	Pdlim5	-1.160893109	-0.21523514	6.508101588	0.042
A_52_P387598	BC023202	-1.160662041	-0.214947953	8.724028328	0.020
A_52_P481202	Dpp8	-1.160402708	-0.214625568	7.195671995	0.050
A_52_P37894	Cox7a2	-1.160222273	-0.214401221	11.04212857	0.006
A_51_P305437	Rcn1	-1.160103589	-0.214253633	8.17737509	0.023
A_52_P263518	Gng2	-1.160039594	-0.214174048	8.375000867	0.021
A_51_P512210	Myh6	-1.159874413	-0.213968604	8.237198938	0.018
A_51_P193475	Ccdc88a	-1.158999694	-0.212880185	8.07139233	0.035
A_51_P100787	Snw1	-1.158677616	-0.212479215	8.939783219	0.023
A_51_P270478	Pin4	-1.158120317	-0.211785143	9.228154793	0.017
A_51_P117162	Cbx3	-1.15788857	-0.211496421	8.154163704	0.011
A_52_P684857	Srek1	-1.157536468	-0.211057646	7.48773889	0.031
A_51_P414653	Plvap	-1.157349602	-0.210824727	7.281910965	0.019
A_51_P220723	B230118H07Rik	-1.156497642	-0.209762324	11.29227661	0.018
A_52_P599728	Map1a	-1.15637329	-0.209607191	8.879741631	0.022
A_51_P472241	B9d1	-1.155954668	-0.209084823	8.354426648	0.028
A_52_P643359	Prpf4b	-1.155920992	-0.209042791	6.878477183	0.004
A_51_P230382	Etv4	-1.155903376	-0.209020806	6.50671192	0.004
A_51_P518470	Ttc14	-1.155824721	-0.208922632	7.825806609	0.050
A_52_P545643	AI597468	-1.155723101	-0.208795784	8.589480757	0.049

A_51_P465082	Tox3	-1.154392061	-0.207133283	6.95469469	0.011
A_52_P62775	A230057D06Rik	-1.154183707	-0.206872871	6.706278202	0.022
A_51_P225832	2700097O09Rik	-1.15381166	-0.206407748	7.767983123	0.003
A_52_P474949	Chd1	-1.153804454	-0.206398738	6.946662987	0.002
A_52_P629112	NA	-1.153633915	-0.206185483	9.721524874	0.025
A_51_P250465	Mettl5	-1.153509061	-0.206029337	7.628091512	0.038
A_51_P250358	Prpf39	-1.153258833	-0.205716342	7.238897488	0.013
A_51_P323620	Thyn1	-1.152420586	-0.204667337	10.76870925	0.040
A_51_P143142	Mrpl12	-1.151943756	-0.204070279	10.37611529	0.014
A_52_P197926	Rpl36a	-1.151609052	-0.203651035	11.18613843	0.005
A_52_P123384	Cib1	-1.151598048	-0.203637248	8.508748048	0.021
A_52_P431894	Pknox1	-1.151570918	-0.20360326	7.28381146	0.018
A_51_P149818	Srsf11	-1.151539284	-0.203563628	8.39055076	0.036
A_51_P212164	Swt1	-1.151484019	-0.203494389	7.144861713	0.008
A_51_P334570	Uba52	-1.151207833	-0.203148313	12.04814021	0.044
A_52_P535012	NA	-1.150689624	-0.202498747	10.69684422	0.011
A_51_P131164	Enkur	-1.150376736	-0.202106405	6.160920892	0.004
A_52_P599317	Hs6st2	-1.149709553	-0.201269444	9.163054784	0.036
A_51_P234544	Azin1	-1.149327692	-0.200790192	8.686949975	0.022
A_52_P582394	Mrps11	-1.148912395	-0.200268797	7.752531873	0.003
A_51_P441091	NA	-1.148826638	-0.200161107	7.669741203	0.025
A_51_P175988	Htr3a	-1.148553902	-0.199818565	7.241106862	0.028
A_52_P241032	NA	-1.148395819	-0.199619983	10.88372322	0.030
A_52_P112791	Far1	-1.147977054	-0.199093805	8.717351323	0.021
A_52_P569218	Utrn	-1.147713775	-0.198762897	8.968890269	0.037
A_52_P595537	Mrpl47	-1.147530392	-0.198532364	6.846736349	0.029
A_51_P310821	Hoxa5	-1.147126487	-0.198024478	7.33968386	0.003
A_51_P167535	Fabp3	-1.14666985	-0.197450069	9.081337825	0.048
A_51_P449911	Cnot6	-1.146599841	-0.197361984	8.663303922	0.010
A_52_P212597	Hook1	-1.145862675	-0.196434156	6.872595842	0.046
A_51_P434527	NA	-1.145381369	-0.195828042	6.884036065	0.006
A_51_P159565	Arhgef9	-1.145339536	-0.195775348	7.962492736	0.033
A_52_P657324	Rnf32	-1.145325922	-0.195758201	7.260343148	0.026
A_51_P138141	Angel2	-1.145210779	-0.195613155	7.871262311	0.021
A_52_P358963	Hmg20b	-1.145059226	-0.195422221	7.960367765	0.049
A_51_P117752	Asgr1	-1.144747286	-0.195029144	6.874326897	0.022
A_51_P225186	Calcrl	-1.144346649	-0.194524144	7.614539416	0.022
A_51_P509489	Kras	-1.144093767	-0.194205297	9.3315898	0.027
A_51_P127841	Pdss1	-1.143953697	-0.194028658	7.13265224	0.014
A_52_P540045	NA	-1.143835914	-0.193880109	6.630440802	0.009
A_51_P387379	Tshz3	-1.143809668	-0.193847005	7.875456841	0.040
A_51_P461404	Smarca1	-1.143779993	-0.193809575	7.543397144	0.015
A_52_P663303	Wdr60	-1.143502738	-0.19345982	7.453372878	0.032
A_52_P379126	Arhgef9	-1.143497754	-0.193453532	7.060220831	0.028
A_51_P299195	Hnrnph1	-1.142847988	-0.192633521	9.475976384	0.034

A_52_P445387	Clk4	-1.142354967	-0.192011013	9.143354994	0.012
A_52_P521507	NA	-1.142055676	-0.191632985	8.67443817	0.040
A_52_P249424	Vegfa	-1.141763192	-0.191263459	6.696997222	0.026
A_52_P376841	NA	-1.141575687	-0.191026515	7.60080186	0.032
A_51_P275989	Ccdc107	-1.141368826	-0.190765065	7.544263402	0.013
A_52_P118100	NA	-1.14130678	-0.190686636	9.278233725	0.048
A_51_P482473	Rps17	-1.141225459	-0.190583836	11.01786759	0.016
A_52_P212336	2610005L07Rik	-1.141170162	-0.190513931	7.527029348	0.019
A_51_P168459	Ifitm2	-1.141072834	-0.19039088	7.008403933	0.012
A_52_P286928	NA	-1.140983335	-0.19027772	7.233407613	0.018
A_52_P258439	Mau2	-1.140898543	-0.190170503	6.567777278	0.027
A_52_P515347	Tusc3	-1.140800802	-0.190046901	8.562399928	0.023
A_52_P365741	Cdc37l1	-1.140727922	-0.189954732	9.02069311	0.040
A_52_P623690	NA	-1.140140086	-0.189211096	10.46940575	0.048
A_51_P128287	2010107E04Rik	-1.140044648	-0.189090327	10.52273491	0.039
A_51_P278353	1700049J03Rik	-1.13991826	-0.188930376	7.610460437	0.036
A_52_P313789	Ppm1a	-1.1396927	-0.188644877	8.504791483	0.013
A_51_P312348	Krt7	-1.139685396	-0.188635631	8.335489865	0.027
A_52_P440627	Nek1	-1.139367632	-0.188233327	7.5834734	0.027
A_51_P479758	Sp4	-1.139173197	-0.187987108	6.571375699	0.029
A_51_P476783	Sptan1	-1.138439096	-0.187057113	6.738626692	0.047
A_52_P319774	Kcnip4	-1.138260998	-0.186831399	8.697913936	0.033
A_51_P221449	Msi2	-1.138102952	-0.186631069	6.913747722	0.043
A_52_P442986	NA	-1.137772653	-0.186212311	6.750031137	0.027
A_51_P479659	Eif5b	-1.137536556	-0.185912909	8.652556264	0.011
A_52_P582309	Nub1	-1.13752296	-0.185895665	8.594068441	0.045
A_52_P336171	Gabra2	-1.13635638	-0.184415358	8.276381988	0.046
A_51_P221510	Fam81a	-1.136221918	-0.184244638	7.055749484	0.017
A_51_P485756	Nts	-1.135966648	-0.183920477	8.655311718	0.015
A_52_P351574	Plcb1	-1.135861911	-0.183787454	7.73192567	0.025
A_51_P280532	Supt16	-1.135835841	-0.183754342	7.124089711	0.045
A_51_P180754	Map2	-1.135718496	-0.183605287	6.755292906	0.022
A_51_P397426	Timm8b	-1.135429029	-0.183237532	10.8874128	0.034
A_52_P617817	Hspa4	-1.135310904	-0.183087432	6.899260799	0.018
A_51_P377237	Kras	-1.134782829	-0.182416226	7.514925485	0.028
A_51_P474367	Ptges3	-1.134376662	-0.181899756	9.524373527	0.009
A_52_P62530	NA	-1.133740219	-0.181090104	6.742489678	0.041
A_51_P101777	Herc4	-1.133291335	-0.180518782	7.700700653	0.015
A_52_P193440	NA	-1.133037374	-0.18019545	6.389705636	0.040
A_51_P237752	Ptrf	-1.132909777	-0.180032971	9.298255121	0.032
A_51_P200134	Snrpg	-1.132717812	-0.179788495	9.857414773	0.022
A_51_P420547	Clic5	-1.132672035	-0.17973019	7.044348706	0.047
A_52_P380649	Prkrir	-1.13261695	-0.179660025	7.227047511	0.031
A_51_P348624	Timm10	-1.132454297	-0.179452828	10.60929475	0.016
A_51_P118715	Rspry1	-1.132313635	-0.17927362	7.599444868	0.018

A_51_P431046	St8sia3	-1.132039995	-0.178924929	7.816732027	0.040
A_52_P5549	Fam133b	-1.131940069	-0.178797577	7.278076737	0.014
A_51_P108478	Mrps18c	-1.13191978	-0.178771717	8.685696178	0.029
A_52_P26626	Fam92b	-1.131819635	-0.17864407	7.001057755	0.034
A_51_P507899	Ttc8	-1.131746567	-0.178550931	7.092947536	0.021
A_51_P478061	Cadm1	-1.13133589	-0.178027325	8.931351368	0.039
A_52_P287219	Man2a2	-1.130960612	-0.177548685	9.807215691	0.021
A_51_P242399	Krt8	-1.130757831	-0.177289987	7.34066702	0.027
A_52_P346231	Azi2	-1.130485496	-0.176942483	7.488017217	0.039
A_51_P219109	Il12rb1	-1.130416563	-0.176854509	6.479155897	0.049
A_52_P558259	Dtna	-1.129930005	-0.176233405	8.278734924	0.021
A_51_P153787	Fcf1	-1.129861311	-0.176145694	8.250603608	0.048
A_51_P238933	Nudc	-1.12977608	-0.176036861	12.38535303	0.014
A_51_P261107	Ogt	-1.129290036	-0.175416062	8.017240697	0.013
A_51_P279997	Slc4a7	-1.128860472	-0.174867178	7.198206194	0.041
A_51_P249867	Snrnp48	-1.128834422	-0.174833886	7.641202008	0.013
A_51_P465292	Hnmt	-1.128712485	-0.174678037	7.677007035	0.050
A_52_P67444	Arih1	-1.128641495	-0.174587296	6.722595526	0.044
A_52_P544523	Myl4	-1.128505671	-0.174413669	7.810172012	0.043
A_51_P459873	6330411E07Rik	-1.128235252	-0.174067921	6.609988073	0.017
A_52_P356093	B3galt2	-1.128047417	-0.173827712	6.873754357	0.047
A_51_P141772	Uhrf2	-1.127862336	-0.173590987	7.940298257	0.048
A_52_P650215	Nop14	-1.127829037	-0.173548393	6.956401632	0.031
A_52_P301223	Brwd1	-1.127594937	-0.173248905	8.177733988	0.026
A_52_P24320	Rpgrip1l	-1.127574666	-0.17322297	6.352953219	0.012
A_52_P573161	Rpl32	-1.127241161	-0.172796197	11.75633282	0.015
A_51_P416869	Nedd8	-1.127228014	-0.172779371	11.24034471	0.021
A_52_P177161	NA	-1.127045364	-0.172545586	6.888991696	0.033
A_52_P306396	Ppp3cb	-1.126548559	-0.171909502	9.126236572	0.034
A_51_P170156	Ndufa5	-1.126499354	-0.171846486	12.09172294	0.030
A_51_P270364	Mmaa	-1.126088692	-0.171320461	9.003559632	0.033
A_51_P314521	Smim11	-1.125960242	-0.171155887	8.74253513	0.027
A_51_P337020	Senp7	-1.125539285	-0.170616413	6.592726426	0.048
A_51_P280192	Tmem256	-1.125481397	-0.17054221	8.56244029	0.010
A_52_P140497	Golga3	-1.125185463	-0.170162819	7.729240598	0.044
A_51_P255565	Smarcad1	-1.125004272	-0.169930479	6.78616172	0.012
A_51_P259064	Ube2d3	-1.12461685	-0.169433567	8.978003742	0.032
A_52_P402960	B230337E12Rik	-1.124329998	-0.169065538	8.001259363	0.029
A_52_P124105	Rab14	-1.124016111	-0.168662714	6.459482502	0.017
A_52_P270145	Zfp329	-1.123111853	-0.167501616	8.717921823	0.033
A_52_P533402	Zfp607	-1.122941368	-0.167282602	7.573627007	0.049
A_51_P160744	Ndufb3	-1.12273191	-0.167013476	11.51134416	0.011
A_51_P353592	Commd4	-1.122520927	-0.166742342	9.225583363	0.026
A_51_P110341	Scgb3a1	-1.122179378	-0.166303306	7.321426146	0.037
A_51_P337290	Rps19	-1.122112696	-0.166217577	7.345320635	0.045

A_51_P219542	Gnpnat1	-1.122014193	-0.166090926	6.887871956	0.010
A_52_P522157	Snhg5	-1.121469537	-0.165390432	8.186611813	0.035
A_52_P563340	NA	-1.121168742	-0.165003428	8.634494808	0.026
A_52_P502141	Hectd2	-1.121121184	-0.164942231	6.583093709	0.014
A_52_P120842	Man1a2	-1.120999606	-0.164785772	7.28694719	0.042
A_52_P507310	Mrps24	-1.120948722	-0.164720283	9.183253342	0.038
A_52_P234910	NA	-1.120720925	-0.164427071	8.326941923	0.044
A_52_P451378	Twsg1	-1.120450119	-0.164078424	6.664396942	0.017
A_51_P159122	Kndc1	-1.1202907	-0.16387314	6.312034979	0.033
A_51_P184806	Elmod2	-1.119792076	-0.163230876	8.024930183	0.020
A_51_P321086	Amz2	-1.119679872	-0.16308631	8.945840095	0.014
A_51_P261001	Mtf2	-1.119613552	-0.163000855	8.366050799	0.046
A_51_P336790	Lsm5	-1.119545573	-0.162913257	7.89845894	0.040
A_51_P464149	Fam45a	-1.119483135	-0.162832795	7.785234314	0.045
A_52_P845245	Gnai2	-1.119297584	-0.162593652	6.376891738	0.005
A_52_P672960	Camk4	-1.119238592	-0.162517614	7.059889378	0.025
A_52_P250578	Fam115a	-1.119227028	-0.162502707	8.424525168	0.046
A_52_P412465	Bach1	-1.118838087	-0.162001271	6.877997049	0.043
A_51_P129929	Zfp866	-1.118422681	-0.161465523	6.479179738	0.013
A_51_P501735	Gria4	-1.118155227	-0.161120483	8.631713842	0.034
A_51_P314323	Lsmd1	-1.118099038	-0.161047983	11.198031	0.021
A_52_P336594	NA	-1.117889475	-0.160777557	6.374117543	0.041
A_52_P394755	Usp45	-1.117636264	-0.160450737	6.673656489	0.046
A_51_P189905	NA	-1.117566671	-0.160360901	9.575172432	0.037
A_52_P72354	NA	-1.117411129	-0.160160094	8.524396252	0.049
A_51_P351481	Ccnl1	-1.117215751	-0.159907819	6.966086822	0.027
A_52_P396917	Eml5	-1.117151018	-0.159824225	6.955258696	0.044
A_52_P518434	Hmbox1	-1.117126718	-0.159792842	6.443631283	0.012
A_52_P406864	Fam179b	-1.116854229	-0.159440899	8.104739275	0.048
A_51_P205278	Tmem161b	-1.116765273	-0.159325986	6.78426147	0.016
A_51_P445555	NA	-1.11659476	-0.159105691	8.503538069	0.037
A_52_P661731	2-Mar	-1.115940513	-0.158260124	9.289888234	0.031
A_51_P320980	Sfxn4	-1.115660846	-0.157898523	7.259281687	0.019
A_51_P117664	NA	-1.115217939	-0.157325673	9.261198344	0.030
A_51_P482600	Btf3	-1.115207411	-0.157312054	6.600784706	0.008
A_52_P394111	NA	-1.114970277	-0.157005251	7.804702078	0.036
A_51_P128786	4833424O15Rik	-1.114552468	-0.156464533	6.908471303	0.038
A_52_P384392	Tsc22d4	-1.114474201	-0.15636322	6.964310559	0.040
A_52_P319123	NA	-1.114205757	-0.156015676	7.272787079	0.044
A_51_P366277	Nol8	-1.113612265	-0.155247006	8.089577351	0.046
A_51_P199987	Gucy1a3	-1.11346624	-0.155057816	7.86653418	0.016
A_51_P407209	Rapgef6	-1.113364242	-0.154925654	6.98303772	0.026
A_51_P210350	Slc17a8	-1.113264582	-0.154796509	7.60501457	0.033
A_51_P274768	Rps25	-1.112331975	-0.153587425	11.81583536	0.041
A_51_P112662	Sp3	-1.112273301	-0.153511322	8.389924807	0.038

A_51_P262230	Ggact	-1.111944976	-0.153085399	7.075837091	0.022
A_52_P348522	NA	-1.111794145	-0.152889689	7.647417009	0.024
A_52_P551526	NA	-1.111642746	-0.152693217	11.25502363	0.048
A_51_P404077	Fzd2	-1.111605426	-0.152644782	6.924787524	0.040
A_52_P369271	Zfp280d	-1.111603268	-0.152641981	6.427820807	0.019
A_52_P941128	NA	-1.111032944	-0.151901596	6.375254795	0.041
A_52_P260864	Arhgap5	-1.11079821	-0.151596758	8.545378006	0.045
A_51_P169567	Nfkbil1	-1.110769496	-0.151559463	7.713672879	0.017
A_52_P78922	NA	-1.110707792	-0.151479318	6.891819772	0.045
A_51_P328963	Ufd1l	-1.110171286	-0.150782284	7.400269793	0.041
A_52_P603740	Fbxo33	-1.110026698	-0.150594376	8.469628196	0.024
A_51_P132715	Rpl35	-1.110020318	-0.150586084	11.28489099	0.035
A_52_P571350	H19	-1.110002205	-0.150562543	6.604468102	0.050
A_52_P795474	NA	-1.109813337	-0.150317046	6.15317776	0.049
A_52_P98452	Hadh	-1.109672129	-0.150133471	7.165618389	0.032
A_51_P192783	Ccdc167	-1.108947942	-0.149191641	7.292610655	0.017
A_51_P391871	N4bp2l2	-1.10894489	-0.149187671	6.377034096	0.031
A_51_P142046	Oxld1	-1.108649576	-0.148803428	8.01767977	0.026
A_52_P531175	Fkbp3	-1.108627065	-0.148774133	11.07716807	0.024
A_52_P268134	NA	-1.108155129	-0.148159857	8.678044607	0.038
A_52_P181394	NA	-1.108077128	-0.148058304	6.631019465	0.029
A_52_P65286	Lrp1b	-1.107814826	-0.147716752	6.200877347	0.017
A_51_P142113	Bloc1s1	-1.1077389	-0.14761787	9.235167829	0.021
A_51_P197378	Atg12	-1.107035334	-0.14670127	9.569618758	0.043
A_51_P202623	Mterfd3	-1.106216114	-0.145633263	8.46354557	0.047
A_51_P396708	Med21	-1.106035721	-0.14539798	8.280716498	0.026
A_52_P6404	NA	-1.105915799	-0.145241548	6.527462943	0.024
A_51_P321579	NA	-1.105779274	-0.145063436	8.187156138	0.050
A_52_P350301	NA	-1.105292544	-0.144428267	10.64091028	0.032
A_52_P654604	NA	-1.104819866	-0.143811166	6.703490344	0.041
A_51_P199435	NA	-1.104334638	-0.143177407	7.150255652	0.033
A_51_P416152	Cartpt	-1.104106905	-0.142879868	8.195193478	0.023
A_51_P361788	Vapa	-1.104050081	-0.142805616	11.05622746	0.037
A_52_P571403	Minos1	-1.103806755	-0.14248762	10.7645778	0.048
A_51_P475891	Trnau1ap	-1.103000591	-0.141433564	9.397044645	0.039
A_51_P495171	Pbdc1	-1.102627602	-0.140945621	8.518711896	0.048
A_51_P488718	Zfand2b	-1.102192024	-0.140375593	8.570420153	0.034
A_52_P259184	Gabra1	-1.102105556	-0.140262407	6.665881199	0.041
A_51_P155323	Hc	-1.102016325	-0.140145595	6.740694593	0.031
A_52_P97417	Tubgcp5	-1.101940681	-0.140046563	7.46326058	0.031
A_51_P279851	Dhps	-1.101907502	-0.140003124	8.308375939	0.032
A_51_P240363	Zfp422	-1.101701647	-0.139733578	7.388672193	0.026
A_52_P6070	Eva1c	-1.101438499	-0.139388942	6.330738854	0.036
A_52_P268549	Rhno1	-1.101304155	-0.139212963	8.608618827	0.042
A_52_P269461	NA	-1.100956093	-0.138756935	7.262276347	0.041

A_51_P478952	N4bp2l1	-1.100656572	-0.138364388	6.767365186	0.049
A_51_P505719	Dmxl1	-1.099902032	-0.137375029	6.856463502	0.044
A_52_P565940	Nsd1	-1.099248296	-0.136517296	7.128536928	0.042
A_52_P556602	NA	-1.099201214	-0.136455503	6.325764589	0.020
A_51_P214449	Polr2k	-1.098881595	-0.136035944	9.111412629	0.046
A_52_P338479	NA	-1.098712963	-0.135814533	6.536879175	0.046
A_51_P218814	Rpl22l1	-1.09819815	-0.135138387	11.44585856	0.049
A_51_P222543	NA	-1.097690029	-0.134470716	7.555384042	0.024
A_52_P537907	Tsga10	-1.097628212	-0.134389468	6.536712541	0.039
A_52_P3754	Immp1l	-1.097620387	-0.134379183	7.381037942	0.022
A_51_P147064	1600014C23Rik	-1.097246025	-0.133887043	6.270034354	0.028
A_52_P405340	Fert2	-1.097179756	-0.133799908	6.29719653	0.019
A_52_P463578	Mphosph10	-1.097079842	-0.133668525	7.295727259	0.043
A_51_P369381	Slc18b1	-1.096776301	-0.133269303	7.0553129	0.020
A_51_P372312	Mrpl54	-1.096614601	-0.133056587	9.302238176	0.027
A_51_P129360	Pthlh	-1.0962087	-0.13252249	6.502875347	0.033
A_52_P303041	Tma7	-1.09593566	-0.132163104	10.92822357	0.028
A_52_P316405	NA	-1.095709555	-0.131865426	9.710049097	0.043
A_51_P201254	NA	-1.095364592	-0.13141115	7.994563271	0.045
A_52_P527637	Stx1b	-1.095030496	-0.130971049	6.832131343	0.032
A_52_P210206	Vcpip1	-1.094833536	-0.130711532	6.752396363	0.020
A_52_P418014	Akt1	-1.09453199	-0.13031412	6.227028651	0.015
A_51_P101719	Ttc14	-1.094114598	-0.129763855	7.526738438	0.036
A_52_P345548	Pcdha4-g	-1.093925751	-0.12951482	6.606730645	0.044
A_52_P91346	Mier1	-1.093535443	-0.128999981	7.27884576	0.023
A_51_P253633	Mrps9	-1.093166185	-0.128512738	8.645612409	0.027
A_51_P315754	Dis3	-1.09312456	-0.128457803	7.345996076	0.041
A_52_P14938	NA	-1.092919017	-0.128186505	6.567186009	0.042
A_52_P294174	U2surp	-1.092490445	-0.127620662	7.005546833	0.048
A_51_P227962	Dynlrb2	-1.092444771	-0.127560345	7.429798654	0.049
A_51_P520857	Gm12060	-1.092080927	-0.127079769	10.94865332	0.036
A_52_P521710	Dph3	-1.091101509	-0.125785326	8.63337876	0.042
A_51_P519276	Ndufb7	-1.09107238	-0.125746811	11.78595956	0.045
A_51_P312175	Tnks	-1.090685602	-0.125235295	6.303073861	0.036
A_51_P100099	2610002J23Rik	-1.090125398	-0.124494099	8.637492437	0.047
A_52_P642012	BC006965	-1.088931928	-0.122913771	6.252032412	0.031
A_52_P617020	Ap3s1	-1.088312059	-0.12209229	8.590108507	0.037
A_52_P65506	Cxxc4	-1.088084621	-0.12179076	7.329018387	0.040
A_51_P118720	Rspry1	-1.087540891	-0.121069646	7.374478486	0.038
A_52_P371063	Iqcc	-1.086058134	-0.119101329	6.179235334	0.040
A_52_P411780	Hdac9	-1.085216602	-0.117983024	6.560549142	0.045
A_52_P641597	Zc3h7a	-1.084139537	-0.116550455	6.42105274	0.047
A_52_P660400	Grik1	-1.083795054	-0.116091968	6.230069066	0.045
A_52_P315280	Nktr	-1.083427169	-0.115602174	6.494034388	0.044
A_51_P193682	1700012B09Rik	-1.081634369	-0.113212899	6.216830517	0.044

A_51_P419226	S100a14	-1.080749037	-0.112031551	6.429996868	0.049
A_51_P300506	Cox6b2	-1.079776027	-0.110732092	6.46165825	0.047
A_51_P121288	Mkks	-1.079581097	-0.110471621	6.425789597	0.049
A_52_P160057	Gm3373	-1.07862991	-0.109199944	6.798656392	0.048
A_51_P371091	Rcsd1	-1.075768072	-0.105367077	7.178465589	0.047
A_52_P241742	2010003O02Rik	-1.075433766	-0.104918675	6.868355907	0.046
A_51_P443359	Trappc2l	-1.07225045	-0.100641922	9.375651696	0.049
A_52_P328304	Plekhg3	1.074845567	0.104129389	8.724389267	0.040
A_51_P354572	Rab35	1.079370648	0.110190361	9.932712757	0.048
A_52_P462013	Cln8	1.080872673	0.112196583	6.324714826	0.045
A_51_P276599	AU040320	1.082183015	0.113944503	8.977725945	0.045
A_52_P185343	Gna13	1.083176563	0.115268429	6.707743059	0.039
A_52_P668030	Mrpl44	1.083310782	0.115447185	6.755086607	0.047
A_52_P609965	Zmynd11	1.084770461	0.117389798	7.425021078	0.026
A_51_P350214	Amz2	1.085446437	0.118288535	8.273693758	0.034
A_52_P89756	NA	1.08554957	0.118425606	6.09174633	0.049
A_52_P219314	Vasp	1.085979828	0.118997305	6.191494745	0.040
A_52_P502754	Ampd3	1.086106354	0.119165382	6.214847128	0.046
A_52_P406459	Ndufaf7	1.086550457	0.119755171	6.589461743	0.047
A_52_P209101	Abl1	1.086597171	0.119817196	6.150424942	0.037
A_52_P287917	Dcaf10	1.087726345	0.121315642	6.501916875	0.046
A_51_P497463	Dedd	1.08775234	0.12135012	6.538923458	0.044
A_51_P249824	Smap1	1.088406698	0.122217741	7.641717001	0.032
A_51_P294169	Cdc40	1.089169158	0.123228035	6.353890149	0.026
A_51_P432764	Sirpa	1.089234569	0.123314674	6.935133305	0.039
A_51_P343556	Cdv3	1.089254257	0.123340751	8.56353141	0.048
A_51_P197321	Clta	1.089952507	0.124265273	11.71983554	0.040
A_51_P471219	6430571L13Rik	1.090323421	0.124756143	6.156327161	0.026
A_51_P307316	Syde1	1.090787119	0.125369569	7.674658375	0.030
A_51_P362013	Rexo1	1.091086186	0.125765066	6.136942913	0.025
A_51_P277416	Fam3c	1.091569808	0.126404397	6.653816368	0.028
A_52_P605455	Eef2	1.091708052	0.126587098	6.280930294	0.031
A_52_P331883	Gng7	1.091750158	0.12664274	6.620249487	0.022
A_51_P217227	NA	1.091866751	0.126796803	9.063525026	0.038
A_52_P182659	Cs	1.09223779	0.127286979	11.82514444	0.048
A_51_P458852	NA	1.092608686	0.127776797	7.708528948	0.033
A_52_P363452	NA	1.09311703	0.128447865	6.148117448	0.037
A_52_P63343	Gm129	1.093885084	0.129461187	6.396783076	0.035
A_52_P403764	4930402H24Rik	1.093987125	0.12959576	6.274451465	0.030
A_51_P299339	Klf15	1.094648289	0.130467406	6.189070596	0.016
A_52_P402786	Prom1	1.095459724	0.131536442	6.231700032	0.037
A_51_P450459	NA	1.096293556	0.132634163	6.22587471	0.046
A_52_P350519	H2-Bl	1.096546899	0.132967518	6.204115859	0.031
A_52_P499980	Pias3	1.09717176	0.133789395	6.634089328	0.047
A_52_P449130	Ppfia1	1.097293269	0.133949161	6.306298111	0.050

A_51_P485688	Cdh6	1.097309555	0.133970572	6.120553931	0.018
A_51_P348636	Osbp2	1.097425947	0.134123591	6.981990127	0.027
A_52_P592639	Gabbr1	1.097844674	0.134673953	6.567879722	0.047
A_52_P141715	Dtna	1.099238147	0.136503976	6.852638435	0.040
A_52_P56658	Emc3	1.09925861	0.136530833	8.875616115	0.047
A_51_P233825	Akap1	1.099660991	0.137058831	6.133542574	0.032
A_51_P157554	Brd7	1.099893325	0.137363609	7.152975938	0.016
A_51_P488383	NA	1.100110087	0.1376479	6.341436564	0.015
A_52_P229709	Ube2d3	1.10043719	0.138076802	7.1318966	0.036
A_51_P240864	Sppl3	1.10085857	0.138629135	11.29138574	0.028
A_52_P240470	Ap1ar	1.10153889	0.13952043	6.65183317	0.040
A_51_P503542	Zfp414	1.10185091	0.139929027	8.310225182	0.029
A_51_P141860	Fbxw11	1.101948482	0.140056777	10.65785558	0.024
A_51_P231587	NA	1.102257452	0.140461231	8.059958558	0.025
A_51_P484671	Adcy3	1.102810019	0.14118428	6.411605001	0.043
A_51_P244543	2610507B11Rik	1.102973418	0.141398022	6.545891297	0.019
A_51_P174396	Aifm1	1.103033389	0.141476462	6.666217725	0.038
A_51_P268439	Mcam	1.103476698	0.142056165	8.889993641	0.044
A_51_P360622	Elmod3	1.104457336	0.14333769	7.301709316	0.033
A_52_P205991	Gnb1	1.104753611	0.143724647	7.833774244	0.034
A_51_P518298	Rnf187	1.104872083	0.143879351	11.29893997	0.015
A_52_P518014	Snrpa	1.105127435	0.14421274	6.506555053	0.036
A_51_P148093	Ptprm	1.105345654	0.144497587	6.771787448	0.040
A_51_P200291	Golga3	1.105408243	0.144579276	7.028348125	0.040
A_52_P442414	Dclk3	1.105456029	0.144641642	6.439849389	0.023
A_52_P397012	Tars	1.10547652	0.144668383	6.849314237	0.020
A_52_P413161	Cdyl2	1.105830109	0.145129758	6.520071169	0.014
A_52_P481793	Snx2	1.106009436	0.145363695	6.476206454	0.048
A_51_P138548	Hmgb3	1.106067611	0.145439577	9.536955256	0.020
A_52_P11441	Rab6b	1.106139542	0.145533396	12.48482909	0.045
A_52_P654021	Tex261	1.106362187	0.145823755	6.996266347	0.040
A_52_P539161	Rdh11	1.106377046	0.145843131	7.77415424	0.024
A_51_P435239	Asap1	1.106517438	0.146026188	7.949961165	0.016
A_52_P571591	Ash2l	1.106768984	0.146354119	7.049520068	0.023
A_52_P650325	Slc35e1	1.106840356	0.146447151	6.887779643	0.042
A_51_P160576	Brsk2	1.107190206	0.146903087	10.39163179	0.024
A_52_P189888	Dennd4b	1.107515331	0.14732667	6.271937808	0.018
A_51_P345362	Arhgef11	1.107661934	0.147517628	7.198399673	0.038
A_52_P243599	Hsd17b12	1.107836178	0.147744557	8.612384619	0.046
A_52_P514336	Trappc11	1.10792611	0.147861669	6.306906723	0.020
A_51_P496054	Zcchc14	1.108056922	0.148031996	6.836938856	0.048
A_52_P228398	Eif4h	1.108246844	0.148279255	11.9078686	0.030
A_52_P269384	1110018G07Rik	1.108641223	0.148792559	6.949800375	0.026
A_51_P205968	Snx15	1.10872578	0.148902589	8.114142337	0.042
A_51_P152685	Pcnxl2	1.10908757	0.149373281	10.50605021	0.032

A_51_P169087	Gls2	1.109368847	0.149739118	9.441828051	0.049
A_52_P647919	Usf2	1.10943272	0.14982218	7.310735654	0.027
A_51_P124505	Iars	1.109834577	0.150344656	6.983598517	0.026
A_51_P407193	Clp1	1.109876129	0.15039867	6.61868473	0.031
A_52_P235108	Vps35	1.109903125	0.15043376	6.363927004	0.027
A_52_P362670	Adam15	1.110114247	0.150708159	6.480436232	0.036
A_51_P348804	Amotl2	1.11058853	0.151324401	6.920160155	0.019
A_51_P418375	Jam2	1.110610412	0.151352827	8.339137139	0.031
A_51_P502964	Tmem63a	1.110846814	0.151659883	9.860320801	0.037
A_52_P213402	Dhx34	1.110900118	0.151729108	7.004367153	0.050
A_52_P556111	4933426M11Rik	1.11119915	0.1521174	6.874908138	0.028
A_52_P554267	Ilf3	1.111276368	0.152217652	8.908769749	0.029
A_52_P550932	H1f0	1.111319962	0.152274245	6.33807284	0.035
A_52_P375598	Rai1	1.111371596	0.152341275	7.532924715	0.048
A_51_P322871	Sh3bp4	1.111601282	0.152639404	6.486139611	0.038
A_52_P618173	Limch1	1.111645973	0.152697404	6.72453328	0.027
A_52_P496935	Armc9	1.111757431	0.152842048	6.123551371	0.032
A_51_P362104	Enpp5	1.111985129	0.153137494	9.443317416	0.016
A_51_P246705	Nop14	1.112068175	0.153245235	7.760103674	0.030
A_52_P637440	Hnrnpa1	1.112350055	0.153610873	6.470506877	0.023
A_51_P112817	Cyp27a1	1.112476943	0.153775435	6.595949261	0.014
A_52_P642879	Fbxo21	1.113100585	0.154583968	6.474777717	0.020
A_51_P235311	Rpl13a	1.113422914	0.155001679	11.32338739	0.033
A_51_P345714	Tsc2	1.113495066	0.155095166	9.851366432	0.015
A_52_P617963	Fbxl5	1.113593869	0.155223173	7.451766848	0.033
A_51_P120201	Eif5a	1.113666874	0.15531775	9.7935341	0.037
A_52_P141662	Tcf3	1.114212837	0.156024842	6.306202024	0.011
A_51_P177897	Ube2i	1.114220123	0.156034276	8.704414861	0.035
A_52_P423357	Yipf3	1.114225672	0.156041461	7.307855458	0.034
A_52_P477369	Csnk1d	1.115217081	0.157324564	6.479358252	0.020
A_51_P110395	NA	1.115756156	0.158021766	8.722675181	0.048
A_52_P626772	Ilf3	1.115963052	0.158289262	6.268754905	0.042
A_51_P395753	Chp1	1.116002018	0.158339636	6.508111456	0.040
A_51_P135137	St3gal4	1.116069605	0.158427005	6.339179185	0.014
A_51_P440242	Pogk	1.116072262	0.15843044	6.848786323	0.050
A_52_P479179	Cnot8	1.116195941	0.158590306	6.520145163	0.017
A_52_P577329	Tmem88b	1.116294275	0.158717398	7.211900491	0.044
A_52_P262275	Atxn2	1.116475256	0.158951278	6.487777211	0.036
A_51_P510817	Camk2d	1.11683154	0.159411589	7.710745461	0.022
A_51_P296100	Rhot2	1.116977524	0.159600156	10.31023738	0.024
A_52_P455494	M6pr	1.117470707	0.160237013	9.777131018	0.014
A_51_P148122	Klhdc3	1.11794938	0.160854866	10.37061138	0.016
A_52_P584293	Atrnl1	1.118031165	0.160960403	7.517652684	0.039
A_51_P444264	Rtn1	1.118483243	0.161543642	12.77543104	0.036
A_51_P342805	Ptpru	1.118611046	0.161708482	6.490887082	0.046

A_51_P186469	Sik3	1.119177248	0.162438539	9.426800784	0.031
A_51_P191601	2510039O18Rik	1.119429766	0.162764015	8.968631457	0.017
A_51_P268186	8430419L09Rik	1.119601794	0.162985704	6.429217002	0.025
A_51_P201609	Rnf26	1.119718136	0.163135612	9.328969929	0.019
A_51_P480290	Casz1	1.119840491	0.163293251	6.702098702	0.047
A_51_P394244	NA	1.120201025	0.163757653	8.129164533	0.036
A_52_P119393	Mta3	1.120278683	0.163857665	7.691457466	0.031
A_52_P684037	Ncam1	1.120311557	0.163899999	6.900869778	0.027
A_52_P108845	NA	1.121459483	0.165377498	10.59249146	0.035
A_51_P211980	Rgs3	1.121768955	0.165775563	7.818307708	0.049
A_52_P649170	Lonp2	1.122376423	0.166556608	6.703129849	0.024
A_52_P359381	Ptk2	1.122448682	0.166649487	6.576183429	0.007
A_51_P492676	Sardh	1.122887646	0.167213582	7.271018883	0.020
A_51_P183853	Polr2m	1.12293452	0.167273804	11.39948734	0.022
A_51_P156434	Slc25a33	1.123132817	0.167528545	8.169457531	0.038
A_51_P106373	Sdhc	1.123250615	0.167679852	8.833325301	0.046
A_51_P443723	Slc35c1	1.123267718	0.167701818	8.922965361	0.042
A_51_P406346	Magi1	1.12343703	0.167919262	9.24735644	0.049
A_52_P396884	2010300C02Rik	1.123577869	0.168100113	6.617044305	0.033
A_51_P155675	Sel1l	1.123846049	0.168444421	6.773810229	0.016
A_51_P504114	Atp11a	1.123928502	0.168550262	6.699161635	0.012
A_51_P466371	Pitpna	1.123992925	0.168632954	11.31361445	0.020
A_51_P511112	H1f0	1.12414548	0.168828753	6.129136432	0.007
A_52_P226407	NA	1.124190678	0.168886757	7.523447234	0.030
A_52_P48398	Rnf41	1.124387783	0.169139683	6.703316546	0.029
A_52_P350477	Mcfd2	1.124454791	0.169225658	6.478067284	0.033
A_51_P114049	Tmem109	1.124887031	0.169780123	10.47014261	0.007
A_51_P308557	Ncln	1.125174941	0.170149328	6.799718438	0.032
A_52_P231691	Wnt7b	1.125271429	0.170273039	7.096599672	0.039
A_51_P209225	Tada3	1.125294695	0.170302868	7.316398415	0.038
A_52_P652212	Psmd14	1.12551193	0.170581348	9.217721691	0.037
A_52_P686136	Prrc2b	1.125598596	0.170692434	11.14412105	0.032
A_52_P517247	Iqsec3	1.125987408	0.171190694	10.69669669	0.020
A_51_P224564	Ppm1f	1.1262665	0.171548242	10.40960359	0.044
A_52_P166952	Ppp2r5c	1.126394482	0.171712172	7.211759286	0.015
A_52_P676956	Tirap	1.126692084	0.172093292	6.634590226	0.048
A_52_P268104	NA	1.126721864	0.172131425	11.58491122	0.048
A_52_P235631	Herc2	1.126797792	0.172228642	7.196672255	0.021
A_51_P223776	Nr1d1	1.126809581	0.172243736	6.762205871	0.044
A_52_P925246	NA	1.126822118	0.172259787	6.58284719	0.014
A_51_P432420	Azi1	1.127016193	0.172508244	8.70048172	0.049
A_51_P491470	Ddx47	1.12705668	0.172560071	7.828382946	0.017
A_52_P404403	Xrn2	1.127086314	0.172598004	6.693411198	0.018
A_51_P219918	Tmem125	1.127219954	0.172769055	9.930818426	0.027
A_52_P427024	Ldlr	1.12767559	0.173352092	9.818374578	0.018

A_52_P45606	2510003E04Rik	1.127775198	0.173479521	7.121195521	0.021
A_52_P555688	Trappc12	1.127962933	0.173719658	7.697783533	0.047
A_52_P261322	Tanc1	1.128613811	0.174551909	6.322691302	0.013
A_51_P282630	Pacsin3	1.128969963	0.175007103	10.7330216	0.014
A_51_P427934	NA	1.129007659	0.175055274	9.220337571	0.028
A_52_P442234	H1f0	1.129056312	0.175117443	7.303514424	0.046
A_52_P677822	Tmem5	1.12906582	0.175129592	7.12998297	0.035
A_52_P314548	Zbtb9	1.129364531	0.175511228	6.293921069	0.011
A_52_P589065	Mbtps1	1.129643135	0.175867084	6.948566522	0.037
A_52_P566406	Xpc	1.130073372	0.176416446	6.971880299	0.019
A_51_P177984	Itfg3	1.130116017	0.176470886	8.959151932	0.038
A_52_P390241	Daam2	1.130196612	0.17657377	7.288839547	0.040
A_52_P588539	Snapin	1.130364456	0.176788006	7.286397584	0.050
A_52_P666930	Thra	1.130442779	0.176887967	8.498349989	0.015
A_52_P264902	Dnajc3	1.130447379	0.176893838	7.337873783	0.015
A_52_P42976	Gorasp2	1.130570307	0.177050712	6.638937863	0.040
A_52_P582424	9130221H12Rik	1.13075344	0.177284385	6.610378957	0.022
A_51_P269375	Ank1	1.130754168	0.177285314	10.95866071	0.008
A_52_P335606	Prima1	1.131085366	0.177707818	6.241020031	0.041
A_51_P462533	Syt7	1.131330461	0.178020402	9.38730707	0.010
A_51_P295708	Ints1	1.131563156	0.178317109	8.55109635	0.010
A_52_P18299	Chd5	1.131913006	0.178763083	8.340713815	0.027
A_52_P173197	Dusp7	1.131953819	0.178815101	9.693120086	0.032
A_52_P317040	Edem2	1.132295456	0.179250457	6.687256878	0.014
A_51_P371311	Slc1a4	1.132300827	0.1792573	6.955530778	0.007
A_52_P600038	Dlgap4	1.132433495	0.179426327	7.394538009	0.049
A_51_P277270	Rbmx	1.132581227	0.179614522	7.232181018	0.041
A_52_P587441	Ctnnd2	1.13270744	0.179775284	6.44341534	0.005
A_51_P126327	Otud7a	1.132770215	0.179855237	9.267535274	0.012
A_51_P506093	Clip3	1.132914454	0.180038928	7.577588645	0.043
A_52_P89425	Pcnt	1.133698829	0.181037434	8.184797144	0.020
A_52_P837662	2310057M21Rik	1.13388238	0.181270994	7.710400845	0.045
A_51_P236287	Safb	1.134142042	0.181601338	7.292491909	0.013
A_52_P393056	Wdr77	1.134795008	0.182431709	6.669209389	0.017
A_51_P333869	Ndnl2	1.134926484	0.182598848	7.35824496	0.045
A_51_P115027	Afg3l2	1.135108892	0.182830704	7.330057553	0.022
A_52_P291924	Ncam1	1.135201607	0.182948536	6.762130937	0.031
A_52_P85403	Hexim1	1.135234144	0.182989886	9.670585563	0.041
A_51_P167452	Snap47	1.135688603	0.183567314	12.95830835	0.010
A_52_P461292	Oaz1	1.135721647	0.18360929	11.00529986	0.031
A_51_P495641	Stmn1	1.135953092	0.183903262	13.41739608	0.049
A_51_P185259	Gnb5	1.136004849	0.183968992	7.637153097	0.024
A_52_P101184	Fbxw7	1.136196511	0.184212378	7.370155619	0.045
A_52_P654130	Oaz2	1.136492068	0.184587616	10.38852691	0.028
A_51_P426739	Gpt	1.136676579	0.184821819	8.102788065	0.037

A_52_P371946	Eif6	1.136708609	0.184862472	7.492676483	0.048
A_52_P438188	Itsn1	1.137100353	0.185359583	7.745986044	0.027
A_51_P360836	Txnl4a	1.137222526	0.185514582	10.78216018	0.049
A_52_P43111	Rnf7	1.137954042	0.186442293	9.731879819	0.029
A_51_P166394	Ap1s1	1.138087258	0.186611175	11.99170496	0.032
A_51_P505472	Ece2	1.138267749	0.186839956	6.907214197	0.028
A_52_P121960	NA	1.138672457	0.187352811	12.10637858	0.019
A_51_P161429	Snx11	1.138844118	0.187570288	6.644501191	0.034
A_52_P571707	Extl3	1.138877958	0.187613156	9.260331961	0.018
A_52_P421417	Vps36	1.139058052	0.187841276	7.192359292	0.042
A_51_P114062	Ncs1	1.139124504	0.18792544	9.920641759	0.008
A_52_P180972	NA	1.139167978	0.187980498	11.67912301	0.034
A_51_P177819	Rnf114	1.139620856	0.188553929	7.117737618	0.043
A_52_P656434	Dirc2	1.139735388	0.188698913	7.349022929	0.045
A_52_P271910	Pip5k1c	1.139789408	0.188767291	7.158870465	0.008
A_52_P331523	Brd9	1.139813319	0.188797557	7.134700477	0.049
A_51_P180629	Cdc42ep1	1.140070799	0.18912342	7.241301633	0.049
A_52_P256569	Dbndd2	1.140115961	0.189180568	10.20304748	0.019
A_51_P466221	Amhr2	1.140586443	0.18977579	6.875807386	0.028
A_51_P372418	Zfp706	1.140776466	0.190016124	9.818627116	0.014
A_52_P409833	Plat	1.140871487	0.190136289	7.356037008	0.047
A_52_P628455	Ewsr1	1.140876203	0.190142253	6.704731354	0.044
A_52_P445969	2810407C02Rik	1.140942489	0.190226073	10.43367832	0.045
A_52_P261562	Cpsf3	1.141121311	0.19045217	7.986718982	0.048
A_52_P277854	Snrpb	1.141151214	0.190489976	7.216260222	0.046
A_51_P329413	Pomgnt1	1.141347816	0.190738508	7.965141203	0.043
A_52_P614762	Bad	1.141711315	0.191197907	6.192458963	0.015
A_51_P164495	Dda1	1.141738561	0.191232335	8.561897087	0.037
A_51_P259555	Gpatch3	1.141772981	0.191275828	6.77665869	0.039
A_52_P16877	Tmcc3	1.141791112	0.191298737	7.144883866	0.016
A_51_P223404	Plin3	1.141868248	0.191396198	9.887244318	0.050
A_52_P548940	Trim11	1.141963533	0.191516582	6.963976242	0.044
A_52_P244572	Map4	1.142101256	0.191690563	9.993405114	0.044
A_52_P572447	Agpat5	1.142380379	0.192043106	7.867659843	0.048
A_51_P372874	Dpf2	1.142503371	0.192198422	8.935103264	0.008
A_51_P504423	Cryab	1.142818573	0.192596389	8.151804827	0.036
A_51_P303675	Slc18a3	1.142911842	0.192714126	6.591296337	0.026
A_52_P323315	Tmem151a	1.143252714	0.193144345	11.19914198	0.037
A_51_P282297	Naa11	1.143271968	0.193168641	7.047316181	0.015
A_51_P186899	Egln1	1.143286816	0.193187378	6.50055583	0.013
A_51_P346704	Sox10	1.143867065	0.193919399	8.578256609	0.047
A_52_P436700	Gltp	1.143975423	0.194056057	7.606117421	0.046
A_52_P572178	D130043K22Rik	1.144040615	0.194138271	7.351021421	0.045
A_52_P79038	Scaf1	1.144323212	0.194494596	6.87840611	0.029
A_51_P275679	Rassf5	1.144456545	0.194662685	6.896057887	0.022

A_51_P100034	Mif4gd	1.144525976	0.194750207	8.95934031	0.006
A_52_P360515	Celsr2	1.144941991	0.195274505	7.908645083	0.026
A_52_P515036	Htatip2	1.14515175	0.19553879	6.624820389	0.031
A_51_P391996	Pgd	1.145521342	0.196004337	7.271859204	0.039
A_51_P296292	Nono	1.145797402	0.196351971	9.912466055	0.038
A_52_P353038	Trim26	1.146197592	0.19685577	6.471670401	0.045
A_51_P404875	Synm	1.146566635	0.197320202	9.786758827	0.023
A_52_P258194	Crtac1	1.146598138	0.197359841	10.17267622	0.042
A_51_P470769	Pcdhga9	1.146657556	0.197434601	9.961377535	0.034
A_51_P271425	Lhfpl4	1.147008233	0.197875747	10.92328535	0.024
A_52_P143287	Itgb4	1.147053018	0.197932076	9.875413684	0.040
A_51_P108334	Slc25a4	1.147272572	0.198208192	11.83070248	0.017
A_51_P338615	Adprh	1.147599012	0.198618631	8.254766377	0.041
A_51_P140211	Ndufv3	1.147659596	0.198694791	7.374100818	0.042
A_51_P144926	Cops8	1.147793872	0.198863577	8.512223081	0.030
A_51_P218953	Zfp536	1.14781902	0.198895186	9.910549559	0.037
A_51_P288839	Otud5	1.147875531	0.198966213	7.856260159	0.012
A_52_P136808	Dtymk	1.147888565	0.198982594	8.04111123	0.019
A_51_P188845	Adora1	1.147960954	0.199073572	6.740744045	0.019
A_52_P366105	Tmem55b	1.148356521	0.199570613	8.346954629	0.035
A_51_P432544	H2-T22	1.14841811	0.199647987	6.949584692	0.047
A_51_P269652	Slc25a3	1.148565535	0.199833177	10.81387581	0.040
A_51_P136277	Csnk1d	1.148645828	0.199934028	7.062697445	0.012
A_51_P215496	NA	1.148738853	0.200050862	8.605412639	0.045
A_52_P194805	Zfp706	1.148746338	0.200060263	8.509043151	0.018
A_52_P226788	Rogdi	1.148753799	0.200069632	7.343010749	0.035
A_51_P335350	Ankrd40	1.148928474	0.200288987	8.603773449	0.017
A_52_P315988	Ccdc88c	1.14907418	0.200471936	6.442274385	0.004
A_52_P364279	Iqcc	1.149227628	0.200664581	6.383054557	0.009
A_51_P173858	Apba2	1.149426487	0.2009142	8.574137701	0.015
A_51_P249268	D130043K22Rik	1.149717238	0.201279087	6.842909551	0.045
A_52_P184304	Dst	1.150099423	0.201758584	6.647557678	0.011
A_51_P483658	Trim3	1.150563663	0.202340813	8.004135484	0.017
A_52_P524426	Epb4.1l1	1.150785121	0.202618473	9.517427496	0.047
A_51_P216905	Aldoa	1.151078803	0.202986604	13.60883259	0.045
A_52_P102207	NA	1.151087825	0.202997912	8.622140462	0.004
A_51_P363801	Pgpep1	1.151330355	0.20330185	7.155772531	0.032
A_51_P343818	Pgbd5	1.151374766	0.203357499	6.62358974	0.007
A_51_P326542	Dnaja3	1.151597266	0.203636269	6.8262623	0.023
A_52_P327381	Fndc4	1.15174337	0.203819293	8.169261385	0.033
A_51_P280404	Epdr1	1.151795597	0.203884712	8.938637198	0.050
A_51_P155152	Ank	1.151921868	0.204042866	9.564209864	0.042
A_51_P479769	Ampd2	1.151964083	0.204095735	9.844593756	0.031
A_51_P264634	Strbp	1.151980997	0.204116919	6.950691838	0.031
A_51_P269029	Rnf112	1.152021781	0.204167994	7.526855523	0.016

A_51_P402496	Atp5a1	1.152139969	0.204315995	11.59406405	0.020
A_51_P356355	Cds2	1.152291873	0.204506194	10.27745547	0.026
A_52_P263068	Rhog	1.152330554	0.204554623	8.702698507	0.040
A_51_P219532	Fbxo31	1.152917249	0.205288967	11.87921192	0.047
A_52_P274184	Vps39	1.153308293	0.205778213	6.4320126	0.024
A_52_P614582	Ube4b	1.153756593	0.206338892	7.423067056	0.029
A_52_P120424	Vcp	1.153815871	0.206413014	10.4380073	0.042
A_52_P594355	Tbc1d5	1.154055283	0.206712336	7.32816784	0.011
A_52_P339912	Inpp5j	1.154199941	0.206893162	6.867762269	0.042
A_51_P420415	Srd5a1	1.154618877	0.207416718	8.019237876	0.028
A_51_P501312	Gm16515	1.154732241	0.207558358	8.711800058	0.022
A_51_P187612	Desi1	1.154948121	0.207828049	7.85886328	0.006
A_51_P473252	Zyx	1.155265939	0.208224995	10.08816582	0.009
A_52_P27103	Necap2	1.155291939	0.208257463	6.959271343	0.008
A_51_P174864	Rnf41	1.15531415	0.208285198	6.539064286	0.034
A_51_P138348	Ap1b1	1.155365013	0.208348712	10.18117341	0.017
A_51_P180905	NA	1.155503854	0.208522071	6.330433802	0.026
A_51_P433194	Bcas1	1.155934413	0.209059543	12.62542534	0.043
A_51_P251508	Lemd2	1.156175562	0.209360484	9.006104886	0.031
A_51_P135423	Capzb	1.156401471	0.209642349	10.37898943	0.003
A_52_P22365	Cnot1	1.156604031	0.209895036	6.864153196	0.017
A_52_P167958	Gripap1	1.156708586	0.210025446	8.689413184	0.032
A_52_P535946	Dhcr7	1.156719432	0.210038974	6.527116071	0.021
A_52_P537492	Tmx2	1.157218465	0.210661248	8.082056169	0.049
A_51_P109171	Os9	1.157223161	0.210667103	9.844961723	0.038
A_52_P81980	Micu1	1.157237946	0.210685536	7.393089288	0.039
A_52_P247388	NA	1.157299311	0.210762035	6.774198775	0.003
A_52_P344376	Eif4a2	1.15873328	0.212548522	9.725902937	0.013
A_52_P377326	Plekhb2	1.158990436	0.212868662	6.647752387	0.027
A_52_P514306	Spata2	1.159125568	0.213036862	7.909660242	0.029
A_51_P209930	Rtn2	1.159143993	0.213059794	9.150625821	0.024
A_52_P16232	Gabbr1	1.159157824	0.213077008	10.35419058	0.025
A_51_P281806	NA	1.159503848	0.213507608	10.50966201	0.031
A_52_P120022	Prosapip1	1.159516993	0.213523963	8.535142898	0.015
A_51_P247665	Trappc12	1.159803845	0.213880826	7.866157705	0.026
A_52_P172619	Egln1	1.160007015	0.21413353	8.270687566	0.007
A_52_P614731	Gng12	1.16008	0.214224298	8.558604622	0.033
A_51_P126177	Map1lc3b	1.160273978	0.214465512	11.97701169	0.013
A_51_P116130	Ube2g2	1.160297037	0.214494184	6.46105368	0.036
A_52_P378719	Eftud1	1.160382164	0.214600026	6.57414248	0.007
A_52_P587606	2310022A10Rik	1.160747297	0.215053922	6.419593782	0.036
A_51_P358112	Fads1	1.16074957	0.215056747	9.650060902	0.048
A_51_P140311	Gnb2	1.161542939	0.216042488	10.07325782	0.045
A_52_P2659	NA	1.161672652	0.216203589	11.39361266	0.036
A_52_P213400	Dhx34	1.161911493	0.216500177	7.275215558	0.030

A_52_P442691	Mccc1	1.162517381	0.217252287	6.618932714	0.015
A_51_P141970	Mum1	1.162847012	0.217661303	6.554757411	0.002
A_52_P408530	NA	1.162892264	0.217717445	9.53581981	0.033
A_51_P427444	Snx3	1.162940921	0.217777808	9.489499025	0.041
A_52_P369310	Ogdh	1.163214755	0.218117475	7.923464769	0.042
A_52_P385801	Spock2	1.163526534	0.218504112	8.976149671	0.010
A_52_P123738	Rnf41	1.16404147	0.219142456	6.561025835	0.029
A_51_P186735	Tesk2	1.164059906	0.219165306	7.499355191	0.011
A_51_P393934	Cd82	1.164210206	0.21935157	9.127466741	0.020
A_52_P327537	Mpdz	1.164512869	0.219726583	6.979866224	0.022
A_52_P621940	Epb4.1l2	1.164591985	0.219824595	6.82990952	0.011
A_52_P589568	Foxo6	1.164812267	0.220097454	7.852263066	0.023
A_51_P189343	Map7d1	1.164996013	0.220325017	11.32642308	0.027
A_51_P223078	Gm10033	1.16541425	0.220842856	6.561932842	0.032
A_51_P219868	Dnm1	1.16560576	0.221079911	9.249443997	0.037
A_51_P427432	Grhpr	1.16585545	0.221388925	10.51848271	0.009
A_52_P18665	Dtnbp1	1.166664639	0.222389913	7.680849486	0.041
A_51_P259879	Fkrp	1.167140122	0.222977776	7.94598216	0.022
A_51_P153982	Specc1	1.16822951	0.224323734	10.5501296	0.045
A_51_P189927	Tm7sf3	1.168252222	0.224351781	6.509984939	0.021
A_51_P341789	Sugp1	1.168618018	0.224803439	9.990696018	0.030
A_51_P187171	Smim12	1.168707661	0.224914101	10.68498759	0.031
A_51_P442402	Mgrn1	1.168955709	0.225220268	11.75227772	0.031
A_52_P600087	NA	1.169138754	0.22544616	11.95704955	0.045
A_51_P371993	Tmed10	1.169156799	0.225468426	11.10888508	0.008
A_52_P268880	Rell2	1.169587185	0.22599941	8.394838257	0.025
A_51_P191669	Chgb	1.170495788	0.227119742	12.97564793	0.018
A_51_P307721	Cbln1	1.17094165	0.227669185	9.574210045	0.044
A_51_P291224	Mobp	1.170964436	0.227697259	9.192101758	0.021
A_51_P359813	Csnk1d	1.170964824	0.227697738	10.82444928	0.030
A_51_P192501	Gramd3	1.171198817	0.227986001	7.558398466	0.018
A_51_P248786	Ccdc80	1.171806733	0.228734645	7.765732793	0.049
A_52_P229648	Pacsin2	1.172507272	0.22959687	6.738333396	0.013
A_52_P504236	Slc20a2	1.172690931	0.229822834	6.685728646	0.035
A_51_P174906	Prpsap1	1.173691341	0.231053056	8.625454259	0.005
A_51_P416046	Trim41	1.173914752	0.231327646	7.624093362	0.007
A_52_P266540	Ubr4	1.175024654	0.232691027	9.548505104	0.038
A_51_P178063	Rasa3	1.175391613	0.233141509	9.764417094	0.010
A_51_P321126	Fasn	1.176418367	0.234401214	12.25697324	0.003
A_52_P272811	Cpsf1	1.176648447	0.234683344	8.204123732	0.040
A_52_P495318	Tomm40	1.176854446	0.234935898	6.800005275	0.025
A_51_P447976	Fam46c	1.176892778	0.234982888	6.487763539	0.012
A_52_P58041	Arpc5	1.177003382	0.235118466	7.370453744	0.016
A_51_P238523	Shisa4	1.177441681	0.235655605	9.931990845	0.004
A_52_P123485	Tcf25	1.17809151	0.236451607	12.93124646	0.009

A_52_P473172	NA	1.178144529	0.236516533	9.706228991	0.008
A_51_P202801	Abcb9	1.178382329	0.2368077	10.82469927	0.003
A_51_P439612	Dnajb2	1.178931655	0.237480085	10.51858514	0.012
A_51_P365008	NA	1.179349061	0.237990787	9.282875772	0.008
A_52_P282279	Mthfd1	1.179450494	0.238114865	7.025558121	0.037
A_52_P308465	Plxnb1	1.179638727	0.238345092	7.825737359	0.047
A_52_P193925	Sulf2	1.180012991	0.238802743	9.264855479	0.022
A_52_P138727	Sirt7	1.180382397	0.239254311	8.068354329	0.050
A_51_P318580	Myh14	1.180427424	0.239309343	10.46645294	0.027
A_51_P460633	Hgs	1.180952479	0.239950912	7.42917388	0.017
A_52_P37077	Ncam1	1.181223706	0.240282215	6.926690513	0.026
A_52_P97889	B4galnt4	1.181658506	0.240813164	7.211216042	0.017
A_51_P384994	Grik4	1.182346897	0.24165338	9.131749967	0.008
A_51_P475628	Paqr6	1.182568735	0.24192404	9.600971094	0.005
A_51_P356705	Plekhb2	1.182721853	0.242110827	10.26338483	0.008
A_51_P154222	Kars	1.183327385	0.242849272	8.971150015	0.014
A_52_P320032	Fus	1.183753797	0.243369054	7.70706229	0.024
A_52_P674530	Hk1	1.184917257	0.244786319	7.405528637	0.027
A_51_P433733	Nucb1	1.185044214	0.244940887	8.825085947	0.022
A_52_P262080	Snurf	1.185310206	0.245264674	8.518779484	0.038
A_51_P262340	Rbm3	1.185739336	0.245786894	9.629994897	0.015
A_52_P352131	Acvr1	1.185771321	0.24582581	7.270621908	0.011
A_52_P171064	Wnk1	1.186821419	0.247102869	8.886687175	0.034
A_52_P568257	Sort1	1.18709464	0.247434957	8.535318405	0.021
A_51_P300717	Stxbp1	1.187312096	0.24769921	11.36644989	0.016
A_51_P144438	Znfx1	1.187374305	0.247774799	7.611579359	0.050
A_52_P290369	Usp19	1.18754489	0.247982049	7.184651832	0.039
A_51_P239766	Plcd1	1.18754758	0.247985317	8.39796341	0.024
A_51_P358940	Wbp2	1.187891358	0.248402897	10.56860371	0.017
A_52_P522097	Adipor1	1.187938467	0.24846011	7.53818378	0.014
A_51_P411645	Maea	1.188063598	0.248612066	7.825473774	0.036
A_51_P114094	Clstn3	1.188496022	0.249137075	11.95771567	0.044
A_51_P311945	Oaz2	1.189520709	0.250380389	10.03134506	0.031
A_51_P323878	Coro7	1.190115309	0.251101361	9.650991063	0.027
A_51_P490023	Tubb2a	1.190121241	0.251108553	13.28086786	0.031
A_52_P166694	Vamp1	1.190158648	0.251153898	12.04102212	0.050
A_52_P377160	Galnt6	1.190285178	0.251307268	6.975783599	0.001
A_52_P419298	Lasp1	1.191862874	0.253218261	7.058144556	0.004
A_52_P508985	Asb8	1.192485512	0.253971738	7.65121114	0.046
A_52_P485971	Scap	1.19260325	0.254114174	10.58903886	0.004
A_51_P360840	Zfpm1	1.192650233	0.254171008	9.064252842	0.005
A_52_P510647	Chtop	1.193395006	0.255071644	9.595897448	0.006
A_51_P125695	Scn8a	1.193474393	0.255167613	7.646627665	0.038
A_52_P86176	Tap2	1.193757882	0.255510259	6.732532076	0.009
A_51_P170371	Hspa8	1.193769096	0.255523811	13.27071209	0.015

A_51_P142057	Ap2a1	1.193884444	0.255663205	7.56138107	0.009
A_52_P305230	Igsf21	1.194323925	0.256194178	9.678978126	0.016
A_51_P409919	Emc1	1.194704735	0.256654108	6.682439457	0.040
A_52_P633597	Rftn1	1.194929822	0.256925892	7.438238923	0.005
A_52_P566718	Acss2	1.195120409	0.257155978	6.943049897	0.030
A_51_P185794	Preb	1.195204564	0.257257562	9.921623988	0.050
A_51_P386638	Llgl1	1.195292201	0.257363343	9.675677948	0.038
A_51_P175018	Apcdd1	1.195686778	0.257839511	8.896068616	0.021
A_52_P642109	Prpsap1	1.19582376	0.258004782	8.511915374	0.035
A_51_P514922	2610301G19Rik	1.195910814	0.258109804	7.658158524	0.009
A_52_P683572	Wsb2	1.196286646	0.258563119	7.738695893	0.024
A_52_P623337	Ncl	1.196991967	0.259413471	11.71539374	0.044
A_51_P512364	Fus	1.197047455	0.259480347	9.425942352	0.039
A_51_P381683	Aatk	1.19719632	0.259659749	12.48530965	0.004
A_52_P656024	Sirt2	1.197713976	0.260283422	8.600995586	0.017
A_52_P282500	Kif21b	1.197941894	0.260557933	9.047152332	0.003
A_51_P180492	Dbp	1.19841199	0.261123963	11.46820467	0.025
A_52_P113916	Gm7146	1.199025785	0.261862684	6.216218934	0.016
A_52_P376574	NA	1.199169262	0.262035309	9.395786471	0.033
A_51_P348372	Prex1	1.200045938	0.263089634	8.226579559	0.034
A_52_P460526	Eif4g2	1.200164352	0.263231984	8.375493514	0.038
A_52_P279759	Glg1	1.200552674	0.263698703	7.278929146	0.045
A_51_P464300	Gdf1	1.200632835	0.263795028	11.83578637	0.006
A_52_P90289	Oaz2	1.201694236	0.265069857	7.975847373	0.024
A_52_P322389	Strn4	1.201765575	0.265155501	6.6733188	0.005
A_52_P178998	Fam168b	1.201795104	0.26519095	10.82529848	0.010
A_52_P27871	Fnbp1	1.20218207	0.265655408	7.8205401	0.028
A_52_P578562	Slc41a1	1.202721303	0.266302377	7.939631692	0.024
A_52_P268206	Mcam	1.202806657	0.266404758	6.563271983	0.001
A_51_P168862	Snrpn	1.203024409	0.266665915	9.235505107	0.003
A_51_P260051	Arf1	1.203547747	0.267293378	8.554523242	0.009
A_51_P343566	Galnt10	1.203566135	0.26731542	7.994829494	0.034
A_52_P28651	Pvrl1	1.203985557	0.267818086	8.564524924	0.022
A_52_P354390	Snrpn	1.204457079	0.268382983	9.855355693	0.044
A_52_P473813	NA	1.20485294	0.268857068	9.594001953	0.016
A_51_P234833	Strn4	1.204865854	0.26887253	8.409276419	0.003
A_51_P263503	Mapk1	1.205373581	0.26948035	6.852293435	0.003
A_52_P642662	Efhd1	1.20576984	0.269954548	6.679781262	0.013
A_51_P519756	Rusc1	1.205866934	0.270070717	10.3933183	0.029
A_51_P488399	Acss2	1.205888269	0.270096241	8.305337327	0.034
A_52_P177847	Tril	1.205999257	0.270229018	7.667070214	0.014
A_52_P157170	Rnf157	1.206505114	0.270834031	9.106858014	0.032
A_52_P140072	Dlst	1.206883693	0.271286651	7.582395986	0.002
A_51_P259603	Adcyap1r1	1.207200701	0.271665549	9.902023504	0.040
A_52_P189235	Dnajc27	1.207386857	0.271888002	7.693184741	0.050

A_51_P354272	Cluh	1.207458754	0.271973908	9.834236504	0.015
A_52_P461378	Tmbim1	1.207690526	0.272250807	7.646820215	0.022
A_52_P675996	Klf9	1.208021776	0.272646461	8.223921774	0.013
A_52_P383753	Tom1l2	1.208190831	0.272848344	10.41172448	0.050
A_51_P425749	Cdc37	1.20868497	0.273438272	7.759264919	0.026
A_52_P179272	Usp30	1.209049054	0.273872779	6.622779407	0.013
A_52_P772918	Larp1	1.211402765	0.276678609	6.868305127	0.015
A_51_P510567	Zfyve28	1.211464196	0.276751767	7.469057731	0.002
A_51_P483946	Dmwd	1.211718284	0.277054321	8.508718296	0.038
A_51_P499061	Ube2o	1.212186712	0.277611933	11.61909614	0.005
A_52_P568028	Ncdn	1.212755607	0.278288849	9.653465164	0.010
A_51_P442097	Slc41a3	1.213450219	0.279114924	7.451172346	0.004
A_51_P507942	Atp13a2	1.21412486	0.279916795	11.28578781	0.005
A_52_P143866	Glul	1.215082656	0.281054456	7.364518328	0.005
A_52_P463143	Cdc37	1.215462807	0.281505747	11.87296318	0.028
A_51_P439746	Vmp1	1.217260115	0.283637489	8.974874002	0.027
A_52_P193611	Pkd2l1	1.217637538	0.284084741	6.6836589	0.045
A_52_P670978	Nkain1	1.218025398	0.284544216	7.933324414	0.037
A_52_P676744	St6galnac6	1.219102495	0.285819425	8.734591851	0.025
A_51_P133229	Sulf2	1.219507717	0.286298887	9.383014718	0.025
A_51_P193176	Slc25a25	1.219864144	0.286720484	8.514670413	0.016
A_51_P169061	Lpcat2	1.220242465	0.287167843	7.939793697	0.005
A_52_P557129	Slc12a5	1.220567605	0.287552206	13.40589404	0.014
A_52_P661327	Phyhipl	1.221339768	0.288464604	7.192995855	0.003
A_52_P347176	Nat8l	1.222032709	0.289282901	12.79350354	0.004
A_52_P116384	Usp4	1.222292829	0.289589958	8.185939106	0.020
A_52_P467726	Nsg1	1.222459168	0.289786278	11.95523024	0.016
A_52_P650855	Myo1d	1.223499869	0.291013947	10.3228362	0.047
A_52_P352187	Acsl6	1.224396755	0.292071126	6.835289552	0.026
A_52_P604618	Sbf1	1.224765848	0.29250596	7.227562051	0.013
A_51_P194249	Stmn4	1.226381746	0.294408128	13.90919597	0.021
A_52_P350664	Pygb	1.226771478	0.29486653	9.771442267	0.048
A_51_P216742	Arhgap23	1.226871486	0.294984135	8.859622731	0.023
A_51_P161582	Ddr1	1.227486129	0.295706721	10.58632352	0.005
A_52_P282741	Sdc3	1.227868603	0.296156183	8.396446058	0.039
A_52_P269003	Neo1	1.227980434	0.296287574	7.525883511	0.003
A_51_P130459	Vdac1	1.228468092	0.296860386	9.995869047	0.001
A_51_P257640	NA	1.228963916	0.297442557	7.39821965	0.010
A_52_P429909	Dynll2	1.230629167	0.299396092	11.80778112	0.033
A_51_P244453	Kctd3	1.231173965	0.300034629	8.21738305	0.039
A_52_P109503	Sdha	1.232038494	0.301047332	9.274912563	0.005
A_51_P411007	Cdk2ap1	1.232267175	0.301315089	9.132643702	0.028
A_52_P546135	NA	1.232448297	0.301527125	7.119644343	0.002
A_52_P54976	NA	1.233358764	0.302592518	7.873469204	0.040
A_51_P124285	Nkd1	1.233813554	0.3031244	8.25781992	0.002

A_51_P362638	Trf	1.234029251	0.303376592	12.34321398	0.041
A_52_P485007	Abca2	1.236514248	0.306278863	9.111611377	0.006
A_52_P518233	Ndrg3	1.236842263	0.306661523	7.615724541	0.010
A_52_P85040	Mog	1.237603509	0.307549193	8.034547364	0.010
A_51_P241465	Gsn	1.238331017	0.308397011	12.29743954	0.008
A_51_P363396	Klc2	1.238413863	0.308493526	6.757388615	0.023
A_51_P314277	Parp1	1.23886123	0.309014594	7.890391969	0.007
A_51_P397920	D17Wsu92e	1.239188233	0.30939535	9.458906024	0.021
A_51_P436719	Eftud2	1.239255786	0.309473994	8.323356826	0.030
A_51_P500135	Ndrg4	1.239725125	0.310020278	13.23465452	0.002
A_51_P228883	Htatip2	1.239869965	0.310188822	7.527049042	0.031
A_51_P506513	Qdpr	1.239995336	0.310334694	11.98704614	0.011
A_52_P376829	Rpl4	1.240261753	0.310644628	9.403469598	0.005
A_52_P276302	Tshz1	1.240802024	0.311272944	7.060904364	0.010
A_51_P433837	Slc22a23	1.241158431	0.311687284	9.104436035	0.019
A_52_P111715	Galnt6	1.242032952	0.31270345	8.127554755	0.007
A_51_P355416	Nisch	1.242802266	0.313596777	10.46741731	0.038
A_52_P674489	Atp5a1	1.243863999	0.314828754	12.35749123	0.042
A_52_P49378	Kif1a	1.244493399	0.315558578	12.55121955	0.002
A_51_P339200	Abhd16a	1.246131011	0.317455753	8.202939512	0.048
A_51_P169745	Tuba1a	1.246150027	0.317477768	13.31823311	0.010
A_52_P326214	Cttn	1.246940592	0.318392733	7.418090527	0.017
A_51_P195506	Csf1	1.247108029	0.318586442	8.18626987	0.041
A_52_P128068	Kazn	1.247463983	0.318998162	8.246019223	0.027
A_52_P229044	Slc20a2	1.247811359	0.319399847	6.917196796	0.010
A_51_P167313	Pgbd5	1.248742998	0.320476589	10.34015265	0.034
A_51_P128667	Lynx1	1.24878377	0.320523693	11.12867886	0.035
A_51_P394515	Tkt	1.250686481	0.322720183	12.00966603	0.023
A_52_P448205	Clstn1	1.25192978	0.324153645	12.78360005	0.005
A_51_P349341	Npc1	1.253028316	0.325419018	7.321206144	0.003
A_51_P160870	Rtn4	1.253202891	0.325620003	9.962956627	0.019
A_51_P274992	Gar1	1.253784536	0.326289441	6.90286089	0.039
A_51_P226645	Cacng2	1.255419053	0.328169009	8.176599432	0.022
A_52_P482849	NA	1.256753885	0.329702148	7.922307503	0.009
A_51_P215038	Tmem59l	1.257815518	0.33092034	11.68281033	0.006
A_51_P485810	Pygb	1.258611272	0.331832769	8.384300049	0.029
A_52_P436564	Cdh20	1.25925708	0.332572842	9.043535732	0.019
A_52_P379337	Rtn4	1.259532434	0.332888273	10.2254835	0.002
A_52_P381846	Rasa3	1.260706223	0.33423213	6.814348956	0.004
A_51_P450632	Usp5	1.261458155	0.335092351	8.061219565	0.026
A_52_P621603	Tubb2a	1.261566686	0.335216469	13.10356256	0.008
A_52_P581056	Tyro3	1.262261132	0.336010401	8.324602714	0.014
A_51_P483908	Dctn1	1.262960584	0.336809615	10.79503277	0.026
A_51_P204582	Rnf5	1.263130215	0.337003373	8.492084816	0.030
A_51_P199041	Adcy5	1.26337299	0.337280633	7.427299504	0.035

A_51_P509518	Ralgds	1.263839941	0.337813765	10.62683303	0.020
A_52_P428354	H2-Q10	1.264043747	0.338046394	6.629508471	0.003
A_51_P193011	Klc1	1.266229105	0.340538463	13.62181281	0.007
A_51_P260504	Arhgef4	1.269911775	0.344728272	11.48965422	0.025
A_51_P372141	Pnkd	1.271595893	0.346640262	8.046950437	0.022
A_51_P454949	Gstm3	1.272301135	0.347440176	10.68061004	0.027
A_51_P232901	Cnp	1.27307928	0.348322265	12.07838927	0.002
A_52_P57651	Rpn2	1.273354392	0.348633997	7.436950327	0.017
A_51_P102809	Gnl1	1.274397686	0.349815552	8.926087024	0.011
A_52_P449208	Adcy5	1.274438367	0.349861605	7.144834166	0.027
A_51_P356353	Cds2	1.275300655	0.350837405	12.88541095	0.003
A_52_P282035	Rnf5	1.276931002	0.352680572	8.97776296	0.013
A_51_P281835	Inf2	1.276945275	0.352696698	9.631683927	0.002
A_52_P12877	Hspa8	1.277097385	0.352868542	11.56003611	0.007
A_52_P495565	Efnb3	1.278952951	0.354963193	10.46919414	0.004
A_51_P172054	Gas6	1.279354045	0.355415567	11.47533906	0.008
A_51_P111902	Slc22a17	1.279824248	0.355945706	8.285987903	0.030
A_51_P515623	Qpctl	1.279982906	0.356124543	7.133447303	0.032
A_52_P112188	Gnas	1.280836389	0.357086201	9.600728705	0.019
A_52_P363920	Psmd2	1.281255326	0.357558002	9.51481009	0.003
A_52_P631591	Mast3	1.28339922	0.359970011	8.869353621	0.040
A_51_P222453	Tmem254a	1.284367249	0.361057782	7.67034161	0.021
A_51_P207636	Atp5b	1.285812607	0.362680402	11.5322491	0.021
A_52_P243102	Kctd3	1.28602174	0.362915031	6.986273665	0.032
A_52_P248378	Cry2	1.288840093	0.366073278	7.940210568	0.033
A_51_P246844	Abca2	1.288901858	0.366142415	8.880033547	0.023
A_51_P431870	Map1s	1.289392094	0.366691042	8.099589497	0.003
A_51_P386189	Tnk2	1.289416053	0.36671785	9.176925083	0.012
A_52_P310140	Wsb2	1.290351886	0.36776455	7.214393771	0.012
A_52_P665386	Ube2m	1.292776304	0.37047266	9.158852792	0.021
A_51_P100246	Ube2m	1.292878051	0.370586201	8.974821763	0.029
A_51_P171107	Tmem35	1.293064142	0.370793841	10.19535007	0.002
A_52_P174313	Aktip	1.293335266	0.371096308	8.392622697	0.006
A_52_P227267	Atp1a2	1.295273607	0.373256878	10.95347608	0.036
A_52_P57582	NA	1.296620984	0.374756827	8.453910905	0.044
A_52_P395397	Aars	1.297136195	0.375329966	9.016966086	0.020
A_51_P145220	Nefm	1.298328981	0.376655991	12.66448046	0.006
A_52_P429308	Efhd2	1.299353611	0.377794104	9.963824332	0.038
A_51_P346472	NA	1.300644888	0.379227121	7.833407	0.028
A_51_P303231	Gna12	1.300815786	0.37941667	6.993360794	0.001
A_52_P491766	Gnl1	1.301759261	0.380462671	9.11593299	0.021
A_52_P267256	NA	1.304847361	0.383881053	11.44321684	0.038
A_51_P454993	Tmcc2	1.31067628	0.390311403	8.280647195	0.000
A_51_P472726	Pdlim2	1.313924558	0.393882442	10.79348405	0.003
A_51_P200068	Arf3	1.31663006	0.396850042	9.761513394	0.028

A_51_P444565	NA	1.321580694	0.402264517	8.905914627	0.036
A_51_P162176	Trappc3	1.324411899	0.405351878	8.59937122	0.025
A_51_P401659	Sspn	1.324944936	0.405932404	8.6910528	0.016
A_51_P287986	Clstn1	1.32559924	0.40664468	7.609298857	0.012
A_52_P328867	Prkcb	1.327053711	0.408226763	8.311493695	0.003
A_52_P238556	Ubc	1.327358551	0.40855813	12.27636609	0.016
A_52_P320553	NA	1.327441829	0.408648641	10.1269749	0.002
A_52_P178470	Ndrg4	1.32849794	0.40979599	7.575482238	0.045
A_51_P155294	Ppp1r16b	1.33061642	0.412094742	7.606020267	0.020
A_52_P112182	Gnas	1.33406194	0.415825652	10.89271186	0.011
A_52_P205572	Sumo3	1.334394785	0.416185555	8.369830292	0.026
A_51_P385906	NA	1.335532949	0.417415569	9.624538468	0.002
A_51_P128148	Chmp1a	1.33563489	0.417525686	9.214055345	0.026
A_52_P72237	Actg1	1.337078197	0.419083842	10.48505671	0.000
A_51_P351872	Slc6a9	1.337921047	0.419992983	12.925014	0.000
A_52_P348627	Nbr1	1.342153203	0.42454936	7.421631826	0.003
A_51_P215627	Plac9a	1.349773887	0.432717749	7.717750776	0.049
A_52_P578266	Tank	1.351284924	0.434331905	6.280721719	0.040
A_52_P604849	Cspg5	1.352442274	0.435567017	8.603917292	0.002
A_51_P425048	H2-Q1	1.352703094	0.435845216	7.11192639	0.003
A_52_P81562	Eef2	1.358189996	0.441685311	11.16068292	0.003
A_51_P358755	Nsg1	1.358702296	0.442229383	9.045671646	0.006
A_51_P264388	Mapk8ip3	1.358717888	0.442245939	13.10375473	0.002
A_51_P433192	Bcas1	1.36128999	0.444974431	9.599218712	0.006
A_51_P456266	Tyro3	1.362512521	0.446269488	7.751403915	0.029
A_52_P424767	Rbbp4	1.366599937	0.450590965	6.321170475	0.018
A_52_P479539	Cit	1.367958161	0.452024106	9.056795522	0.050
A_52_P474242	H2-K1	1.378767451	0.463379146	7.065934196	0.010
A_51_P201187	Pllp	1.390394181	0.475493949	7.851987054	0.009
A_52_P443435	B930095G15Rik	1.391494152	0.476634845	7.499830808	0.020
A_52_P299505	Eef1a1	1.391925615	0.477082115	10.64179922	0.006
A_51_P276142	Gpr37l1	1.3919714	0.477129569	13.18304435	0.001
A_51_P400752	NA	1.40382716	0.489365321	8.877811447	0.015
A_51_P432930	Trappc3	1.410001138	0.495696327	8.098000015	0.004
A_51_P497937	Gjc2	1.410547646	0.496255399	7.149088529	0.001
A_52_P445239	Plp1	1.410645302	0.496355277	10.56198161	0.001
A_51_P496997	H2-Q10	1.430190222	0.516207045	8.944996158	0.015
A_51_P303397	Pepd	1.449040794	0.535098211	9.617289316	0.026
A_52_P1157979	Calm3	1.452656231	0.538693332	11.00685444	0.001
A_52_P318631	Eef2	1.457552595	0.543547943	10.15740779	0.004
A_51_P219789	H2-Q2	1.469389934	0.555217296	8.907647623	0.020
A_51_P262079	H2-Q7	1.47556976	0.561272128	8.319767479	0.011
A_51_P275496	BC026762	1.485969563	0.571404565	6.885703003	0.028
A_51_P304757	Gabarapl1	1.494006795	0.57918671	8.597662667	0.035
A_51_P237754	H2-T23	1.514868269	0.599192344	8.583927174	0.012

A_51_P496996	NA	1.522136355	0.606097603	8.7293637	0.029
A_52_P329451	Mbp	1.551906797	0.634041916	11.25358176	0.004
A_52_P313279	NA	1.61951588	0.695562614	9.291974672	0.015
A_52_P644972	Mzt1	1.818842169	0.863020357	7.234335262	0.002

Positive and negative values correspond to up and down regulation changes, respectively.

Table S3. qPCR data of selected genes for verification

Gene Symbol	Fold Change	p value
Glg1	1.87	0.007
Aqp4	1.45	0.011
Calca	-1.57	0.048
Eef2	2.24	0.049
Nsg1	2.04	0.047
Syt10	-2.06	0.0046

Table presents differentially expressed genes according to microarray data ($p<0.05$) evaluated by means of qPCR for verification. The genes *Glg1*, *Aqp4* and *Calca* were evaluated at the 40 days old SOD1^{G93A} pre-symptomatic mice, while *Eef2*, *Nsg1* and *Syt10* were evaluated at 80 days old SOD1^{G93A} pre-symptomatic mice. The genes were selected considering their importance in the mechanisms of cellular signaling related to neuronal trophism and plasticity with possible implications for ALS. The regulation of expression of studied genes on qPCR presents the same direction than the observed in microarray experiments (Tables S1 and S2)

Table S4. Over-represented GO terms amongst differentially expressed up or down regulated genes at 40 days old mice.

**Pathways pointed by up regulated genes**		
**Gene ID**	**Gene Symbol**	**Gene Name**
negative regulation of astrocyte differentiation		
13131	*Dab1*	disabled 1
15904	*Id4*	inhibitor of DNA binding 4
positive regulation of BMP signaling pathway		
12168	*Bmpr2*	bone morphogenetic protein receptor, type II (serine/threonine kinase)
15208	*Hes5*	hairy and enhancer of split 5 (Drosophila)
suckling behavior		
11804	*Aplp2*	amyloid beta (A4) precursor-like protein 2
14810	*Grin1*	glutamate receptor, ionotropic, NMDA1 (zeta 1)
prepulse inhibition		

14804	*Grid2*	glutamate receptor, ionotropic, delta 2
14810	*Grin1*	glutamate receptor, ionotropic, NMDA1 (zeta 1)
negative regulation of oligodendrocyte differentiation		
15208	*Hes5*	hairy and enhancer of split 5 (Drosophila)
15904	*Id4*	inhibitor of DNA binding 4
fructose 2,6-bisphosphate metabolic process		
170768	*Pfkfb3*	6-phosphofructo-2-kinase/fructose-2,6-biphosphatase 3
18640	*Pfkfb2*	6-phosphofructo-2-kinase/fructose-2,6-biphosphatase 2
positive regulation of branching involved in ureteric bud morphogenesis		
11606	*Agt*	angiotensinogen (serpin peptidase inhibitor, clade A, member 8)
22339	*Vegfa*	vascular endothelial growth factor A
positive regulation of phosphatidylinositol 3-kinase cascade		
11606	*Agt*	angiotensinogen (serpin peptidase inhibitor, clade A, member 8)
17999	*Nedd4*	neural precursor cell expressed, developmentally down-regulated 4
nucleosome assembly		
26914	*H2afy*	H2A histone family, member Y
67155	*Smarca2*	SWI/SNF related, matrix associated, actin dependent regulator of chromatin, subfamily a, member 2
72480	*Tspyl4*	TSPY-like 4
translational initiation		
217869	*Eif5*	eukaryotic translation initiation factor 5
218629	*Dhx29*	DEAH (Asp-Glu-Ala-His) box polypeptide 29
226982	*Eif5b*	eukaryotic translation initiation factor 5B
56347	*Eif3c*	eukaryotic translation initiation factor 3, subunit C
66892	*Eif4e3*	eukaryotic translation initiation factor 4E member 3
cell projection organization		
11758	*Prdx6*	peroxiredoxin 6
235442	*Rab8b*	RAB8B, member RAS oncogene family
243548	*Prickle2*	prickle homolog 2 (Drosophila)
26562	*Ncdn*	neurochondrin
382406	*Poc1b*	POC1 centriolar protein homolog B (Chlamydomonas)
78514	*Arhgap10*	Rho GTPase activating protein 10

Pathways pointed by down regulated genes

**Gene ID**	**Gene Symbol**	**Gene Name**
protein retention in ER lumen		
105785	*Kdelr3*	KDEL (Lys-Asp-Glu-Leu) endoplasmic reticulum protein retention receptor 3
carnitine metabolic process, CoA-linked		
11363	*Acadl*	acyl-Coenzyme A dehydrogenase, long-chain

fatty acid beta-oxidation using acyl-CoA dehydrogenase		
11363	*Acadl*	acyl-Coenzyme A dehydrogenase, long-chain
golgi vesicle transport		
27096	*Trappc3*	trafficking protein particle complex 3
93739	*Gabarapl2*	gamma-aminobutyric acid (GABA) A receptor-associated protein-like 2
elastic fiber assembly		
17880	*Myh11*	myosin, heavy polypeptide 11, smooth muscle
23876	*Fbln5*	fibulin 5
negative regulation of oligodendrocyte differentiation		
12387	*Ctnnb1*	catenin (cadherin associated protein), beta 1
15902	*Id2*	inhibitor of DNA binding 2
protein homotetramerization		
11363	*Acadl*	acyl-Coenzyme A dehydrogenase, long-chain
330817	*Dhps*	deoxyhypusine synthase
cartilage condensation		
12167	*Bmpr1b*	bone morphogenetic protein receptor, type 1B
12814	*Col11a1*	collagen, type XI, alpha 1
positive regulation of osteoblast differentiation		
12167	*Bmpr1b*	bone morphogenetic protein receptor, type 1B
12387	*Ctnnb1*	catenin (cadherin associated protein), beta 1
positive regulation of macrophage differentiation		
12310	*Calca*	calcitonin/calcitonin-related polypeptide, alpha
15902	*Id2*	inhibitor of DNA binding 2
negative regulation of osteoclast differentiation		
12310	*Calca*	calcitonin/calcitonin-related polypeptide, alpha
12387	*Ctnnb1*	catenin (cadherin associated protein), beta 1
regulation of cellular component organization		
16568	*Kif3a*	kinesin family member 3A
55942	*Sertad1*	SERTA domain containing 1
56213	*Htra1*	HtrA serine peptidase 1
78558	*Htra3*	HtrA serine peptidase 3

Table S5. Over-represented GO terms amongst differentially expressed up or down regulated genes at 80 days old mice.

**Pathways pointed by up regulated genes**		
**Gene ID**	**Gene Symbol**	**Gene Name**
SCF-dependent proteasomal ubiquitin-dependent protein catabolic process		
103583	*Fbxw11*	F-box and WD-40 domain protein 11
242960	*Fbxl5*	F-box and leucine-rich repeat protein 5
50754	*Fbxw7*	Fbxw7 F-box and WD-40 domain protein 7
76454	*Fbxo31*	Fbxo31 F-box protein 31
protein polymerization		
12345	*Capzb*	capping protein (actin filament) muscle Z-line, beta
22142	*Tuba1a*	tubulin, alpha 1A
22151	*Tubb2a*	tubulin, beta 2A class IIA
277360	*Prex1*	phosphatidylinositol-3,4,5-trisphosphate-dependent Rac exchange factor 1
67771	*Arpc5*	actin related protein 2/3 complex, subunit 5
post-Golgi vesicle-mediated transport		
11764	*Ap1b1*	adaptor protein complex AP-1, beta 1 subunit
11769	*Ap1s1*	adaptor protein complex AP-1, sigma 1
11840	*Arf1*	ADP-ribosylation factor 1
12757	*Clta*	clathrin, light polypeptide (Lca)
94245	*Dtnbp1*	dystrobrevin binding protein 1
tricarboxylic acid cycle		
12974	*Cs*	citrate synthase
18293	*Ogdh*	oxoglutarate (alpha-ketoglutarate) dehydrogenase (lipoamide)
66052	*Sdhc*	succinate dehydrogenase complex, subunit C, integral membrane protein
66945	*Sdha*	succinate dehydrogenase complex, subunit A, flavoprotein (Fp)
78920	*Dlst*	dihydrolipoamide S-succinyltransferase (E2 component of 2-oxo-glutarate complex)
regulation of cell shape		
104445	*Cdc42ep1*	CDC42 effector protein (Rho GTPase binding) 1
11674	*Aldoa*	aldolase A, fructose-bisphosphate
14673	*Gna12*	guanine nucleotide binding protein, alpha 12
14674	*Gna13*	guanine nucleotide binding protein, alpha 13
235611	*Plxnb1*	plexin B1
protein processing		
107522	*Ece2*	endothelin converting enzyme 2
11545	*Parp1*	poly (ADP-ribose) polymerase family, member 1
20340	*Glg1*	golgi apparatus protein 1
243853	*Fkrp*	fukutin related protein
66887	*Lonp2*	lon peptidase 2, peroxisomal

glycoprotein biosynthetic process

171212	*Galnt10*	UDP-N-acetyl-alpha-D-galactosamine:polypeptide N-acetylgalactosaminyltransferase 10
20014	*Rpn2*	ribophorin II
20443	*St3gal4*	ST3 beta-galactoside alpha-2,3-sialyltransferase 4
243853	*Fkrp*	fukutin related protein
50935	*St6galnac6*	ST6 (alpha-N-acetyl-neuraminyl-2,3-beta-galactosyl-1,3)-N-acetylgalactosaminide alpha-2,6-sialyltransferase 6
68273	*Pomgnt1*	protein O-linked mannose beta1,2-N-acetylglucosaminyltransferase

cellular response to glucagon stimulus

104111	*Adcy3*	adenylate cyclase 3
14688	*Gnb1*	guanine nucleotide binding protein (G protein), beta 1
14693	*Gnb2*	guanine nucleotide binding protein (G protein), beta 2
14701	*Gng12*	guanine nucleotide binding protein (G protein), gamma 12
14708	*Gng7*	guanine nucleotide binding protein (G protein), gamma 7
224129	*Adcy5*	adenylate cyclase 5

homophilic cell adhesion

12563	*Cdh6*	cadherin 6
19274	*Ptprm*	protein tyrosine phosphatase, receptor type, M
232370	*Clstn3*	calsyntenin 3
23836	*Cdh20*	cadherin 20
53883	*Celsr2*	cadherin, EGF LAG seven-pass G-type receptor 2 (flamingo homolog, Drosophila)
58235	*Pvrl1*	poliovirus receptor-related 1
65945	*Clstn1*	calsyntenin 1

ATP catabolic process

11305	*Abca2*	ATP-binding cassette, sub-family A (ABC1), member 2
11946	*Atp5a1*	ATP synthase, H+ transporting, mitochondrial F1 complex, alpha subunit 1
11947	*Atp5b*	ATP synthase, H+ transporting mitochondrial F1 complex, beta subunit
15481	*Hspa8*	heat shock protein 8
269523	*Vcp*	valosin containing protein
71960	*Myh14*	myosin, heavy polypeptide 14
74772	*Atp13a2*	ATPase type 13A2

endocytosis

11764	*Ap1b1*	adaptor protein complex AP-1, beta 1 subunit
11769	*Ap1s1*	adaptor protein complex AP-1, sigma 1
11771	*Ap2a1*	adaptor-related protein complex 2, alpha 1 subunit
13043	*Cttn*	cortactin
13429	*Dnm1*	dynamin 1
14269	*Fnbp1*	formin binding protein 1
16443	*Itsn1*	intersectin 1 (SH3 domain protein 1A)
16835	*Ldlr*	low density lipoprotein receptor
19261	*Sirpa*	signal-regulatory protein alpha
22174	*Tyro3*	TYRO3 protein tyrosine kinase 3

232089	*Elmod3*	ELMO/CED-12 domain containing 3
66147	*Necap2*	NECAP endocytosis associated 2
98402	*Sh3bp4*	SH3-domain binding protein 4
mRNA processing		
15382	*Hnrnpa1*	heterogeneous nuclear ribonucleoprotein A1
19655	*Rbmx*	RNA binding motif protein, X chromosome
20624	*Eftud2*	elongation factor Tu GTP binding domain containing 2
20638	*Snrpb*	small nuclear ribonucleoprotein B
224903	*Safb*	scaffold attachment factor B
233208	*Scaf1*	SR-related CTD-associated factor 1
24128	*Xrn2*	5'-3' exoribonuclease 2
53607	*Snrpa*	small nuclear ribonucleoprotein polypeptide A
53610	*Nono*	non-POU-domain-containing, octamer binding protein
54451	*Cpsf3*	cleavage and polyadenylation specificity factor 3
70465	*Wdr77*	WD repeat domain 77
70616	*Sugp1*	SURP and G patch domain containing 1
71713	*Cdc40*	cell division cycle 40
94230	*Cpsf1*	cleavage and polyadenylation specific factor 1

Pathways pointed by down regulated genes

Gene ID	Gene Symbol	Gene Name
positive regulation of nitric-oxide synthase activity		
11651	*Akt1*	thymoma viral proto-oncogene 1
16653	*Kras*	v-Ki-ras2 Kirsten rat sarcoma viral oncogene homolog
ionotropic glutamate receptor signaling pathway		
11820	*App*	amyloid beta (A4) precursor protein
14805	*Grik1*	glutamate receptor, ionotropic, kainate 1
positive regulation of BMP signaling pathway		
12168	*Bmpr2*	bone morphogenetic protein receptor, type II (serine/threonine kinase)
65960	*Twsg1*	twisted gastrulation homolog 1 (Drosophila)
endochondral ossification		
14683	*Gnas*	GNAS (guanine nucleotide binding protein, alpha stimulating) complex locus
19227	*Pthlh*	parathyroid hormone-like peptide
protein monoubiquitination		
109331	*Rnf20*	ring finger protein 20
59026	*Huwe1*	HECT, UBA and WWE domain containing 1
66105	*Ube2d3*	ubiquitin-conjugating enzyme E2D 3
chromatin remodeling		
12648	*Chd1*	chromodomain helicase DNA binding protein 1
15353	*Hmg20b*	high mobility group 20B
93761	*Smarca1*	SWI/SNF related, matrix associated, actin dependent regulator of chromatin, subfamily a, member 1

gamma-aminobutyric acid signaling pathway

14394	*Gabra1*	gamma-aminobutyric acid (GABA) A receptor, subunit alpha 1
14395	*Gabra2*	gamma-aminobutyric acid (GABA) A receptor, subunit alpha 2
14678	*Gnai2*	guanine nucleotide binding protein (G protein), alpha inhibiting 2

protein ubiquitination involved in ubiquitin-dependent protein catabolic process

226098	*Hectd2*	HECT domain containing 2
59026	*Huwe1*	HECT, UBA and WWE domain containing 1
67345	*Herc4*	hect domain and RLD 4

glycoprotein metabolic process

108155	*Ogt*	O-linked N-acetylglucosamine (GlcNAc) transferase
17156	*Man1a2*	mannosidase, alpha, class 1A, member 2
20451	*St8sia3*	ST8 alpha-N-acetyl-neuraminide alpha-2,8-sialyltransferase 3
26878	*B3galt2*	UDP-Gal:betaGlcNAc beta 1,3-galactosyltransferase, polypeptide 2

rRNA processing

14300	*Frg1*	FSHD region gene 1
66181	*Nop10*	NOP10 ribonucleoprotein
67973	*Mphosph10*	M-phase phosphoprotein 10 (U3 small nucleolar ribonucleoprotein)
69713	*Pin4*	protein (peptidyl-prolyl cis/trans isomerase) NIMA-interacting, 4 (parvulin)
73736	*Fcf1*	FCF1 small subunit (SSU) processome component homolog (S. cerevisiae)
75416	*Nop14*	NOP14 nucleolar protein
78651	*Lsm6*	LSM6 homolog, U6 small nuclear RNA associated (S. cerevisiae)

translational elongation

19951	*Rpl32*	ribosomal protein L32
19981	*Rpl37a*	ribosomal protein L37a
19982	*Rpl36a*	ribosomal protein L36A
22186	*Uba52*	ubiquitin A-52 residue ribosomal protein fusion product 1
57294	*Rps27*	ribosomal protein S27
66489	*Rpl35*	ribosomal protein L35
67945	*Rpl41*	ribosomal protein L41
71787	*Trnau1ap*	tRNA selenocysteine 1 associated protein 1

mRNA processing

19134	*Prpf4b*	PRP4 pre-mRNA processing factor 4 homolog B (yeast)
218543	*Srek1*	splicing regulatory glutamine/lysine-rich protein 1
219249	*Tdrd3*	tudor domain containing 3
328110	*Prpf39*	PRP39 pre-mRNA processing factor 39 homolog (yeast)
66354	*Snw1*	SNW domain containing 1
66373	*Lsm5*	LSM5 homolog, U6 small nuclear RNA associated (S. cerevisiae)
66637	*Tsen15*	tRNA splicing endonuclease 15 homolog (S. cerevisiae)
67684	*Luc7l3*	LUC7-like 3 (S. cerevisiae)
67797	*Snrnp48*	small nuclear ribonucleoprotein 48 (U11/U12)

68011	*Snrpg*	small nuclear ribonucleoprotein polypeptide G
68272	*Rbm28*	RNA binding motif protein 28

Protocol for astrocyte enrichment

Laser microdissected astrocytes samples were submitted to PCRs to certify about purity of samples. Primers to evaluate the presence of astrocyte, microglia and motor neuron, respectively *Gfap*, *Cd68* and *Chat*, are shown in Figure S2B. The reactions were performed to 20 µl final volume, using GoTaq Flexi DNA Polymerase (Promega), according to manufacturers and 500 nM of each primer.The protocol for PCRs consisted in 95°C during 5 minutes, followed by 35 cycles of 95°C in 30 seconds, 60°C in 30 seconds, 72°C in 45 seconds, and ending with 72°C in 7 minutes. Whole spinal cord sample was used as a positive control.

PCR products were submitted to electrophoresis in 3% agarosis gel containing ethidium bromide for 60 minutes at 100V, and then visualized under UV exposure. The result is shown in Figure S2A.

A

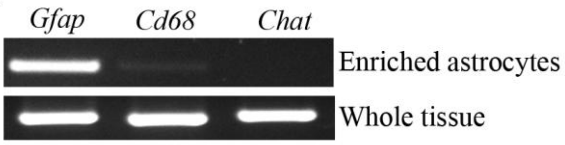

B

GeneID	Primers (5'-3')	Amplicon (bp)
Gfap	F: CAGACTTTCTCCAACCTCCAG	138
	R: CTCCTGCTTCGAGTCCTTAATG	
Cd68	F: ACTTCGGGCCATGTTTCTC	136
	R: TGGTAGGTTGATTGTCGTCTG	
Chat	F: CAAATAAGTCATAAAGGCAGAGGC	140
	R: CTCAAGGAAGACTGTGCTATGG	

Figure S2. PCR results for sample enrichment. (A) Representative bands of PCRs for specific gene markers of astrocytes (*Gfap*), microglia (*Cd68*) and motor neurons (*Chat*) in the enriched astrocyte sample and in the whole lumbar spinal cord. (B) Sequence for primers used in the PCRs and their respective amplicon sizes in base pairs (bp).
